# Mortality prediction with adaptive feature importance recalibration for peritoneal dialysis patients

**DOI:** 10.1016/j.patter.2023.100892

**Published:** 2023-12-08

**Authors:** Liantao Ma, Chaohe Zhang, Junyi Gao, Xianfeng Jiao, Zhihao Yu, Yinghao Zhu, Tianlong Wang, Xinyu Ma, Yasha Wang, Wen Tang, Xinju Zhao, Wenjie Ruan, Tao Wang

**Affiliations:** 1 Peking University, Beijing, China; 2 Centre for Medical Informatics, University of Edinburgh, Edinburgh, UK; 3 Department of Nephrology, Peking University Third Hospital, Beijing, China; 4 Department of Nephrology, Peking University People’s Hospital, Beijing, China; 5 Department of Computer Science, University of Exeter, Exeter, UK; 6 Health Data Research UK, London, UK

**Keywords:** electronic medical record, EMR, end-stage renal disease, ESRD, peritoneal dialysis, PD, mortality prediction, deep learning, model interpretability

## Abstract

The study aims to develop AICare, an interpretable mortality prediction model, using electronic medical records (EMR) from follow-up visits for end-stage renal disease (ESRD) patients. AICare includes a multichannel feature extraction module and an adaptive feature importance recalibration module. It integrates dynamic records and static features to perform personalized health context representation learning. The dataset encompasses 13,091 visits and demographic data of 656 peritoneal dialysis (PD) patients spanning 12 years. An additional public dataset of 4,789 visits from 1,363 hemodialysis (HD) patients is also considered. AICare outperforms traditional deep learning models in mortality prediction while retaining interpretability. It uncovers mortality-feature relationships and variations in feature importance and provides reference values. An AI-doctor interaction system is developed for visualizing patients’ health trajectories and risk indicators.

## Highlights

• Harnessing deep learning for PD patients’ 1-year mortality prediction
• Model clarifies medical indicators affecting mortality outcomes
• AI-doctor system visualizes individual health trajectories

## The bigger picture

As global populations age and their lifestyles change, the threat of end-stage renal disease (ESRD) grows ever more significant. Consequently, an increasing number of patients require life-sustaining treatments such as peritoneal dialysis (PD). For these PD patients, their medical journey involves more than just treatment, it is about comprehending the trajectory of their health, navigating potential health risks, and underlining the urgent need for real-time, personalized risk predictions. We employ deep learning not only to predict but also to comprehend the mortality risks associated with PD patients. Our model doesn’t merely “tell”; it “explains.” It offers clinicians insight into the reasons behind its predictions by highlighting crucial medical factors that shape these outcomes. Additionally, we created a functional AI-doctor interaction system, empowering professionals to visualize a patient’s health trajectory and grasp the personalized reference values of clinical indicators.

## Graphical abstract

**Figure:**
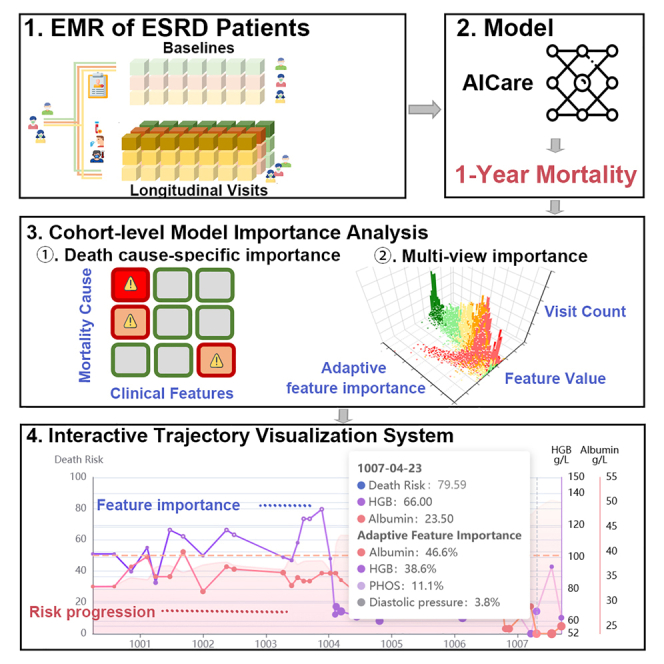

## Teaser

As global health concerns shift with aging populations, the looming threat of kidney diseases necessitates better treatment management. This study introduces an AI-driven model adept at predicting critical health risks for kidney disease patients undergoing peritoneal dialysis. By not just predicting but also explaining these risks, the research paves the way for timely, personalized medical interventions.

## Introduction

The prevalence of end-stage renal disease (ESRD) continues to increase and has become a significant healthcare burden worldwide. Approximately 3.8 million people currently rely on some form of dialysis for the treatment of ESRD worldwide.^1^ ESRD is a long-term disease, and patients need continuous medical care and treatment for years or even decades. Peritoneal dialysis (PD) is a well-established renal replacement therapy (RRT) modality and the leading form of home-based life-supporting dialysis therapy for patients with ESRD.^2^ Over the past decade, the use of PD increased dramatically worldwide.

During long-term PD, patients may still encounter various vital risks, such as cardio-cerebrovascular disease and infection.^3^ These risks may cause adverse outcomes, and patients need lifelong treatment with periodic follow-up visits to monitor their health status. Predicting mortality risk and identifying modifiable risk factors from routine clinic visit records are of great importance for personalized medicine and early intervention to prevent adverse outcomes and improve the survival of long-term PD patients. Recent studies have attempted to utilize artificial intelligence (AI) techniques to evaluate the health status of patients. However, there are still some critical issues that have not yet been thoroughly addressed by existing works.

### Issue_1_: Perform dynamic mortality prediction at each follow-up visit based on the effective utilization of both sequential medical records and the baseline demographic information

Most AI-based electronic medical record (EMR) analysis research on kidney disease patients only uses static baseline information to perform one-time health prediction based on traditional machine learning methods.^4^^,^^5^^,^^6^^,^^7^^,^^8^^,^^9^^,^^10^^,^^11^^,^^12^^,^^13^ These methods cannot perform real-time health prediction, and thus the practical utility in the clinical application is limited. Other research models the disease process by incorporating sequential EMRs.^14^^,^^15^^,^^16^ However, these models cannot yet effectively together embed the baseline information and the sequential records and capture the interaction between them during the health status embedding procedure, which leads to limited prediction performance.

### Issue_2_: Provide fine-grained interpretability for each patient individually by selecting key features that contribute the most to mortality prediction (patient-level interpretability) and simultaneously achieve high prediction performance

Key factors strongly indicate that health risk are different among patients. Medical experts need to understand how a model makes a specific decision for a particular patient. This requires sufficient model interpretability to ensure that prediction results are trustworthy for developing bespoke interventions and extracting medical knowledge. However, most existing studies fail to ensure the model’s trustworthiness in providing verifiable interpretations. On one hand, traditional machine learning models, such as decision trees,^7^^,^^8^^,^^11^^,^^12^^,^^17^ are clinically interpretable, but they cannot capture complex longitudinal progressions and thus have inferior prediction performances. On the other hand, the decision-making process in deep learning-based models is a black box and fails to provide human-understandable interpretation.^13^^,^^14^ Some recent works apply the Shapley additive explanation (SHAP),^18^^,^^19^ feature permutation,^9^^,^^10^ and inverse analysis^20^ strategies to improve the interpretability. However, these post hoc interpretation^21^ methods can only provide coarse-grained interpretability, which is difficult to understand at the patient level. It is still challenging to simultaneously provide satisfactory interpretability and achieve high prediction performance.

### Issue_3_: Adaptively analyze the importance of each feature along with the variation of its value (feature-level interpretability) to provide medical advice and extract knowledge

The way of attending to the medical feature in the prediction process should be flexible and individualized according to its value. However, most existing studies analyze the health status of patients in a fixed decision process^7^^,^^12^^,^^17^ or embed clinical features via fixed parameters of neural networks without ante hoc interpretability.^9^^,^^13^^,^^20^ To the best of our knowledge, none of the existing AI-based clinical prediction studies for kidney disease patients explicitly analyze the changes in the feature importance with features’ values.

**Figure 1:**
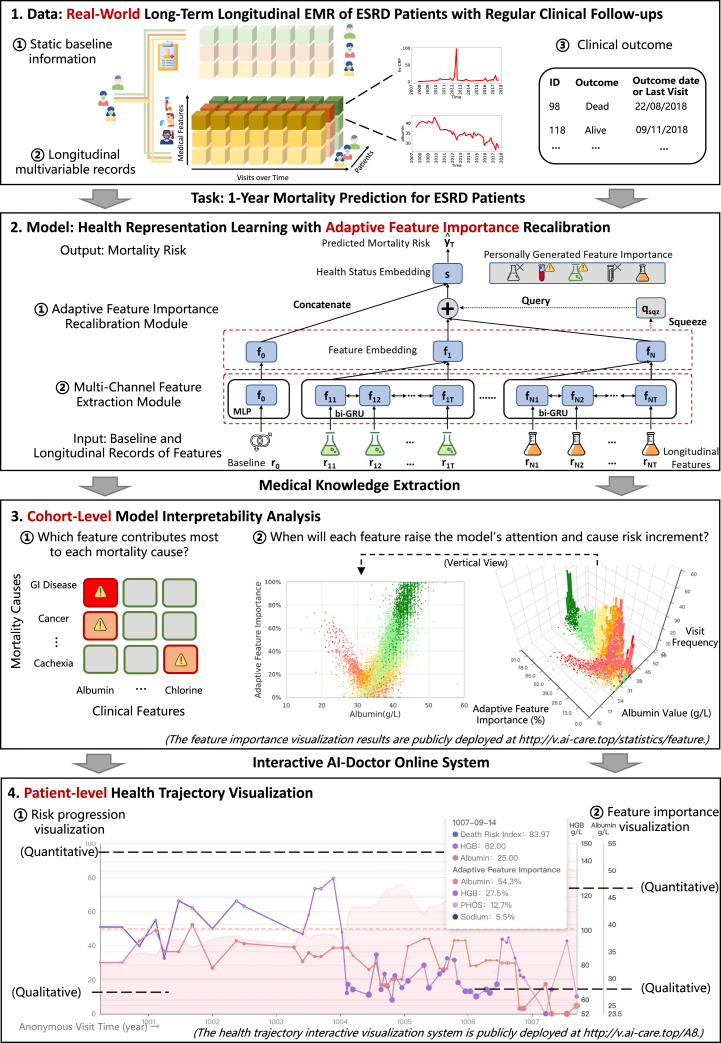
Mortality prediction research overview for peritoneal dialysis (PD) patients (1) We collect an over 12-year, long-term, and real-world clinical EMR dataset of PD patients, consisting of static baseline information, longitudinal multivariable records, and clinical outcomes. The prediction task is defined as a 1-year mortality prediction at each clinical visit. (2) We propose a deep learning-based interpretable health status representation learning framework consisting of a multichannel feature extraction module and an adaptive feature importance recalibration module. (3) We perform a model interpretability analysis for diverse mortality causes and observe the change of feature importance to extract novel medical knowledge (taking albumin as an example). (4) We build an interactive AI-doctor system to visualize the health trajectory.

To address these challenges, we propose a deep learning-based interpretable mortality risk prediction framework for PD patients, AICare. As shown in Figure 1, it is built upon a real-world longitudinal EMR dataset of PD patients spanning 12 years, including baseline demographic information and outcomes, as well as patient-level follow-up lab tests and diagnosis records spanned by an average of 20 visits per patient. The main contributions of this work are summarized below.

**Figure 2:**
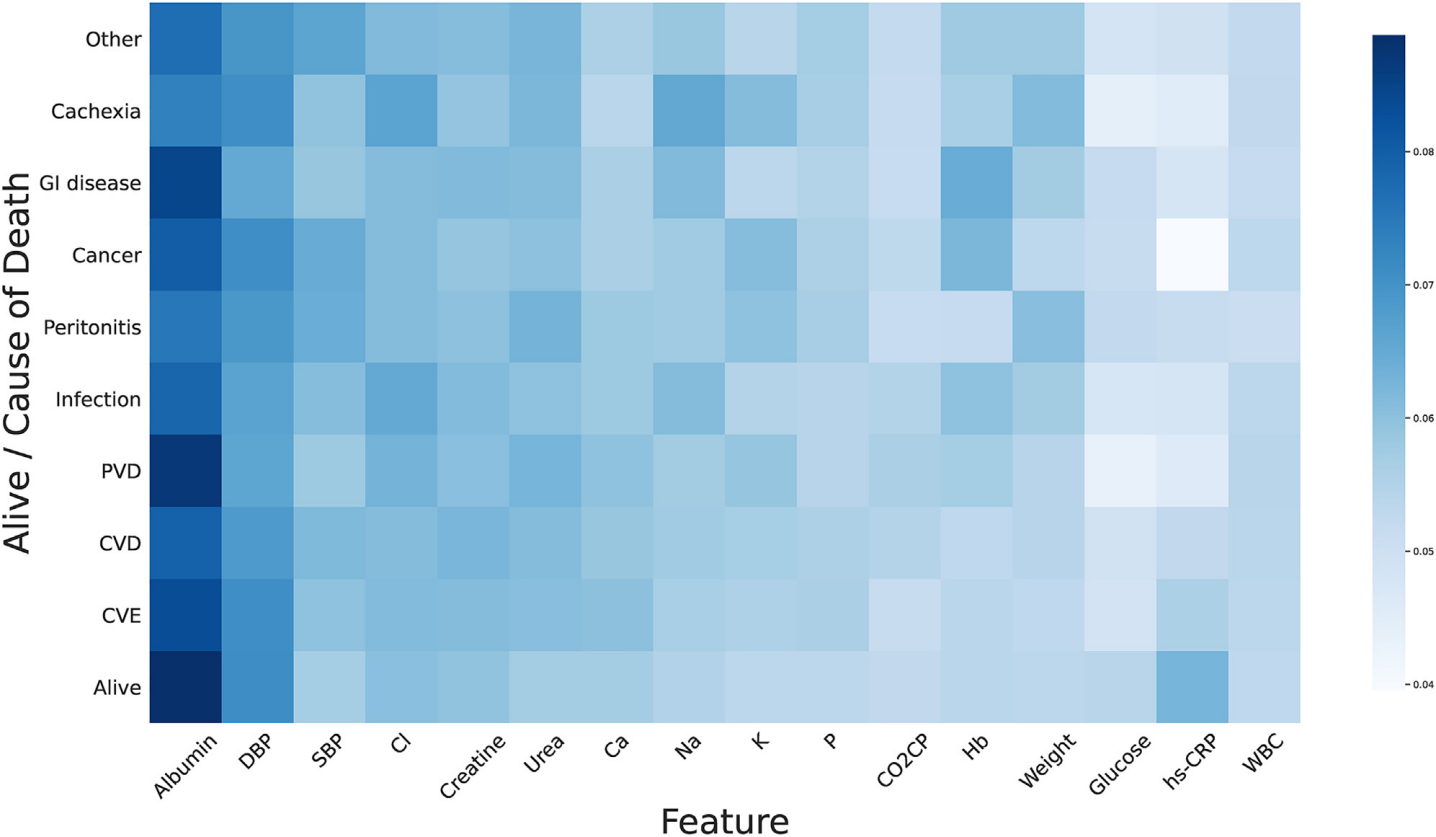
Average feature importance heatmap for diverse mortality causes generated by AICare The darker the color, the greater the importance. Serum albumin is the most important feature in mortality prediction, especially for PD patients who died of gastrointestinal (GI) disease or peripheral vascular disease (PVD). Diastolic blood pressure (DBP) is the second indicative feature, especially for PD patients who died of cachexia, cancer, or cerebrovascular disease (CVE).
(3) AICare reveals the variation pattern in each feature’s importance for PD patient mortality prediction (feature-level interpretability in ${\mathrm{Issue}}_{3}$). As shown in Figure 1 section 3., Figures 3 and 4, and Table 5, AICare provides the ante hoc attention weight of each clinical feature according to its value and the patient’s condition. We report detailed feature-level interpretability analyses. There are two variation patterns of importance in medical features: V-shaped parabolic curves (e.g., albumin and DBP) and L-shaped fold lines (e.g., SBP and hemoglobin). For example, the importance weight of albumin is presented as a V-shaped curve with 32 g/L as the lowest turning point. For most PD patients, when albumin is lower (higher) than the turning point of 32 g/L, the more extreme the value, the more attention weight is assigned by AICare, which means that this feature plays an essential role in the health status representation learning, and the predicted mortality risk rises (declines). AICare recommends improving albumin to higher than 32 g/L—the higher the better. The importance weight of SBP presents as an L-shaped curve with 130 mm Hg as a turning point. For SBP over 130 mm Hg, AICare pays nearly no attention to SBP. AICare recommends raising the SBP to at least 130 mm Hg for most PD patients, but a further increase in SBP will not bring many benefits.

(1) Our proposed framework, AICare, models the health trajectory based on multivariate EMR data of PD patients and achieves better prediction performance than state-of-the-art (SOTA) methods while simultaneously providing fine-grained patient-level interpretability. As shown in Figure 1 section 2, AICare employs a multichannel medical feature embedding architecture to extract sequential patterns from high-dimensional medical features. The squeezed embedding of static information and dynamic features is treated as a health context vector to guide the feature importance recalibration (addressing ${\mathrm{Issue}}_{1}$). AICare assigns attention weights for each feature by looking at the health context for clues that can help lead to a more individual representation of the health status. On the dynamic mortality prediction tasks, AICare achieves 47.2% AUPRC (area under the precision-recall curve) on the PD dataset, which is 11.8% relatively higher than the SOTA comparative baseline model. We also introduce an additional experiment dataset, which is described in the supplemental information.
(2) AICare provides an elucidation of the relationship between the causes of mortality in patients with PD and clinical features (patient-level interpretability in ${\mathrm{Issue}}_{2}$) based on an end-to-end deep learning model. As shown in Figure 1 section 3.1 and Figure 2, AICare achieves fine-grained interpretability by adaptively emphasizing high-risk features during the prediction process based on a feature recalibration module. We report detailed patient-level interpretability analyses; serum albumin, diastolic blood pressure, and chlorine are the most important indicators for most PD patients. Albumin is a strong indicator for patients who died of gastrointestinal disease and peripheral vascular disease. Diastolic blood pressure (DBP) is an indicator for patients who died of cachexia, cancer, and cerebrovascular disease. Systolic Blood Pressure (SBP) is indicative of cancer and PD-associated peritonitis deaths.

**Figure 3:**
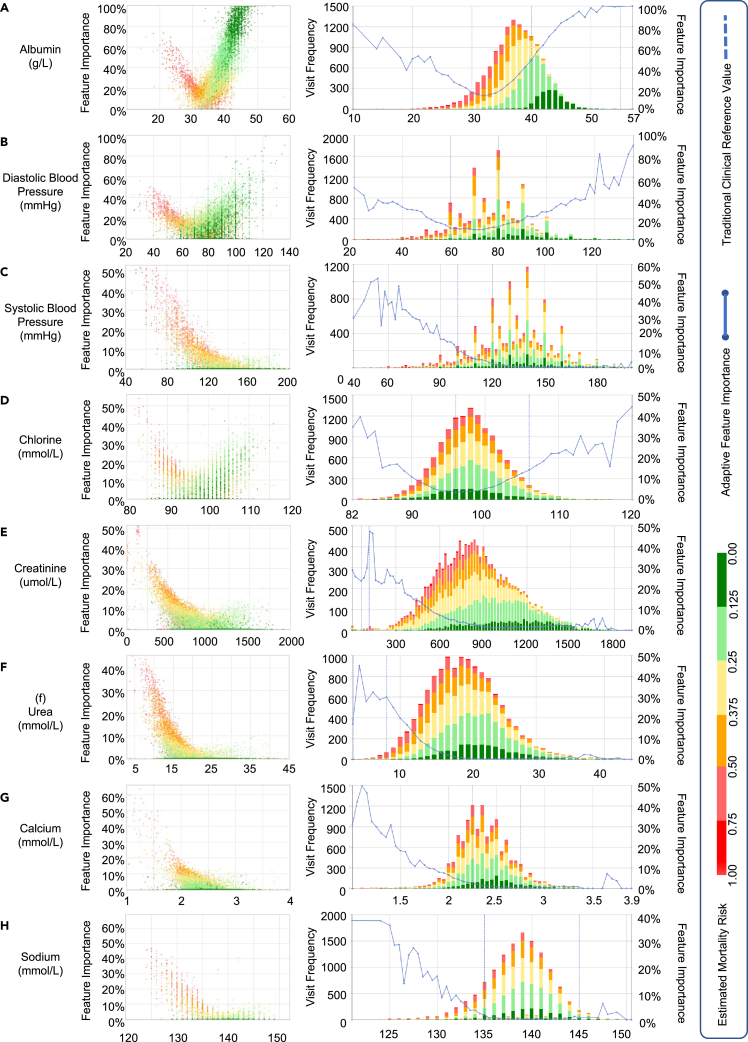
Feature importance variation learned by AICare (features a–h) The clinical visits are marked as colored dots and histograms. Red represents high risk predicted by AICare, while green represents low risk. The average feature importance is visualized as blue fold lines. The traditional clinical reference values are vertically marked as blue dotted lines. There are two variation patterns of feature importance: V-shaped parabolic curves (e.g., albumin, DBP) and L-shaped fold lines (e.g., SBP and Hb). We take the serum albumin importance variation as an example of a V-shaped parabolic curve. For most patients, when albumin is lower (higher) than the turning point of 32 g/L, the more extreme the value, the more attention weight is assigned by AICare, which means this feature takes essential part in health status representation learning, and the predicted mortality risk rises (declines). As a result, AICare recommends improving the serum albumin to above 32 g/L—the higher the better. On the contrary, we take the systolic blood pressure (SBP) importance variation as an example of an L-shaped fold line. For most patients, when SBP is lower than the turning point of 130 mm Hg, the lower the value, the more attention weight is assigned. However, when SBP is higher than 130 mm Hg, the attention weight drops to nearly 0%, meaning this feature will no longer affect the representation learning of health status. As a result, AICare recommends improving the SBP to at least 130 mm Hg, but higher SBP will not bring many benefits.

**Figure 4:**
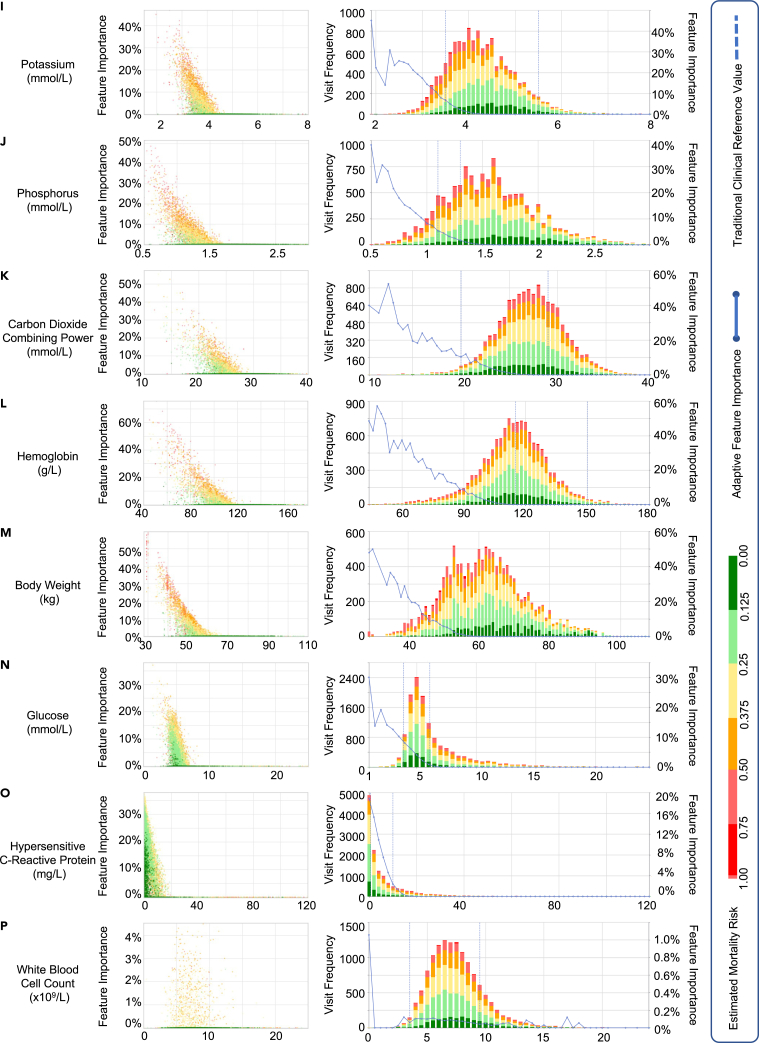
Feature importance variation learned by AICare (features i–p)
(4) We develop a practical AI-doctor interaction system to visualize the trajectory of patients’ health status and risk indicators. Model deployment has been the last but most challenging step toward clinical application. Deploying the deep models in an accessible way for clinicians to allow them to easily understand model predictions and model decision process still needs extra considerations. As shown in Figure 1 section 4, to further facilitate personalized clinical service, we deploy an AI-doctor interaction system online with open-source code at https://github.com/Accountable-Machine-Intelligence/AICare. Our developed health trajectory visualization system with anonymous case studies (patient IDs A1–A20) is publicly available at http://v.ai-care.top/A8. Visualization of the importance of the features is available at http://v.ai-care.top/statistics/feature. Users can upload the data online to get the prediction results immediately (http://v.ai-care.top/predict) or download the code to train the model based on their dataset offline. We made an abstract presentation video introducing our work (https://youtu.be/CY2glHchsC8).

## Results

### Data description and problem formulation

We collected the EMR data of 656 PD patients with 13,091 visit records, spanning over 12 years, from January 1, 2006, to January 1, 2018, including patients’ baseline data, longitudinal visit records, and outcomes.

(1) Baseline data include patients’ demographic data (e.g., age and gender) and the diagnosis of diabetes at the beginning of dialysis. The statistics of the baseline data and the assignment of the labels are shown in Table 1.

**Table 1:** Statistics of baseline information and label assignment

	Total	Mortality (%)	Survival (%)
Patients	656	261 (39.8%)	395 (60.2%)
Visits	13,091	1,196 (9.1%)	11,895 (90.9%)

Age			

16–40	96 (14.6%)	10 (10.4%)	86 (89.6%)
40–60	217 (33.1%)	64 (29.5%)	153 (70.5%)
60–80	297 (45.3%)	153 (51.5%)	144 (48.5%)
80–98	44 (6.7%)	33 (75.0%)	11 (25.0%)

Diabetes			

Patients with diabetes	244 (37.2%)	120 (49.2%)	124 (50.8%)

Gender			

Female	327 (49.8%)	125 (38.2%)	202 (61.8%)
Male	328 (50.2%)	136 (41.5%)	192 (58.5%)

The real-world dataset contains 656 peritoneal dialysis (PD) patients with 13,091 clinical visits. There are 39.8% patients who died before the final follow-up. The age range of patients enrolled is 16–98 years.
(2) Visit data include laboratory tests and patients’ vital signs at each visit. The visit frequency statistics and the feature values distribution are shown in Tables 2 and 3, respectively.

**Table 2:** Statistics of age and visit frequency

Statistic	Avg	Med	Max	Min	Std
Age (year)	58.55	60.70	97.45	16.79	15.81
Visits per patient	19.95	16	69	1	13.53
High-risk visits per patient	2	0	29	0	2.95
Duration of follow-up (year)	3.98	3.43	10.44	0.1	2.67
Visit interval (month)	2.73	2.48	29.87	–	2.67

Peritoneal dialysis (PD) patients were followed up every 3 months. There are about 20 visits recorded for each patient. Avg, average; Med, median; Max, maximum; Min, minimum; Std, standard deviation.

**Table 3:** Feature summary of the peritoneal dialysis (PD) dataset

Abbreviation	Full name	Unit	High-risk visits (y=1)			Low-risk visits (y=0)			Missing
Dynamic Features			Mean	Std	Med	Mean	Std	Med	
Albumin	albumin	g/L	33.81	4.437	34.3	37.87	4.337	38	25%
DBP	diastolic blood pressure	mm Hg	70.28	14.71	70	78.59	13.79	80	18%
SBP	systolic blood pressure	mm Hg	125.3	25.19	127	134.4	21.61	135	14%
Cl	chlorine	mmol/L	96.02	4.155	96	98.21	4.923	98	17%
Cr	creatinine	μmol/L	779.6	250.3	741	868.9	270.3	853	10%
Urea	urea	mmol/L	18.12	5.545	17.8	20.09	5.363	19.8	11%
Ca	calcium	mmol/L	2.358	0.277	2.345	2.406	0.341	2.39	12%
Na	sodium	mmol/L	137.1	4.262	137.9	138.5	4.617	139	21%
K	potassium	mmol/L	4.240	0.783	4.17	4.320	0.718	4.25	11%
P	phosphorus	mmol/L	1.549	0.450	1.5	1.606	0.430	1.57	13%
CO_2_ CP	CO_2_ combining power	mmol/L	27.45	3.562	27.5	27.38	3.630	27.4	8%
Hb	hemoglobin	g/L	111.4	19.54	113	114.6	17.05	115	12%
Weight	body weight	kg	59.98	11.05	59.59	62.26	11.07	62	41%
Glucose	glucose	mmol/L	7.758	3.665	6.7	6.689	3.089	5.7	30%
hs-CRP	hypersensitive C-reactive protein	mg/L	17.57	28.07	8.49	7.954	13.96	3.19	29%
WBC	white blood cell count	×10^9^/L	8.238	2.767	7.895	7.773	2.754	7.43	10%

Baseline features									

Age	age	year	66.12	13.01	67.82	53.30	15.54	54.53	0%
Gender	female (0) or male (1)	–	0.53	0.50	1	0.49	0.50	0	0%
Height	height	cm	162.2	9.95	160.5	164.1	10.98	163.8	0%
Diabetes	is (1) or is not (0) diabetic	–	0.45	0.50	0	0.31	0.46	0	0%

This dataset comprises 16 dynamic features recorded at each clinical visit and 4 static baseline features recorded at the first visit. Med, median; Std, standard deviation.
(3) Outcome data include patients’ outcomes at the end of the data collection window, including death date and cause of death (e.g., cancer). The outcomes of all patients were followed and further recorded until October 31, 2018.

The feature sets consist of 16 longitudinal medical features and 4 baseline features. The age distribution of the patients is 58.55 ± 15.81 years, and the number of average records per patient is 19.95 ± 13.53. We fill in the missing values with the most recent historical recorded values.

We conduct the 1-year dynamic mortality prediction task. Given a patient’s visit records with *T* visits, the binary classification task is to predict the mortality risk in the future 1 year ${\hat{y}}_{t}$ at each visit *t*. To meet actual clinical practice, we also define an uncertain phase of patient health status. For patients with negative labels (alive, y=0), the uncertain phase is 1 year before the end date of data collection because we do not know the outcomes of these patients in the future 1 year. For patients with positive labels (dead, y=1) at *t*, the uncertain phase is between the t−2 year and the t−1 year because we are uncertain about the ground-truth health status during these visits. The final dataset contains 1,196 visits with positive labels (i.e., died within 1 year) and 10,804 records with negative labels. For more details about the dataset and the problem formulation, please see the supplemental information.

### Prediction performance

The prediction performance of AICare and the baseline models on the 10-fold cross-validation mortality prediction of PD patients, evaluated by AUPRC and area under the receiver operating characteristic curve (AUROC), are shown in Table 4. AICare achieves 47.2% AUPRC, which is relatively 11.8% higher than the best baseline model.^1^ This indicates that AICare can efficiently embed the long-term longitudinal multivariable sequential data and static baseline data to learn the representation of the health status of PD patients individually, using the multichannel feature extraction module and the adaptive feature importance recalibration module. More details about the experiment are listed in the supplemental information, including the prediction performance of diverse mortality causes and detailed descriptions of the comparative baseline methods. To verify the application universality of AICare on other patient cohorts, we also introduce an additional experiment dataset to train the model and test the prediction performance, which consists of 1,363 ESRD patients receiving hemodialysis (HD) from another grade A tertiary hospital. The prediction performance is listed in the supplemental information.

**Table 4:** Mortality prediction performance of PD patients

Method	AUPRC	AUROC
GRU (gated recurrent unit)^22^	0.422 (0.109)	0.781 (0.047)
Transformer^23^	0.406 (0.097)	0.789 (0.047)
MT-RHN (multi-task recurrent highway network)^15^	0.413(0.107)	0.777(0.063)
LSTM (long short-term memory network)^19^	0.395(0.100)	0.782(0.065)
biLSTM-FC (bidirectional LSTM with fully connected layer)^24^	0.398(0.089)	0.758(0.067)
LR (logistic regression)^5^	0.370 (0.084)	0.610 (0.044)
XGBoost^17^	0.379 (0.087)	0.597 (0.033)
DT (decision tree)^12^	0.319 (0.040)	0.607 (0.027)
LightGBM^18^	0.405 (0.082)	0.604 (0.028)
AICare	0.472 ^∗∗^ (0.075)	0.816 ^∗∗^ (0.033)

Our proposed model, AICare, outperforms other comparative baseline approaches, including deep models. The values in parentheses are the standard deviation of 10-fold cross-validation. ^∗∗^: p<0.01.

### Interpretability analysis

AICare provides fine-grained interpretability to help clinicians understand the prediction decision process. At each visit, the model provides dynamic importance weights for the features that indicate the contributions of each feature to the final prediction result. In this section, we discuss the detailed interpretability analyses.

#### Average feature importance for diverse causes of death

We calculate the average importance of each feature for patients, which is shown as a heatmap in Figure 2. The results indicate that serum albumin, DBP, and chlorine (Cl) are considered important health indicators for most PD patients because their columns are darker than other feature columns. Some findings of the relationship between the causes of mortality in PD patients and clinical features are listed below.

Albumin is the strongest indicator of most causes of death, especially for cerebrovascular disease (CVE), peripheral vascular disease (PVD) and gastrointestinal (GI) disease, according to the heatmap generated by AICare. This may be because albumin is an indicator of protein energy wasting, correlated with suboptimal GI intake and inflammation.^25^^,^^26^ Hypoalbuminemia is a strong predictor for PD-related peritonitis,^27^ which is the primary reason for deaths from infection and peritonitis. Besides, our model generates a high attention weight of albumin for patients who are still alive, which means that low-risk scores are associated with high albumin value. More details about albumin can be found in the supplemental information.

DBP is a risk indicator for CVE, PD-related peritonitis, cancer, and cachexia deaths. This may be because DBP is a marker of atherosclerosis and is strongly independently related to atherothrombotic brain infarction incidence.^28^ Low DBP could also be an indicator for low peripheral vascular resistance or increased arterial stiffness^29^^,^^30^ that is strongly associated with a high incidence of cardio-cerebral vascular disease.^31^ Additionally, low blood pressure (BP) is a surrogate predictor for specific comorbidities, heart failure, chronic inflammation, and malnutrition,^32^ which may be related to death from peritonitis, cancer, and cachexia.

Sodium (Na), potassium (K), and body weight are important indicators for cachexia deaths. This may be because patients with cachexia often experience low Na and K levels due to insufficient food intake. Decreasing weight for these patients is a common phenomenon.

Hemoglobin (Hb) is an important indicator for GI disease deaths. GI bleeding is a critical manifestation of uremic GI disease. Hb and K are indicators for cancer deaths, which are consistent with the fact that cancer is highly associated with refractory anemia, anorexia, and, consequently, hypokalemia due to insufficient intake.

Urea, body weight, K, albumin, DBP and SBP are important indicators for PD-related peritonitis deaths. The risk factors for peritonitis, a common complication of PD patients, have been well defined^27^ and include hypoalbuminemia, hypokalemia, protein energy wasting, etc. This is consistent with the results of our model.

#### Change of feature importance with feature values

AICare quantifies the feature importance changes with feature values in a macroscopic perspective for the whole patient cohort to help clinicians better understand the decision process, perform individualized intervention, and extract new medical knowledge, as shown in Figures 3 and 4.

In the left scatterplot, the x axis denotes the value of the biomarker. The y axis denotes the feature’s importance. Each dot represents a follow-up visit of a patient, and the color represents the predicted risk. The right histogram shows the risk distributions at different values of biomarkers. The blue curve is the fitted curve of the average importance of the feature. We also plot each feature’s traditional clinical reference ranges for normal clinic outpatients as blue dotted lines, helping physicians evaluate the consistency between the results of AICare and the traditional ranges.

There are two obvious patterns of relationships between biomarkers’ importance weights and recorded values: a V-shaped parabolic curve and an L-shaped fold line. For the V-shaped parabolic pattern (e.g., albumin and DBP), an extremely high or low feature value will cause high importance attention weight through AICare, which means the feature plays an essential part in the learning the representation of health status. For the L-shaped fold line pattern (e.g., SBP and Hb), the lower the biomarker value, the higher the importance of attention weight.

The pattern of variation in importance and the recommended reference values learned by AICare are summarized in Table 5. We will discuss these patterns in detail in the following text.

(1) Albumin (Figure 3A). AICare believes that the albumin importance attention weight appears to be a V-shaped curve with 32 g/L as a turning point. The variation of albumin in a descending or ascending manner always gets the model’s attention. Considering that the red dots are mostly on the left side of the figure, AICare learns that patients with an albumin level lower than 32 g/L tend to have a high importance weight and poor prognosis (y>0.5). When the albumin level is lower than 23 g/L, more than 50% attention weight is given, which means that the albumin level becomes the most critical indicator for the 1-year mortality outcome.

**Table 5:** Importance variation pattern and recommended reference value (turning point) learned by AICare for PD patients

Feature	Unit	Importance variation learned by AICare			Traditional reference range		Consistency
		Variation type	Recommendation	Turning point	Lower limit	Upper limit	
Albumin	g/L	V shape	higher	>32	40	55	√
DBP	mm Hg	V shape	higher	>70	60	80	∼
SBP	mm Hg	L shape	at least	>130	100	120	×
Cl	mmol/L	V shape	higher	>96	96	106	√
Cr	μmol/L	L shape	at least	>900	62	115	×
Urea	mmol/L	L shape	at least	>20	3.1	9	×
Calcium	mmol/L	L shape	at least	>2.5	2.25	2.75	∼
Na	mmol/L	L shape	at least	>135.5	135	145	√
K	mmol/L	L shape	at least	>4	3.5	5.5	√
P	mmol/L	L shape	at least	>1.5	1.1	1.3	×
CO2CP	mmol/L	L shape	at least	>25	20	29	∼
Hb	g/L	L shape	at least	>114	115	150	√
Weight	kg	L shape	at least	>59	–	–	–
Glucose	mmol/L	L shape	not exceed	<6	3.9	6.1	√
Hs-CRP	mg/L	L shape	not exceed	<16	0.5	10	√
WBC	×10^9^/L	irregular	unknown	–	3.5	9.5	–

This table is a quantified summary of Figures 3 and 4. Recommendation “higher” means that AICare suggests increasing this feature’s value above the turning point. “At least” means that AICare suggests maintaining the value above the turning point, but a further increase may not bring many benefits. Consistency (∼) means that there is some overlap between the reference range recommended by AICare for PD patients and the traditional reference range for outpatients. We have publicly deployed the visualization charts of the variation in importance of features at http://v.ai-care.top/statistics/feature.

On the other hand, between the range of 32 and 57 g/L, a high albumin value also causes high importance weight and indicates a significant improvement in the patient’s health (y<0.5). When the albumin level is higher than 40 g/L, it often occupies about 50% to even 100% of the feature importance weight, which means the model can predict the high survival expectation of patients using just this feature. As a result, AICare recommends raising the albumin level to above 32 g/L as much as possible for most PD patients.

The traditional clinical reference range of albumin for outpatient clinics is 40–55 g/L, which is highly consistent with the recommended range given by AICare. This finding is also consistent with a recent study that evaluated the association between serum albumin trajectories and mortality in PD patients using the joint modeling approach, showing that changes (increases and decreases) in serum albumin over time were strongly and significantly associated with mortality after adjustment for the risk factor.^33^.

(2) DBP (Figure 3B). DBP is another critical feature in the evaluation of patient health status. Similar to albumin, both high and low levels of DBP will affect the model’s attention. The importance weight of DBP varies in a V-shaped curve with 70 mm Hg as a turning point. In the 40–70 mm Hg range, the model pays more attention to DBP as it gets lower and predicts a poor prognosis. When the DBP is below 40 mm Hg, it takes more than 30% of the model attention weights. Most patients whose DBP is below 60 mm Hg are more likely to have a high health risk, marked as red dots in the figure.

On the other hand, in the range of 70–120 mm Hg, the model pays more attention as the DBP gets higher and predicts better prognosis outcomes. When DBP is above 85 mm Hg, it also occupies about 20% of model attention weights, and patients are predicted to have a low-risk condition for most cases, marked as green dots in the figure. As a result, AICare recommends increasing the DBP to above 70 mm Hg, while a greater DBP indicates lower risk.

This is consistent with recent studies of dialysis patients. Higher DBP was associated with decreased early mortality in the first year after the start of RRT.^34^ All-cause mortality risk was minimal at 77 mm Hg for DBP in 7,335 Chinese PD patients.^35^ DBP lower than 70 mm Hg may be related to an increased mortality risk in both nondiabetic patients with chronic kidney disease and HS patients.^35^^,^^36^^,^^37^^,^^38^ The traditional normal reference range of DBP for outpatient clinics is 60–80 mm Hg, while maintaining DBP at a relatively higher level is conducive to improving the survival of PD patients. Further research about DBP for PD patients is needed.

(3) SBP (Figure 3C). Unlike the features discussed above, AICare believes that SBP is a typical feature whose importance weights vary in an L-shaped fold line with 130 mm Hg as a turning point, meaning that the importance weights decrease as the value increases. For SBP below 60 mm Hg, AICare gives more than 50% attention, and in most cases, patients are likely to be predicted to have poor outcomes, presented as red dots in the figure. For SBP over 130 mm Hg, AICare pays nearly no attention to SBP (α<1%), which means that SBP does not affect health status representation learning. As a result, AICare recommends maintaining the SBP at 130 mm Hg or slightly higher for most PD patients. A further improvement over 130 mm Hg does not significantly help reduce mortality risk.

This is consistent with clinic experience and most of the recent studies. Lower BP was a surrogate marker for severe comorbid conditions (e.g., heart failure or ischemic heart disease), chronic inflammation, and malnutrition and, hence, can lead to worse outcomes by limiting blood flow to vital organs.^35^^,^^39^ The traditional reference range of SBP for outpatient clinics is 100–120 mm Hg. Although accepted definitions of hypertension and BP treatment targets in the dialysis population have not been determined, and definitive recommendations regarding BP treatment targets in dialysis patients have not been made, it is clear that hypotension should be avoided.^40^

(4) Creatinine (Cr) (Figure 3E). The importance variation curve of serum Cr is also L shaped, similar to SBP. For Cr levels in the 160–900 μmol/L range, the lower the level, the more attention is paid by AICare. When the Cr level drops below 400 μmol/L, the model provides more than 15% of attention weights, and the patients are likely to face a poor prognosis (y>0.5). For serum creatinine levels in the range of 900–1750 μmol/L, it often only occupies 5% of attention weights, and patients in this range generally have a lower mortality risk (y<0.5). As a result, AICare recommends maintaining the Cr level at least 900 μmol/L or slightly higher for most PD patients.

This is consistent with the finding of a previous study that a low Cr level (707–815 μmol/L as reference) as a proxy of low muscle mass, nutritional status, and protein energy wasting (PEW) may be associated with adverse outcomes in PD patients.^41^^,^^42^ In contrast, a high Cr level is associated with a relatively lower mortality risk.^41^ Cr should be maintained at a certain level. The traditional reference range of Cr for normal outpatient clinics is 62–115 μmol/L, which is unsuitable for PD patients. Note that AICare provides a rough recommendation for most PD patients in this dataset. We will specify this finding for different cohorts (e.g., different gender) in future work.

(5) Hb (Figure 4L). The curve of the importance variation of Hb is L shaped. The model pays more attention to the Hb level at 44–114 g/L as the Hb level decreases. Hb occupies about 20%–60% of the model attention weights when the Hb level is below 100 g/L. Patients in this range are more likely to have a high mortality risk. The model pays almost no attention to Hb levels above 114 g/L. As a result, AICare recommends keeping Hb levels at least 114 g/L, but further increases in Hb may not bring many benefits.

As indicated by a previous study, an Hb level lower than 100 g/L was significantly associated with a higher risk for all-cause and cardiovascular deaths.^43^ Lower Hb is also associated with a higher mortality risk in ESA (erythropoiesis-stimulating agents)-treated PD patients.^44^ Current anemia management guidelines also suggest not using ESAs to maintain a Hb concentration above 110 g/L in adults.^45^

More analyses about the features’ importance are listed in the supplemental information.

### Case studies with the health trajectory interactive visualization system

To intuitively show the prediction process and verify the reasonability of AICare when applied to clinical practice, we develop an online AI-doctor system with an interactive interface to visualize the patient’s health trajectory with the importance weights of features at each time step. This system makes the prediction results of deep learning models more accessible to clinicians and helps physicians make individualized clinical decisions. We draw a two-dimensional line chart to show the changes in the patient’s biomarkers. The x axis is the visit timeline, and the y axis is the value of biomarkers. At each time step, we plot the predicted risk curve ${\hat{y}}_{i}$ (values from 0%–100%). The attention weights of different biomarkers at each time step are also visualized, symbolized as the size of each data point on the line chart. The larger the point, the higher the attention weight.

In the following, we analyze two patient cases using our system. The clinical visit dates were reset to start in 1000 (year) on the online visualization system to protect privacy.

**Figure 5:**
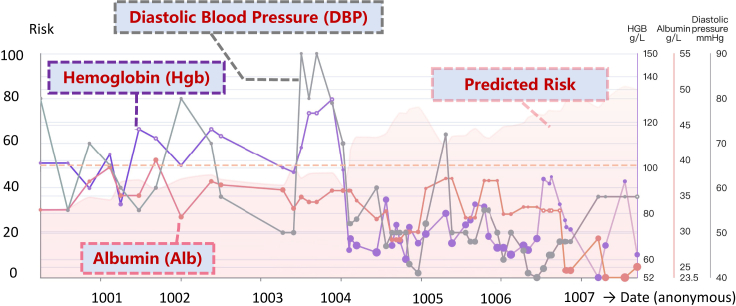
Case study I: Patient died of multiple organ failure Mortality risk prediction results and interpretability analysis are deployed on a health trajectory interactive visualization system. The x axis denotes the visit date. The y axis denotes the predicted mortality risk (visualized as a pink translucent curve) and feature values. AICare provides the features’ importance weights as interpretability at each visit, symbolized as the size of each data point on the line chart and also listed in the inset labels. AICare pays most attention to albumin, Hb, and DBP for this patient, and the patient died of multiple organ failure. The health trajectory interactive visualization system is publicly deployed at http://v.ai-care.top/A8 and is available in English and simplified Chinese.

Case I Patient died of multiple organ failure (Figure 5): the first patient died in 1007 (year) due to prostate cancer and multiple organ failure. Figure 5 shows the patient’s risk prediction and historical visit information.

During the period the red dotted box covers in the figure, AICare kept predicting a high risk 3 years before the adverse outcome. AICare mainly focused on albumin, DBP, and Hb due to their abnormal values and declining patterns. It is evident that the values of Hb and DBP decreased sharply at the beginning of 1004, which decreased by 69 g/L (from 130 g/L to 61 g/L) and 27 mm Hg (from 79 mm Hg to 52 mm Hg), respectively. AICare sensed the changes rapidly and started to pay attention to them. There was 31.0% of attention given to Hb and 19.8% given to DBP. We can also find a sudden drop of albumin from 32.9 mmol/L to 24.5 mmol/L in 1007 (year), and the albumin level remained at a low level during the last several visits since then, which kept drawing 30%–40% of attention weights of our model.

According to the records, this patient had a series of comorbidities since 1004, including unstable angina pectoris, peripheral arterial disease (PAD), prostate cancer, anemia, diabetic foot, and inflammatory bowel disease, which were closely related to the abnormal biomarkers identified by AICare.

Specifically, the decline of DBP indicated worsening arterial stiffness, which may be associated with severe atherosclerosis, such as coronary heart disease, PAD, and diabetic foot in this patient. The abnormal Hb level indicated deleterious anemia and could be associated with GI bleeding, severe infection, malnutrition, prostate cancer, diabetic foot, and inflammatory bowel disease.^25^^,^^26^^,^^46^ With the help of AICare, physicians may be reminded early to perform a further examination to confirm and treat the these conditions accordingly.

**Figure 6:**
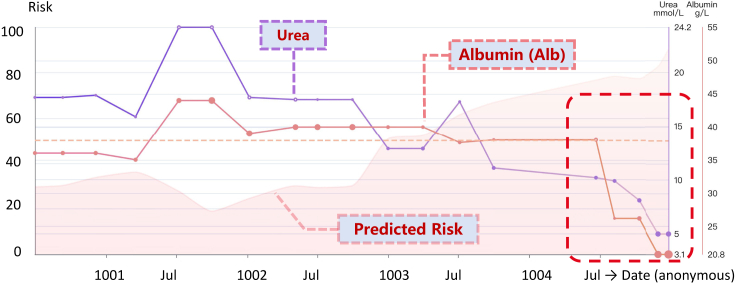
Case study II: Patient died of digestive system disease AICare pays most attention to albumin and urea for this patient. The patient died of digestive system disease. The health trajectory is shown at http://v.ai-care.top/A2.

Case II Patient died of digestive system diseases (Figure 6): the second patient was diagnosed with ischemic kidney disease, and PD therapy was initiated. This patient died in December 1004 due to GI disease.

Since July 1003, the risk score generated by AICare increased continuously increased. The attention varied but focused mainly on serum chloride, Na, and urea levels, which were indicators of insufficient intake or GI loss (the details are shown at http://v.ai-care.top/A2). In November 1004, 53.9% of AI attention was assigned to serum albumin (the albumin level decreased from 38 g/L to 20.8 g/L). Finally, during the last visit in December, the risk score for this patient was 90.3, and 66.2% attention was given to the albumin level. AICare captured the most important clinical features related to patient death and generated a timely high risk score.

### Materials and ethics issues

This retrospective study was approved by the Medical Scientific Research Ethical Committee. The input of AICare is routinely collected laboratory test results and static baseline information. Patients do not need to conduct any additional unnecessary tests. Our study was granted an exemption from informed consent by the ethics committee due to its retrospective nature and the fact that it did not involve any intervention in the patients’ treatment.

Our paper and online system do not contain any sensitive information that could be used to identify individual patients. Patients’ private information was anonymized during the analysis. Patient names were replaced with unique patient IDs (e.g., A1, A2). Contact information, including phone numbers and addresses, was deleted. Clinical visit dates were reset to start in 1000 (year) in the online visualization system, and the date of birth was also reset with the corresponding offset.

We developed a practical AI-doctor interaction system to visualize the trajectory of patients’ health status and risk indicators. Our developed health trajectory visualization system with anonymous case studies (patient IDs A1–A20) is publicly available at http://v.ai-care.top/A8. Visualization of the importance of the features is available at http://v.ai-care.top/statistics/feature.

All code for the model, the deep learning training parameters, and the training data are openly available. They can be accessed on Zenodo via https://doi.org/10.5281/zenodo.10003570 and http://v.ai-care.top/download. Users can upload data online to get prediction results immediately (http://v.ai-care.top/predict) or download the code to train the model offline based on their dataset. More data that support the findings of this study are available from the corresponding author upon reasonable request.

## Discussion

### Implications

The whole procedure of PD treatment needs a dynamic prediction of patient mortality risk to help patients prevent adverse outcomes, based on the medical records collected along with the visits. Individual-level dynamic mortality prediction for long-term PD has not yet been substantially studied. Besides, deep models, which can capture complex longitudinal progressions, are often black boxes and fail to provide human-understandable interpretation. Thus, medical professionals lack trustworthiness in deep models.

In this work, we develop a deep learning-based generalizable model capable of learning massive amounts of EMR data and exploring personal characteristics to perform clinical predictions. AICare captures the clinical features that strongly indicate the health status of patients in various conditions. It builds personal health status embedding and provides reasonably fine-grained interpretability in terms of feature importance at each follow-up visit.

In the experiment based on a real-world clinical dataset, 656 incident PD patients were enrolled at the Department of Nephrology of a large grade A tertiary hospital. AICare is used to predict 1-year mortality at each follow-up visit. We compare the performance of AICare with existing related SOTA clinical predictive models. The experiment results show that AICare outperforms the published baseline approaches with 11.8% relative improvement on AUPRC and powerful interpretability.

To facilitate personalized clinical service and verify the reasonability of the model, we develop an AI-doctor interaction system to reveal the patient’s health trajectory and the corresponding vital biomarkers while predicting a prognosis. After the trial of our system, experienced nephrology department physicians suggest that AICare can offer opportunities to identify patients with potential mortality risks within a time window that enables early individualized treatment and outcome improvement. The medical knowledge learned by AICare has been positively confirmed by human medical experts and related medical literature.

### Key findings and clinical recommendations restatements

#### Important features summary

Some of the key findings generated by AICare are summarized below. For more details about the medical findings, please see Figures 3 and 4 and Table 5.

Albumin is the most indicative feature for the prediction of 1-year mortality in patients with PD, especially for GI disease, PVD, and living patients. The feature importance weight of albumin presents as a V-shaped curve along with the albumin level. A higher albumin level brings better survival expectations. AICare recommends raising the albumin level to above 32 g/L as much as possible for most PD patients.

DBP is the second important feature. It is indicative especially for cachexia, cancer, CVE and living patients. SBP is indicative for cancer and PD-associated peritonitis (PDAP). The importance weight of DBP and SBP presents as V-shaped and L-shaped curves, respectively. AICare recommends raising the DBP to above 70 mm Hg for most PD patients. AICare recommends maintaining the SBP at least 130 mm Hg. But further increases of SBP will not bring many benefits.

Cl is indicative for cachexia and infection patients. The importance weight of Cl presents as a V-shaped curve. A higher Cl level brings better survival expectations. AICare recommends raising the Cl level to above 96 mmol/L for most PD patients.

Cr is indicative for GI disease and cardiovascular disease (CVD) patients. AICare recommends raising the Cr level to above 900 μmol/L, which is a rough recommendation for most PD patients in this dataset. We will specify this finding for different cohorts (e.g., different gender) in future work.

Urea is indicative for PDAP and PVD patients. AICare recommends raising the urea level to above 20 mmol/L for most PD patients.

Phosphorus (P) is indicative for PDAP, cancer, CVD, and CVE patients. The importance weight of P presents as an L-shaped curve. AICare recommends raising the P level to above 1.5 mmol/L for most PD patients. Further increases will not bring benefits.

Hb is indicative for GI disease patients. The importance weight of Hb presents as an L-shaped curve. AICare recommends raising Hb level above 114 g/L for most PD patients. Further increases will not bring many benefits.

#### Prediction performance for mortality causes

As shown in Table.S1, experiment results indicate that AICare can effectively predict the most common adverse outcomes of PD patients (e.g., cachexia, PVD, infection, and cancer). However, CVE and CVD are the most challenging mortality causes to predict. CVE^47^ patients in particular often acutely suffer from sudden death without apparent signs. This suggests that more frequent clinical follow-up and more clinical tests should be included as features (e.g., ECG examination) to perform early screening for CVE.

### Limitations and future work

#### Introducing multicenter EMRs to increase the data amount

A major limitation of this study is the single-center design, which makes the data amount of the research scarce. The limitation also results in a relatively small sample of positive cases. However, the analyzed data of 656 PD patients with 13,091 visits cover a long-term longitudinal trajectory of PD patients. There are about 20 visits recorded for each patient, with an average visit interval of 2.7 months and an average follow-up time of 4 years. To the best of our knowledge, this is rarely seen in the existing medical literature. Besides, we also introduce an HD EMR dataset as an additional experimental dataset to test the prediction performance. In future work, we will extend AICare to multi-center healthcare systems and conduct a prospective multicenter controlled experiment to validate the framework in other clinical scenarios.

#### Incorporating more clinical features to depict health status

During the data collection process for this study, we collected many medical features of patients, most of which were discarded due to high missing rates. Our model only had access to the autofiltered 16 longitudinal medical features and 4 demographic features for each patient. The novelty of this research does not only lie in incremental model performance improvements. This predictive performance was achieved without hand selection or hand-made variables deemed important by a medical expert. AICare can achieve satisfactory prediction results and discover medical findings, proving the model’s validity and practicability. In future releases, we expect to incorporate more medical features, such as medication records, dialysis adequacy records, complication records, and health data collected at home.

#### Providing recommendations for diverse patient cohorts

To obtain relatively stable and reasonable conclusions, the clinical recommendations in this paper are roughly generated by AICare for most PD patients. As more data are collected in the next release, we will provide refined recommendations for diverse patient cohorts (e.g., different genders and ages).

#### Embedding sequences with a relatively regular time interval

Our 1-year mortality risk prediction system, as developed in this paper, is primarily designed for scenarios involving outpatient follow-up of PD patients. It aligns with the approach of most relevant methods that deal with sequential EMRs using time-series-encoding models for prognosis prediction tasks, as illustrated by the methods listed in the Related work section in the supplemental information. Our network framework, AICare, is best suited for situations where there is a relatively regular interval between medical visits. For example.

(1) The majority of PD patients typically undergo routine outpatient lab tests at hospitals approximately every 2.5 months.
(2) In intensive care unit (ICU) settings, medical records for patients might be recorded on a daily basis or at even shorter intervals, as demonstrated in the sepsis prediction task in the supplemental information.

However, we acknowledge that some scenarios may involve significantly irregular records. In cases where raw data are input into our model without regularization, it could potentially result in a decline in prediction performance. To address this issue, it is crucial to preprocess the data using regularization techniques^48^ or implement time-aware mechanisms to mitigate the adverse effects of irregular records.^49^^,^^50^ Dealing with irregular records is indeed an important research branch within EMR analysis, but it is not the primary focus of this study.

#### Features with low importance weight

No significantly meaningful importance pattern was discovered for white blood cell count (WBC). This may be because WBC is not a crucial feature in mortality prediction or because WBC is such a special clinical feature that AICare does not know how to use it to embed the health representation. This reminds us to design proper embedding network modules (e.g., convolutional layers) to effectively utilize different feature effectively. Besides, considering that the proportion of immune cells may indicate the health status as a human-constructed advanced feature, we will introduce more related clinical features about the immune.

#### A robust prospective evaluation in future work

In this study, our focus is on application of our methodology to the 1-year mortality risk prediction task for PD patients and the interpretation of the results. Although it is widely believed that accurate predictions can be used to improve care,^51^ this is not a foregone conclusion, and prospective trials are needed to demonstrate this.^52^^,^^53^ We acknowledge that clinical practice is influenced by numerous factors, and while our observations provide some new medical insights, they should be considered preliminary and require further validation in larger prospective evaluations to establish efficacy and safety for patients. These observations, while serving as a reference and source of inspiration for subsequent research on EMR analysis, should not be immediately implemented in clinical practice without undergoing further validation. We will recruit patients and conduct a larger and more robust prospective evaluation in the future. We will conduct a blinded application-grounded evaluation by inviting dozens of experienced medical practitioners (with 7–20 years of clinical practice time) from nephrology departments of different hospitals to test the practical effectiveness and degrees of physicians’ recognition. The prototype version of the trial system with online questionnaires has already been developed and can be found at http://v.ai-care.top/table/questionnaire/a1. The AI-doctor interaction system is available in English and simplified Chinese. The questionnaire page is currently only available in simplified Chinese.

### Conclusion

AICare is a generic framework proposed to model a patient’s health status based on multivariate time-series EMR data. The AICare captures complex longitudinal progressions in patients’ health conditions and provides dynamic predictions of mortality risk while also offering fine-grained interpretability to understand how the model arrives at specific predictions for individual patients. This interpretability aspect enables them to build trust in the model’s predictions and better comprehend the reasoning behind the recommendations.

Our work also includes the development of an AI-doctor interaction system that leverages the capabilities of AICare to support clinicians in prognosis prediction. The system provides an intuitive visualization of the patient’s health trajectory over time, allowing clinicians to track changes in key health indicators and identify trends in mortality risk predictions. Moreover, AICare offers personalized recommendations based on the model’s predictions, suggesting target values for essential health indicators to improve the patient’s survival outlook. These recommendations are instrumental in helping clinicians devise personalized treatment plans and monitoring strategies for their patients.

## Experimental procedures

### Resource availability

#### Lead contact

Yasha Wang is the lead contact of this study and can be reached via e-mail (wangyasha@pku.edu.cn).

#### Materials availability

This study did not generate new unique reagents.

#### Data and code availability

The code, training parameters, and training data are openly available on Zenodo (https://doi.org/10.5281/zenodo.10003570) and our website (http://v.ai-care.top/download). Users can instantly get predictions by uploading data to http://v.ai-care.top/predict or download the code for offline training with their dataset. Additional supporting data for this study can be requested from the corresponding author.

## Supplemental information

Document S1. Supplemental experimental procedures, Figures S1–S5, Tables S1–S5, and supplemental references

Document S2. Article plus supplemental information
