# Supplementary material for: Mortality prediction with adaptive feature importance recalibration for peritoneal dialysis patients
Source: Patterns (N Y). 2023 Dec 8;4(12):100892. doi: 10.1016/j.patter.2023.100892 (PMC10724364; doi:10.1016/j.patter.2023.100892)
Supplement: Document S1. Supplemental experimental procedures, Figures S1–S5, Tables S1–S5, and supplemental references [file mmc1.pdf]

Patterns, Volume *4*

Supplemental information

Mortality prediction with adaptive feature importance recalibration for peritoneal dialysis patients

Liantao Ma, Chaohe Zhang, Junyi Gao, Xianfeng Jiao, Zhihao Yu, Yinghao Zhu, Tianlong Wang, Xinyu Ma, Yasha Wang, Wen Tang, Xinju Zhao, Wenjie Ruan, and Tao Wang

Appendix for Mortality Prediction with Adaptive Feature Importance Recalibration for Peritoneal Dialysis Patients

Materials and Method Details

Dataset

This work includes 13,091 visits of 656 end-stage renal disease peritoneal dialysis patients from the Department of Nephrology of a large grade A tertiary (the highest level in the nation's three-tier grading system for hospitals). Fig. s1 shows the distribution of age and visit frequency. The average age of patients at the first clinical visit is 58.55 years old, with a standard deviation (Std) of 15.81 years. The average visiting frequency of patients at the end of the clinical follow-up was 19.95, with an Std of 13.53.

Problem Formulation

We formulate the model inputs and prediction tasks as follows:

Definition 1 (**Patient Records**)**.** A patient's visit records can be represented as a matrix $\mathbf{R} \in \mathbb{R}^{N \times T}$, where N denotes the number of medical features in visit records and T denotes the number of visits within the observation window. We use vector $\mathbf{r}_{n,t}$ to denote the t-th visit of the n-th feature. The baseline information is denoted as vector $\mathbf{r}_0$.

Problem 1 (**One-year Mortality Prediction**)**.** Given a patient's visit records $\mathbf{R}$ and baseline information $\mathbf{r}_0$, our objective is to predict the mortality risk $\widehat{\mathbf{y}}_t$ in the next year for the patient at each visit. This is formulated as a binary classification task as $\mathbf{y} \in \{0, 1\}$.

Considering the uncertainty of the health status variation in the observation window, we design a particular labeling strategy to make the training labels as close to the ground truth as possible. As shown in Fig. s2, for patients with positive labels (i.e., mortality) at the end of clinical follow-up, we consider all visits within one year before the mortality date as high-risk visits ($y = 1$). For patients with negative labels at the end, we consider all visits within one year before the last visit as *uncertain*, since we do not know whether the patient will have an adverse outcome in the next year ($y = uncertain$). The calculation of loss function and performance metrics will not include these visits. Other visits are all labeled as low-risk ($y = 0$).

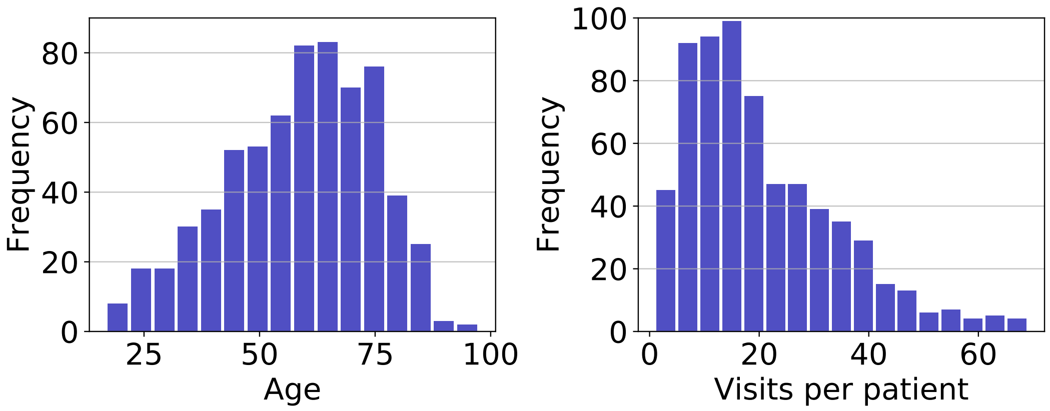

Figure s1: **Distribution of Age and Visit Frequency in PD Dataset.**

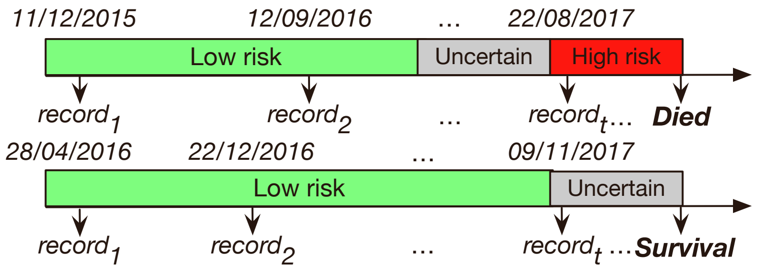

Figure s2: **Label Assignment.** The prediction task is defined as a 1-year mortality prediction at each clinical visit. Clinical visits within 1 year before death are labeled as *high risk* $(y = 1)$. Visits recorded 2 years before death are labeled as *low risk* $(y = 1)$. Visits recorded between 1 and 2 years before death are labeled as *uncertain* status and will not be included in the training process.

Problem 2 (**Model Interpretability**)**.** For a given patient, the model will output an importance score matrix α, where the value $\alpha_{n,t}$ in the n-th row and t-th column denotes the importance score for feature n at visit t. This importance score represents how much the feature contributes to the prediction.

Model Detail

We propose a general healthcare predictive model, which can adaptively depict patients' health status in diverse conditions and provide reasonable interpretability. The model explicitly captures the interdependencies among time series of dynamic features and static baseline information to learn the personal health context of patients in a global view. As shown in Fig.1-(2), `AICare` comprises the following sub-modules:

- The multi-channel feature extraction module is developed to learn the representation of each dynamic feature separately.
- The adaptive feature importance recalibration module enhances the key factors explicitly by squeeze-and-excitation block to perform the individualized clinical prediction.

Multi-Channel Feature Extraction

The patient's health status is depicted by dynamic feature sequences and static baseline features. To embed such heterogeneous information and build the final health representation, as well as make the dynamic feature's importance assignment process more intuitive, we develop a multi-channel feature extraction module.

Considering that the logical time order of the clinical sequence matters in the medical domain, `AICare` embeds the time series of each dynamic feature separately by multi-channel bidirectional GRUs. The GRUs at the bottom level will refine the representation of each feature and form a sequence of $N + 1$ feature vectors (N for dynamic record features $\mathbf{R}$ and 1 for the baseline information $\mathbf{r}_0$). Specifically, `AICare` embeds the time series of each feature separately by multi-channel bidirectional GRU:

$$(\overrightarrow{\mathbf{f}_{n,1}}, ..., \overrightarrow{\mathbf{f}_{n,T}}), (\overleftarrow{\mathbf{f}_{n,1}}, ..., \overleftarrow{\mathbf{f}_{n,T}}) = \text{bi-GRU}_n(\mathbf{r}_{n,1}, ..., \mathbf{r}_{n,T}), \quad (1)$$

where the time series of feature n is denoted as $\mathbf{r}_{n,:} = (\mathbf{r}_{n,1}, ..., \mathbf{r}_{n,T}) \in R^T$. We derive each feature representation as the sum of the two last hidden embeddings:

$$\mathbf{f}_n = \overrightarrow{\mathbf{f}_{n,T}} + \overleftarrow{\mathbf{f}_{n,1}}. \tag{2}$$

Furthermore, the demographic baseline data ($\mathbf{r}_0$) is embedded as:

$$\mathbf{f}_0 = W_0^{emb} \cdot \mathbf{r}_0, \tag{3}$$

where W_0^{emb} is the embedding matrix. From here on, we ignore the bias term for ease of notation. Thus, all the patient data can be represented by a matrix $\mathbf{F}$ (i.e., a sequence of vectors, where each vector represents one feature of the patient over time): $\mathbf{F} = (\mathbf{f}_0, \mathbf{f}_1, \cdots, \mathbf{f}_N)$.

Adaptive Feature Importance Recalibration

We develop an adaptive feature importance recalibration module to provide the feature's importance weight when performing mortality prediction at each clinical visit. This attention-based module is inspired by the $SEblock$ in the research area of computer vision[1], and is trained to explicitly model the dependencies between clinical features. It can selectively give more weight to the representative and predictive features but suppresses unimportant ones.

First, to select the most informative features, we should provide the model with a global view of patients' status at the current visit. For a particular patient, `AICare` squeezes all the feature embedding via a mean pooling operation to get an integrated but straightforward picture of the health status. It can be regarded as an abstract of the patient's all historical status. The importance of different temporal patterns will be calculated based on this abstract. Second, we selectively give more weight to the predictive features but suppress unimportant ones, which contribute little to the prediction target. The selectively enhanced predictive features can be treated as a precursor of health risk for the given patient.

Concretely, the $Query$ is obtained by $\mathbf{f}_{sqz}$ from embedded health information $\mathbf{F}$, including dynamic features and baseline features. The Keys are formed by embedded dynamic features $\mathbf{f}_1, \cdots, \mathbf{f}_N$ as:

$$\mathbf{f}_{sqz} = \text{Mean\_pooling}(\mathbf{f}_0, \mathbf{f}_1, \cdots, \mathbf{f}_N), \tag{4}$$

$$\mathbf{q}_{sqz}^{fin} = W_{sqz}^{fin} \cdot \mathbf{f}_{sqz}, \tag{5}$$

$$\mathbf{k}_n^{fin} = W_n^{fin} \cdot \mathbf{f}_n, \quad (n = 1, ..., N), \tag{6}$$

where W_{sqz}^{fin} and W_n^{fin} are the projection matrix, respectively. Then the attention weights are calculated as follows:

$$\alpha_1^{fin}, ..., \alpha_N^{fin} = \delta(\zeta_1^{fin}, ..., \zeta_N^{fin}), \tag{7}$$

$$\zeta_n^{fin} = \mathbf{q}_{sqz}^{fin} \cdot \mathbf{k}_n^{fin} \quad (n = 1, ..., N), \tag{8}$$

where δ denotes the activation function (i.e., *softmax* or *sparsemax*). *Sparsemax* activation will make the model interpretability more prominent by allowing the most critical features to dominate the final embedding. Using *sparsemax* activation will slightly weaken the performance since it suppresses too much information from features that are not most important. In this work, we employ *softmax* function to obtain the prediction results, and employ *sparsemax* function to extract medical findings. Finally, the health status representation s and the prediction result $\widehat{\mathbf{y}}$ can be obtained by:

$$\mathbf{s} = \text{Concat}[\sum_{n=1}^{N} \alpha_n^{fin} \cdot \mathbf{f}_n^*, \ \mathbf{f}_0^*], \tag{9}$$

$$\widehat{\mathbf{y}} = \mathrm{Sigmoid}(W^{final} \cdot \mathbf{s}), \tag{10}$$

where W^{final} is the weight matrix.

Experiment Details

Experiment Setup

Implementation Details

The training is done in a machine equipped with CPU: Intel Xeon E5-2630, 256GB RAM, and GPU: Nvidia Titan V using Pytorch 1.1.0. We use Adam[2] with the mini-batch of 256 patients, and the learning rate is set to $1e-3$. To fairly compare different approaches, the hyper-parameters of the baseline models are fine-tuned by a grid-searching strategy.

Baseline Approaches

We implement several state-of-the-art (SOTA) representative models as comparative baseline approaches. The hyper-parameters of models are fine-tuned by a grid-searching strategy.

GRU: Gated Recurrent Unit neural network embeds the time series to perform the target prediction. It is a widely applied variant of the Recurrent Neural Network (RNN), which improves the capability to maintain historical memories and reduces parameters in the update and reset gates. GRU has been used to predict several severe complications (mortality, renal failure with a need for renal replacement therapy, and postoperative bleeding leading to operative revision) in post-cardiosurgical care in real-time (Lancet Respiratory Medicine, 2018)[3].

Transformer$_e$ is the encoder of the Transformer[4], which comprises the positional encoding module and the self-attention module. Transformer has been used to perform the mortality risk analysis for liver transplant recipients[5].

MT-RHN: Multi-Task Deep Recurrent Highway Network, which embeds the historical data and current step with a deep residual embedding component, and employs a multi-task learning strategy to enhance the performance. MT-RHN has been used to perform the continuous risk prediction of future acute kidney injury deterioration occurring in the next 48 hours (Nature, 2019)[6].

LSTM: Long Short Term Memory network is a variant of the Recurrent Neural Network (RNN), capable of learning long-term dependencies. LSTM has been used to perform the 90-day all-cause mortality in the intensive care unit (ICU), based on the concatenated static features and dynamic features (Lancet Digital Health, 2020)[7].

biLSTM-FC: Bidirectional LSTM with Fully Connected layers. Bidirectional LSTM (biLSTM) is a sequence processing model that consists of two LSTMs: one taking the input in a forward direction and the other in a backward direction. Sung et al. have used biLSTM-FC to perform the clinical event prediction (death, sepsis, and acute kidney injury), where biLSTM and fully connected layers are employed to embed the dynamic features and static features correspondingly (JMIR, 2021)[8].

XGBoost: a recursive tree-based supervised machine learning classifier. XGBoost has been used to predict the mortality for COVID-19 infected patients (Nature Machine Intelligence, 2020)[9].

DT: Decision Tree, a non-parametric supervised learning algorithm with a hierarchical tree structure. DT has been used to perform mortality prediction for peritoneal dialysis patients (Nature Scientific Reports, 2020)[10].

LightGBM: a gradient boosting ensemble framework that uses tree-based learning algorithms. LightGBM has been used to perform the early prediction of circulatory failure in the intensive care unit (Nature Medicine, 2020)[11].

LR: Logistic Regression model predicts a dependent data variable by analyzing the relationship between one or more existing independent variables. LR has been used to predict the early risk of chronic kidney disease in patients with diabetes (Nature Medicine, 2019)[12].

Evaluation Metrics

We evaluate the models with a 10-fold cross-validation strategy and report the average performance, similar to[13]. We assess performance for the binary classification problem using the area under the receiver operating characteristic curve (AUROC) and the area under the precision-recall curve (AUPRC). AUPRC is the most informative and the primary evaluation metric when dealing with a highly imbalanced and skewed dataset[14,15] like the real-world EMR data.

Hyper-Parameter Settings

Specially, the hyper-parameter setting of the proposed `AICare` is as follows: We set the embedding dimension and hidden dimension as 16 / 16 / 32 for PD Patients / Hemodialysis ESRD / Challenge dataset, respectively. We use Adam optimization algorithm with the batch size of 256 and the learning rate is set to $1e-3$. We implement several state-of-the-art models as our baseline approaches. For the hyper-parameter settings of our baseline models, our principle is as follows: If the hyper-parameter setting is available in the original paper, we will use the recommended setting. Otherwise, the hyper-parameters of the baseline models are fine-tuned by the grid-searching strategy.

GRU/LSTM/biLSTM-FC/Transformer/MT-RHN The hidden units are set to 16 / 16 / 32 for PD Patients / Hemodialysis ESRD / Challenge dataset, respectively, and the dropout rate is 0.5. **LR**: the number of max iterations is set to 200. **XGBoost**: the max depth is set to 5, with 50 estimators and 0.1 learning rate. **DT**: the max depth is set to 5. **LightGBM**: the max depth is set to 5, with 50 estimators and 0.01 learning rate.

Prediction Performance of `AICare` for Different Causes of Death on PD Dataset

There are nine different CODs recorded: Cerebrovascular Disease (CVE), Cardiovascular Disease (CVD), Peritoneal Dialysis Associated Peritonitis (PDAP, Peritonitis), Peripheral Vascular Disease (PVD), Infections, Gastrointestinal Disease (GI Disease), Cachexia, Cancer and Other causes. We evaluate the performance for patients with different causes of death (COD). Since our experiment was conducted via 10-fold cross-validation, we employ the model trained on each fold's training set to the corresponding testset to perform the prediction.

The statistics of patients with different mortality causes are shown in Table s1. The receiver operating characteristic (ROC) curves for different COD patient subgroups are shown in Fig. s3. According to the prediction results, the risk of cachaxia (AUROC = 0.88), infection (AUROC = 0.82) and PVD (AUROC = 0.82) are easy to be identified. `AICare` provides accurate prediction results of these patients about a year before the outcome.

On the contrary, patients with CVE (AUROC = 0.55) and CVD (AUROC = 0.71) are the most difficult to predict by the model. These diseases often attack untimely and acutely without obvious signs[16], compared to cachexia, infections, and PVD. Patients with these health risk factors

Table s1: **Statistics of patients with different mortality causes.** This real-world dataset contains 656 peritoneal dialysis (PD) patients. 261 (39.8%) patients of them, unfortunately, died before the final follow-up. There are nine different causes of death (COD) recorded.

Causes of Death	# Patients (%)	AUROC
Cerebrovascular Disease (CVE)	74 (28.3%)	0.55
Cardiovascular Disease (CVD)	21 (8.0%)	0.71
Gastrointestinal Disease (GI Disease)	17 (6.5%)	0.73
Peritoneal Dialysis Associated Peritonitis (PDAP)	21 (8.0%)	0.74
Cancer	23 (8.8%)	0.76
Other	50 (19.1%)	0.80
Infection	33 (12.6%)	0.82
Peripheral Vascular Disease (PVD)	13 (4.9%)	0.82
Cachexia	9 (3.4%)	0.88
Mortality	261 (100.0%)	-

may have a higher threat and probability of sudden death in quite a short period of time, which is hard to take early warnings.

Additional Experiments on External Public Datasets

`AICare` is a generic framework proposed to model the patient's health status on multi-variate time series EMR data. The analysis of the peritoneal dialysis dataset in this paper serves as a proof of concept. To verify the generalizability of `AICare`, we train the model to perform the prognosis prediction tasks on external real-world public datasets (i.e., mortality prediction on the DOPPS hemodialysis ESRD dataset, sepsis prediction on the PhysioNet Challange Dataset).

Mortality Prediction on the DOPPS Hemodialysis ESRD Dataset

We perform the 1-year mortality prediction on the DOPPS hemodialysis ESRD dataset[17] [1]. The statistics of the dataset are listed in Table s2. The results in Table s4 indicate that `AICare` also achieves better performance than the baseline models. We notice that XGBoost achieves a higher AUPRC while a lower AUROC. This is because AUPRC is often more informative than AUROC when dealing with highly imbalanced datasets. In a scenario where one class significantly outnumbers the other, a random classifier could achieve an AUROC of 0.5, but the AUPRC might be higher or lower depending on the underlying distribution of the data and the way the model is handling the classes. So both XGBoost and GRU might be focusing on aspects that lead to higher precision and recall for the positive class, hence the higher AUPRC, while XGBoost fails to distinguish between classes in general, reflected in the AUROC score.

Sepsis Prediction on the PhysioNet Challenge Dataset

We perform the sepsis prediction on an open-source challenge dataset[18] [2]. The sepsis prediction data is from three geographically distinct U.S. hospital systems with three different electronic medical record systems. These data were collected over the past decade with approval from

[1] The DOPPS hemodialysis ESRD dataset is made available directly through an international platform `https://www.dopps.org/OurStudies/HemodialysisDOPPS.aspx`, and it can be obtained from DOPPS research community website:`https://www.dopps.org/PartnerwithUs.aspx`.

[2] The Physionet Challenge dataset is available at `https://physionet.org/content/challenge-2019/`

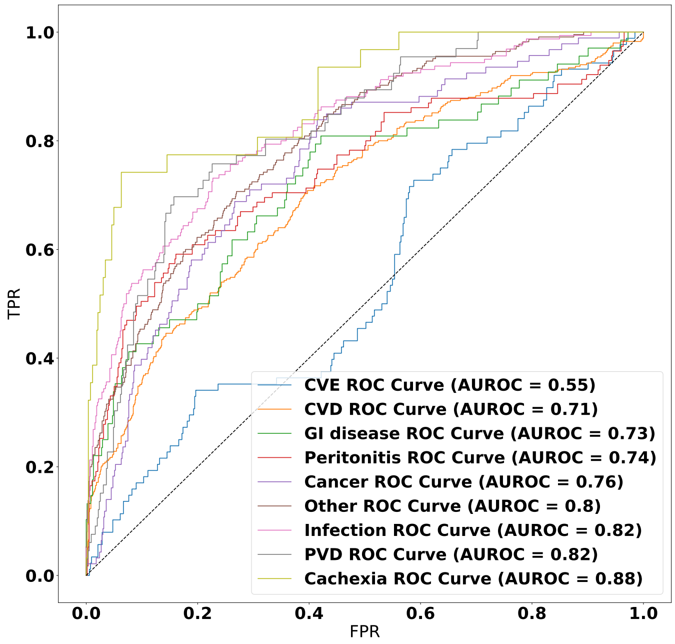

Figure s3: **Prediction ROC Results of Different Mortality Causes.** Cerebrovascular Disease (CVE) and Cardiovascular Disease (CVD) are the most challenging mortality causes to predict. On the contrary, Infections, Peripheral Vascular Disease (PVD), and Cachexia risks are relatively easy to be early identified.

Table s2: **Statistics of Hemodialysis Patient EMR as an Additional Dataset.** The real-world dataset contains 1,363 hemodialysis dialysis (HD) patients with 4,789 clinical visits. There are 12.55% patients, unfortunately, who died before the final follow-up. The average age of patients enrolled is 59 years old.

	Total	Survival (%)	Mortality (%)
# Patients	1363	1192 (87.45%)	171(12.55%)
# Visits	4789	4359 (91.02%)	430 (8.98%)
Avg. Age	59.38	57.89	68.67

Table s3: **Feature Summary of Hemodialysis (HD) Dataset.** This dataset comprises 18 dynamic features recorded at each clinical visit and 4 static baseline features recorded at the first visit.

Abbrev.	Full Name	Unit	Low Risk Visits ($y = 0$)			High Risk Visits ($y = 1$)			% Missing
Dynamic Features			Mean	Std	Median	Mean	Std	Median	
CO_2CP	CO_2 Combining Power	mmol/L	21.579	3.867	21.6	21.292	4.066	21.4	27%
WBC	White Blood Cell Count	x10^9/L	6.216	1.981	5.950	6.254	2.391	5.85	6%
Hb	Hemoglobin	g/L	107.9	17.283	109.5	103.6	18.66	105	6%
Ca	Calcium	mmol/L	2.272	0.24	2.258	2.253	0.236	2.24	41%
K	Potassium	mmol/L	5.045	0.818	5	4.851	0.851	4.8	13%
Na	Sodium	mmol/L	139.1	3.451	139	138.6	3.437	138.9	16%
Cr	Creatinine	umol/L	933.4	296.7	917.8	784.6	306.0	740.3	17%
P	Phosphorus	mmol/L	1.896	0.636	1.811	1.817	0.671	1.701	15%
Albumin	Albumin	g/L	39.49	4.212	39.9	36.94	4.838	37.5	27%
Glucose	Glucose	mmol/L	7.131	3.677	6.11	7.846	4.361	6.68	38%
pre-Weight	Pre-Dialysis Weight	kg	62.35	12.24	61.5	60.57	11.91	60.01	61%
pst-Weight	Post-Dialysis Weight	kg	59.94	11.96	59.06	58.23	11.58	57.23	62%
pre-SBP	Pre-Dialysis SBP	mmHg	147.3	20.58	147	146.7	20.96	148.6	61%
pst-SBP	Post-Dialysis SBP	mmHg	137.9	22.02	137.6	137.9	22.52	138.6	61%
pre-DBP	Pre-Dialysis DBP	mmHg	78.88	11.9	78.66	75.62	11.83	76	61%
pst-DBP	Post-Dialysis DBP	mmHg	77.44	12.05	77	73.62	12.53	73.33	61%
pre-Urea	Pre-Dialysis Urea	mmol/L	45.51	19.65	39.9	43.15	20.24	36.76	61%
pst-Urea	Post-Dialysis Urea	mmol/L	15.11	8.526	12.88	15.02	8.967	12.79	67%
Baseline Features									
BMI	Body Mass Index	-	21.87	3.633	21.39	21.83	4.081	21.60	57%
Gender	Female (0) or male (1)	-	0.54	0.498	1	0.557	0.497	1	0%
Age	Age	year	58.19	14.21	59	67.75	12.72	70	0%
Diabetes	Is (1) or not (0) has diabetes	-	0.267	0.442	0	0.351	0.478	0	0%

Table s4: **Mortality Prediction Performance on Hemodialysis ESRD Dataset.** Our proposed deep-learning-based model, `AICare`, outperforms other SOTA baseline comparative approaches.

Method	AUPRC	AUROC
GRU[3]	0.252(0.086)	0.702(0.083)
Transformer[5]	0.256(0.096)	0.695(0.100)
MT-RHN[6]	0.275 (0.089)	0.735 (0.080)
LSTM[7]	0.257 (0.085)	0.714 (0.074)
biLSTM-FC[8]	0.287 (0.082)	0.731 (0.078)
LR[12]	0.166 (0.076)	0.522 (0.031)
XGBoost[9]	0.222 (0.118)	0.518 (0.017)
DT[10]	0.202 (0.034)	0.539 (0.023)
LightGBM[11]	0.168 (0.111)	0.514 (0.020)
`AICare`	**0.325**** (0.122)	**0.743**** (0.088)

Table s5: **Results of the Sepsis Prediction on Challenge Dataset.**

Methods	AUPRC	AUROC
GRU[3]	0.7016 (0.026)	0.9352 (0.007)
Transformer[5]	0.6237 (0.031)	0.8946 (0.012)
MT-RHN[6]	0.2016 (0.019)	0.7378 (0.015)
LSTM[7]	0.7173 (0.025)	0.9351 (0.007)
biLSTM-FC[8]	0.7070 (0.026)	0.9303 (0.008)
LR[12]	0.0755 (0.004)	0.5040 (0.002)
XGBoost[9]	0.2986 (0.025)	0.8212 (0.012)
DT[10]	0.1924 (0.021)	0.6513 (0.016)
LightGBM[11]	0.2777 (0.025)	0.7972 (0.013)
`AICare`	**0.7731** (0.023)	.**0.9527** (0.019)

the appropriate Institutional Review Boards. They are labeled by Sepsis-3 clinical criteria. The cleaned dataset consists of 40,336 patients and consists of hourly vital sign summaries, lab values, and static patient descriptions. In particular, the data contained 40 clinical variables: 8 vital sign variables, 26 laboratory variables, and 6 demographic variables. We fix a test set of 10% of patients and divide the rest of the dataset into the training set and validation set with a proportion of 0.85 : 0.15. As shown in Table s5, on the PhysioNet dataset, `AICare` deals with 34-dimension lab test data and achieves relative improvements of 7.7% in AUPRC, compared to the best baseline models.

Additional Interpretability Analysis

Additional Case Study on AI-Doctor Interaction System

On the patient detail page, users can view the patient's static baseline demographic information, dynamic trajectories of biomarkers, and prediction results. The system automatically displays the most *key* biomarkers that dominate the prediction results and provides the importance weights assigned by the model.

Case III: Patient Died of Sudden Death and Insufficient Dialysis (Fig. s4)

This case was diagnosed with diabetic nephropathy and initiated PD therapy. Fig. s4 shows the details of the previous visits and the results of the mortality risk assessment for this patient.

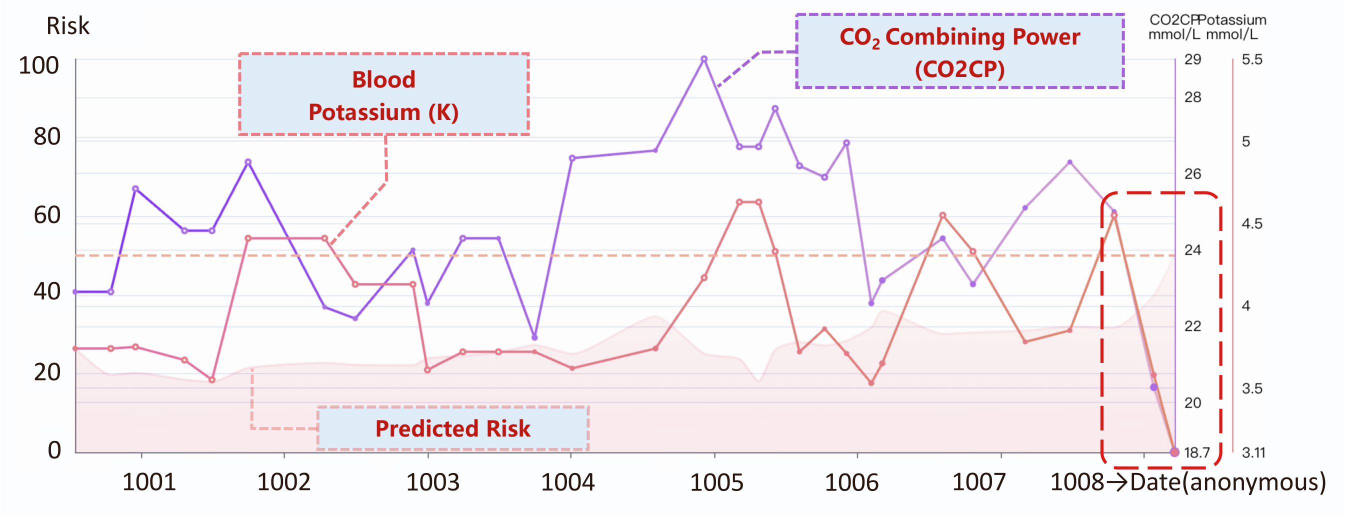

Figure s4: **Case Study III: Patient Died of Insufficient Dialysis and Sudden Heart Attack.** `AICare` pays most attention to CO2CP and Potassium for this patient. The health trajectory interactive visualization system is publicly deployed at `http://v.ai-care.top/A1`.

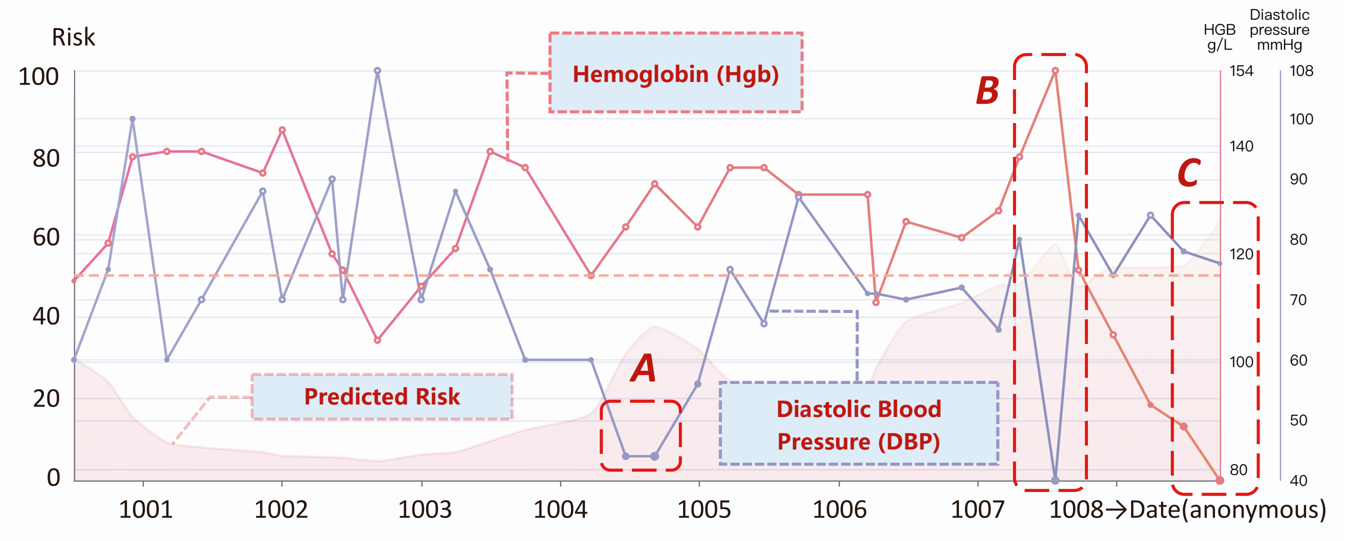

Figure s5: **Case Study IV: Patient Died of Gastrointestinal Bleeding.** `AICare` pays most attention to Diastolic Blood Pressure and Hemoglobin for this patient. The health trajectory interactive visualization system is publicly deployed at `http://v.ai-care.top/A3`.

The model believed that the patient's risk of death rose during the last two visits, indicating a deterioration of her health. The model assigned high attention weights to two medical features: carbon dioxide combining power (CO2CP) and potassium (K), due to the substantial decline of these two features at the last visits. AICare recommended focusing on the changes of carbon dioxide combining power (CO2CP) and potassium (K), where CO2CP decreased by 6.3 mmol/L rapidly (from 25.0 mmol/L to 18.7 mmol/L, 25.2% relative decrease), and K decreased by 1.44 mmol/L (from 4.55 mmol/L to 3.11 mmol/L, 31.6% relative decrease). The predicted mortality risk rapidly increased from 31.6 to 50.4 from September 1007 to March 1008.

This patient died on May, 1008. The cause of death was inadequate dialysis due to abdominal leakage of dialysate and sudden heart attack. The model sensed changes in important physiological indicators of the patient and provided early warning of possible risk factors for the patient. CO2CP was an indicator of acidosis, closely related to inadequate dialysis, and acidosis posed a health risk to the patient. The alert of potassium level could help physicians to give attention to it and treat hypokalemia by potassium supplement or hyperkalemia by necessary medical intervention.

Case IV: Patient Died of Gastrointestinal Bleeding (Fig. s5)

This case was diagnosed with glomerulonephritis and initiated PD therapy. There were three risk scores peaks during the whole PD treatment period. The first risk score peak (A) occurred on June, 1004. The risk score increased from 12.1 one year before, on July, 1003, to 37.53 on September, 1004. During this period, AI paid attention mainly to serum albumin (Please visit the link http://v.ai-care.top/A3 for details.). On September, 1004, AICare found abnormal value changes in blood pressure (DBP dropped from 60 mmHg to 44 mmHg and SBP dropped from 100 mmHg to 96 mmHg) and paid much attention to DBP (26.3%) and SBP (12%) behind serum albumin (33.8%). The second risk score peak (B) was on July, 1007. The risk score increased to 57.59. The model found abnormal values of SBP (73mm Hg) and DBP (40 mm Hg). Attention was mainly given to SBP (28.1%) and DBP (24.3%). The third risk peak (C) was on September, 1008. The patient risk score increased to 63.37. The model found an abnormal decline and low hemoglobin value (from 92g/L to 78g/L). The highest attention (26.1%) was given to hemoglobin level, followed by 25.5% of attention to serum albumin (29.1g/L). The patient died of gastrointestinal bleeding on November, 1008. AICare accurately captured the abnormality of blood pressure level and hemoglobin. Hemoglobin level was one of the main manifestations of gastrointestinal bleeding causing this patient's death.

Additional Observation of Feature Importance Variation

1)-5) For analysis of albumin, diastolic blood pressure, systolic blood pressure, creatinine, and hemoglobin, please see the main text.

6) Chlorine (Cl) Fig.3-(d). As shown in Table 3, the importance weight of chlorine presents as a V-shaped curve with 96 mmol/L as the lowest turning point. In the 82-96 mmol/L range, AICare pays higher attention weight to chlorine level as the chlorine gets lower and predicts a poor prognosis, marked as red dots in Fig.3-(d). Meanwhile, in the range of 96-120 mmol/L, the model pays more attention to chlorine level as it gets higher and predicts a good prognosis. AICare recommends raising the chloride level to above and even higher than 96 mmol/L for most PD patients. To the best of our knowledge, the direct effect of chlorine on PD patient mortality has not been investigated by previous studies. Some studies find that blood chlorine level positively correlates with residual renal function[19]. The suggested level of blood chlorine by AICare is highly consistent with the traditional reference range of chlorine for outpatients, which is 96-106 mmol/L.

7) Urea Fig.3-(f). The importance variation learned by AICare presents an L-shaped curve

with a 20 mmol/L turning point. The model pays more attention to urea levels in the range of 4-20 mmol/L as the urea level decreases. When urea level drops below 10 mmol/L, the model provides more than 20% of attention weights, and patients in this range are more likely to suffer a high mortality risk (i.e., $\hat{y} > 0.5$, marked as red dots in Fig.3-(f)). However, it nearly occupies no attention for urea levels in the range of 20-44 mmol/L. AICare recommends maintaining urea level at 20 mmol/L or slightly higher for most PD patients. It will not bring much benefit if urea rises above 20 mmol/L. This may be because the urea level indicates the nutritional status of PD patients. The traditional recommended reference range of urea for outpatient clinics is 3.1-9 mmol/L, which is inconsistent with AICare's suggestions. The traditional recommended range is only suitable for normal outpatients without ESRD. To the best of our knowledge, the recommended reference range of urea for PD patients has not been analyzed by existing works based on end-to-end deep learning methods.

8) Calcium (Ca) Fig.3-(g). The importance variation of calcium presents as an L-shaped curve with a turning point of 2.5 mmol/L. For calcium in the 1-2.5 mmol/L range, AICare pays more attention to calcium and tends to make adverse predictions as the calcium level decreases. For calcium over 2.5 mmol/L, the model pays nearly no attention to it. AICare recommends maintaining calcium level at least 2.5 mmol/L, while increasing calcium above 2.5 mmol/L may not bring much more benefits. The traditional recommended calcium reference range for outpatient clinics is 2.25-2.75 mmol/L, which is consistent with AICare's suggestions.

9) Sodium (Na) Fig.3-(h). The importance variation of sodium presents as an L-shaped curve with a turning point of 135.5 mmol/L. For sodium level in the 121-135.5 mmol/L, AICare pays more attention to sodium and tends to make adverse predictions as the sodium level decreases. This is consistent with the previous study that an increased mortality rate associated with hyponatremia in PD patients[20]. Moreover, a study in time-dependent analysis shows that, in PD patients, lower time-dependent and baseline sodium levels were independently associated with higher death risk[21]. Yet, for sodium level over 135.5 mmol/L, the model pays nearly no attention to it. Thus, AICare recommends maintaining sodium level at least 135.5 mmol/L, while increasing sodium above 135.5 mmol/L may not bring much more benefits. The traditional recommended reference range of sodium for outpatient clinics is 135-145 mmol/L, which is highly consistent with AICare's suggestions.

10) Potassium (K) Fig.4-(i). AICare pays more attention and tends to make adverse predictions as the potassium level decreases for potassium level in the range of 2.3-4 mmol/L. For potassium level over 4 mmol/L, the model pays nearly no attention to it. AICare recommends maintaining potassium level at 4 least mmol/L, but further improvement may not bring benefits. The traditional recommended reference range of potassium for outpatient clinics is 3.5-5.5 mmol/L. Previous studies have also reported the association between low potassium level and PD patients' poor outcomes[22,23].

11) Phosphorus (P) Fig.4-(j). AICare pays more attention and makes adverse predictions as the phosphate level decreases for phosphate level in the range of 0.5-1.5 mmol/L. AI recommends maintaining phosphate level at least 1.5 mmol/L, while further increase may not bring benefits. The traditional recommended reference range of phosphate for outpatient clinics is 1.1-1.3 mmol/L, which is **inconsistent** with AICare's suggestions. However, this is **consistent** with the KDOQI guidelines for preventing hyperphosphatemia in ESRD patients, which points out that the normal range of phosphate is 0.85-1.51 mmol/L. Low serum phosphorus is usually associated with poor dietary intake, and the association between low serum phosphorus and poor clinical outcome has been reported previously[24].

12) Carbon Dioxide Combining Power (CO2CP) Fig.4-(k). AICare pays more attention and makes adverse predictions as the CO2CP level decreases for CO2CP level in the range of 10.5-25 mmol/L. Yet, for CO2CP level over 25 mmol/L, the model pays nearly no attention to it. Thus, AICare recommends maintaining the CO2CP level at least 25 mmol/L. However, further

improvement on CO2CP does not help reduce mortality risk. The traditional recommended reference range of CO2CP for outpatient clinics is 20-29 mmol/L. According to previous studies, low serum bicarbonate indicated acidosis in PD patients, which is associated with catabolism, malnutrition, and poor outcome[25,26].

13) Body Weight Fig.4-(m). `AICare` pays more attention and makes adverse predictions as the body weight decreases for body weight in the range of 29-59 kg. For body weight over 59 kg, the model pays nearly no attention to it. `AICare` recommends maintaining body weight at 60 kg for most PD patients. However, the further improvement of body weight may not bring benefits. Malnutrition and low body weight are associated with higher mortality in peritoneal dialysis (PD)[27,28].

14) Glucose Fig.4-(n). `AICare` pays more attention and makes good predictions as the glucose level decreases for glucose level in the range of 1-6 mmol/L. For glucose level over 6 mmol/L, the model pays nearly no attention to it. Thus, AI recommends maintaining glucose level not exceeding 6 mmol/L. The traditional recommended reference range of glucose for outpatient clinics is 3.9-6.1 mmol/L, which is consistent with `AICare`'s suggestions.

15) Hypersensitive C-Reactive Protein (Hs-CRP) Fig.4-(o). Hs-CRP is a highly skewed L-shaped curve with a 16 mg/L turning point. For hs-CRP level 0-16 mg/L, `AICare` pays more attention and makes good predictions as the hs-CRP level decreases. For hs-CRP level over 16 mg/L, the model pays nearly no attention to it. Thus, `AICare` recommends maintaining the hs-CRP level not exceeding 16 mg/L. The traditional recommended reference range of hs-CRP for outpatient clinics is 0.5-10 mg/L, which is almost consistent with `AICare`'s suggestions.

16) White Blood Cell Count (WBC) Fig.4-(p). WBC is considered by `AICare` as the most eccentric clinical feature included in the dataset. For most patients, the importance weights assigned to WBC is below 1%. The model believes that WBC is not a crucial feature for ESRD patients when conducting 1-year mortality prediction.

As a preliminary conclusion drawn from the analysis of a specific peritoneal dialysis patient dataset based on deep learning methods, we suggest that readers use the above suggestions as inspiration for future work and clinical considerations, but do not directly implement specific recommendations for such indicators to clinical patients.

Related Work

Over the past ten years, there has been a massive explosion in the amount of digital information stored in electronic medical records, which opens a door for researchers to make secondary use of these records for various clinical applications[29–34]. At the same time, with the development of artificial intelligence, machine learning and deep learning-based models have shown the capability to perform renal-disease-related clinical predictions, including acute kidney injury risk prediction[6,8,35,36], graft loss prediction[37] and mortality prediction[8,10,37–42]. For instance, Bai et al.[43] proposed a study to assess the feasibility of machine learning (ML) in predicting the risk of end-stage kidney disease (ESKD) for patients with CKD.[44] developed a deep-learning-based prediction model for end-stage kidney disease (ESKD) in patients with primary immunoglobulin A nephropathy (IgAN). Noh et al.[10] conducted the 5-year mortality risk prediction task for peritoneal dialysis patients using the decision tree model. Xu et al.[35], Ravizza et al.[12] and Chaudhuri et al.[45] utilized patient static information to conduct the prediction for the progression of renal diseases, such as AKI, CKD and hospitalization. Besides, Akbilgic et al.[38], Liu et al.[39], Zhou et al.[40], Radovic et al.[41] and Kang et al.[42] used different ML-based methods to predict the mortality risk of patients with kidney-related diseases.

However, there are still some critical issues that have not yet been thoroughly addressed by existing work in terms of the following three issues.

I_1: **Perform dynamic mortality prediction at each follow-up visit based on the effective utilization of both sequential medical records and the baseline demographic information.** Most above-mentioned existing renal disease-related works only utilize static records. Such models cannot learn non-linear progression patterns from high-dimensional longitudinal EMR datasets, capturing the health status variation trajectory, limiting their prediction performance and applications in quality improvement initiatives or data-driven clinical decision-making processes. It requires significant efforts from clinicians, healthcare institutions and model developers to collect data and build longitudinal models for such long-term predictive tasks.

Some works attempted to model the dynamic information of patients. For example, Makino et al.[46] constructed a predictive model for diabetic kidney diseases (DKD) using AI, processing natural language and longitudinal data of diabetes patients. Rank et al.[36] developed a deep-learning-based real-time algorithm to predict postoperative AKI prior to the onset of symptoms and complications.Tomavsev et al.[6] and Hyland et al.[11] proposed DL-based models to conduct dynamic monitoring for the risk of AKI and circulatory failure on in-hospital patients with kidney disease. These work dynamically predict patients' risk through different time-series models based on deep learning and realize the dynamic monitoring of the health status of patients with kidney disease. However, the baseline data of patients with renal disease is also essential in diagnosing and treating. These researches have limitations in combining the static baseline information and dynamic data of patients, thence have difficulties comprehensively integrating the patient information for evaluation.

Thence, Srinivas et al.[37] combined the static and dynamic data of patients who received a kidney transplant and conducted the graft loss and mortality prediction via LR. Sung et al.[8] have used biLSTM-FC to perform the clinical event prediction (death, sepsis, and acute kidney injury), where biLSTM and fully connected layers are employed to embed the dynamic features and static features correspondingly. However, these works briefly concatenate the static and sequential information by the hidden units. The static information does not guide the individual health status representation learning or help the model adaptively assign weights to the features.

I_2: **Provide fine-grained interpretability for each patient individually by selecting key features which contribute the most to the mortality prediction (patient-level interpretability) and achieve high prediction performance simultaneously.** Although deep learning has achieved huge success in many domains, lack-of-explainability remains one serious drawback for the neural network. An interpretable model is essential for clinical decision support applications as the predictive results need to be understood by clinicians to adopt individualized treatment and extract medical knowledge. However, the model interpretability has not been fully discussed in most renal disease-related works. The decision-making process in such deep models is a black box and fails to provide human-understandable interpretability.

Several researchers have explored the interpretability in the medical feature via tree-based strategy. For example, Noh et al.[10] assessed mortality risk prediction in PD patients using decision tree algorithms. Akbilgic et al.[38] implemented a random forest method to predict outcomes of ESRD patients after dialysis initiation. However, the prediction performance of these static information-based methods is limited due to the deficiency of effective advanced clinical feature extraction.

Some recent works apply the SHapley Additive exPlanations (SHAP)[7,11], feature permutation[40,41], and inverse analysis[46] strategies to provide the post-hoc interpretability. For example, Makino et al. generated the time-series data pattern by inverse analysis. However, these interpreting methods usually only provide coarse-grained analysis and may face a difficult trade-off between the network complexity and prediction performance. As a result, it is still challenging to provide satisfying interpretability and achieve high prediction performance simultaneously.

I_3: **Adaptively analyze the importance of each feature along with the variation of its value (feature-level interpretability) to provide medical advice and extract knowledge.** The

interpretability shown in most of the existing EMR analysis works mainly focuses on tree-based interpretability and some forms of post-hoc interpretability. The tree-based analysis can only provide a fixed decision process for all patients and face a deficiency in sequential information utilization. To the best of our knowledge, none of the existing PD/HD/ESRD-related works explicitly provide the adaptive feature importance, analyze the changes of feature importance with its values, and extract medical advice based on ante-hoc interpretability in a deep end-to-end model.

References

1. Hu, J., Shen, L., and Sun, G. Squeeze-and-excitation networks. In: *Proceedings of the IEEE Conference on Computer Vision and Pattern Recognition* (2018):(7132–7141).

2. Kingma, D. P., and Ba, J. (2014). Adam: A method for stochastic optimization. arXiv preprint arXiv:1412.6980.

3. Meyer, A., Zverinski, D., Pfahringer, B., Kempfert, J., Kuehne, T., Sündermann, S. H., Stamm, C., Hofmann, T., Falk, V., and Eickhoff, C. (2018). Machine learning for real-time prediction of complications in critical care: a retrospective study. The Lancet Respiratory Medicine *6*, 905–914.

4. Vaswani, A., Shazeer, N., Parmar, N., Uszkoreit, J., Jones, L., Gomez, A. N., Kaiser, Ł., and Polosukhin, I. Attention is all you need. In: *Advances in neural information processing systems* (2017):(5998–6008).

5. Nitski, O., Azhie, A., Qazi-Arisar, F. A., Wang, X., Ma, S., Lilly, L., Watt, K. D., Levitsky, J., Asrani, S. K., Lee, D. S. et al. (2021). Long-term mortality risk stratification of liver transplant recipients: real-time application of deep learning algorithms on longitudinal data. The Lancet Digital Health *3*, e295–e305.

6. Tomašev, N., Glorot, X., Rae, J. W., Zielinski, M., Askham, H., Saraiva, A., Mottram, A., Meyer, C., Ravuri, S., Protsyuk, I. et al. (2019). A clinically applicable approach to continuous prediction of future acute kidney injury. Nature *572*, 116–119.

7. Thorsen-Meyer, H.-C., Nielsen, A. B., Nielsen, A. P., Kaas-Hansen, B. S., Toft, P., Schierbeck, J., Strøm, T., Chmura, P. J., Heimann, M., Dybdahl, L. et al. (2020). Dynamic and explainable machine learning prediction of mortality in patients in the intensive care unit: a retrospective study of high-frequency data in electronic patient records. The Lancet Digital Health *2*, e179–e191.

8. Sung, M., Hahn, S., Han, C. H., Lee, J. M., Lee, J., Yoo, J., Heo, J., Kim, Y. S., Chung, K. S. et al. (2021). Event prediction model considering time and input error using electronic medical records in the intensive care unit: Retrospective study. JMIR medical informatics *9*, e26426.

9. Yan, L., Zhang, H.-T., Goncalves, J., Xiao, Y., Wang, M., Guo, Y., Sun, C., Tang, X., Jing, L., Zhang, M. et al. (2020). An interpretable mortality prediction model for covid-19 patients. Nature Machine Intelligence (1–6).

10. Noh, J., Yoo, K. D., Bae, W., Lee, J. S., Kim, K., Cho, J.-H., Lee, H., Kim, D. K., Lim, C. S., Kang, S.-W. et al. (2020). Prediction of the mortality risk in peritoneal dialysis patients using

machine learning models: a nation-wide prospective cohort in korea. Scientific reports *10*, 1–11.

11. Hyland, S. L., Faltys, M., Hüser, M., Lyu, X., Gumbsch, T., Esteban, C., Bock, C., Horn, M., Moor, M., Rieck, B. et al. (2020). Early prediction of circulatory failure in the intensive care unit using machine learning. Nature medicine *26*, 364–373.

12. Ravizza, S., Huschto, T., Adamov, A., Böhm, L., Büsser, A., Flöther, F. F., Hinzmann, R., König, H., McAhren, S. M., Robertson, D. H. et al. (2019). Predicting the early risk of chronic kidney disease in patients with diabetes using real-world data. Nature medicine *25*, 57–59.

13. Ma, T., Xiao, C., and Wang, F. Health-atm: A deep architecture for multifaceted patient health record representation and risk prediction. In: *Proceedings of the 2018 SIAM International Conference on Data Mining*. SIAM (2018):(261–269).

14. Davis, J., and Goadrich, M. The relationship between precision-recall and roc curves. In: *Proceedings of the 23rd international conference on Machine learning*. ACM (2006):(233–240).

15. Choi, E., Xiao, C., Stewart, W., and Sun, J. Mime: Multilevel medical embedding of electronic health records for predictive healthcare. In: *Advances in Neural Information Processing Systems* (2018):(4547–4557).

16. Wikipedia contributors. Cerebrovascular disease — Wikipedia, the free encyclopedia (2019). URL: `https://en.wikipedia.org/w/index.php?title=Cerebrovascular_disease&oldid=876465095` [Online; accessed 3-February-2019].

17. Zhao, X., Niu, Q., Gan, L., Hou, F. F., Liang, X., Ni, Z., Chen, Y., Zhao, J., Bieber, B., Robinson, B. et al. (2021). Baseline data report of the china dialysis outcomes and practice patterns study (dopps). Scientific reports *11*, 873.

18. Reyna, M. A., Josef, C. S., Jeter, R., Shashikumar, S. P., Westover, M. B., Nemati, S., Clifford, G. D., and Sharma, A. (2019). Early prediction of sepsis from clinical data: the physionet/computing in cardiology challenge 2019. Critical Care Medicine.

19. Li, L., Liang, W., Ye, T., Chen, Z., Zuo, X., Du, X., Qian, K., Zhang, C., Hu, X., Li, J. et al. (2016). The association between nutritional markers and biochemical parameters and residual renal function in peritoneal dialysis patients. PLoS One *11*, e0156423.

20. Al-Chidadi, A., Nitsch, D., and Davenport, A. (2017). The effect of serum sodium on survival in patients treated by peritoneal dialysis in the united kingdom. Peritoneal dialysis international *37*, 70–77.

21. Ravel, V. A., Streja, E., Mehrotra, R., Sim, J. J., Harley, K., Ayus, J. C., Amin, A. N., Brunelli, S. M., Kovesdy, C. P., Kalantar-Zadeh, K. et al. (2017). Serum sodium and mortality in a national peritoneal dialysis cohort. Nephrology Dialysis Transplantation *32*, 1224–1233.

22. Davies, S. J., Zhao, J., Morgenstern, H., Zee, J., Bieber, B., Fuller, D. S., Sloand, J. A., Vychytil, A., Kawanishi, H., Johnson, D. W. et al. (2021). Low serum potassium levels and clinical outcomes in peritoneal dialysis—international results from pdopps. Kidney international reports *6*, 313–324.

23. Szeto, C.-C., Chow, K.-M., Kwan, B. C.-H., Leung, C.-B., Chung, K.-Y., Law, M.-C., and Li, P. K.-T. (2005). Hypokalemia in chinese peritoneal dialysis patients: prevalence and prognostic implication. American journal of kidney diseases *46*, 128–135.

24. Liu, C.-T., Lin, Y.-C., Lin, Y.-C., Kao, C.-C., Chen, H.-H., Hsu, C.-C., and Wu, M.-S. (2017). Roles of serum calcium, phosphorus, pth and alp on mortality in peritoneal dialysis patients: a nationwide, population-based longitudinal study using twrds 2005–2012. Scientific reports *7*, 1–9.

25. Szeto, C., and Lal, K. (1998). Metabolic acidosis and nutritional status of patients receiving continuous ambulatory peritoneal dialysis (capd). The International journal of artificial organs *21*, 192–195.

26. Kang, D.-H. (1999). Metabolic acidosis as a catabolic factor in peritoneal dialysis patients. Peritoneal dialysis international *19*, 304–308.

27. Imam, T. H., Shi, J. M., Yi, D. K., and Yang, S.-J. (2021). Long-term peritoneal dialysis is associated with a decrease in body weight. Clinical kidney journal.

28. Taylor, P., Keshaviah, R., and Beecroft, M. L. (1996). Adequacy of dialysis and nutrition in continuous peritoneal dialysis: Association with clinical outcomes1. J, Am. Soc. Nephrol *7*, 198–207.

29. Ma, L., Gao, J., Wang, Y., Zhang, C., Wang, J., Ruan, W., Tang, W., Gao, X., and Ma, X. Adacare: Explainable clinical health status representation learning via scale-adaptive feature extraction and recalibration. In: *Thirty-Fourth AAAI Conference on Artificial Intelligence* (2020):.

30. Ma, L., Zhang, C., Wang, Y., Ruan, W., Wang, J., Tang, W., Ma, X., Gao, X., and Gao, J. Concare: Personalized clinical feature embedding via capturing the healthcare context. In: *Thirty-Fourth AAAI Conference on Artificial Intelligence* (2020):.

31. Gao, J., Xiao, C., Wang, Y., Tang, W., Glass, L. M., and Sun, J. Stagenet: Stage-aware neural networks for health risk prediction. In: *Proceedings of The Web Conference 2020* (2020):(530–540).

32. Gao, J., Xiao, C., Glass, L. M., and Sun, J. (2020). Dr. agent: Clinical predictive model via mimicked second opinions. Journal of the American Medical Informatics Association *27*, 1084–1091.

33. Gao, J., Yang, C., Heintz, J., Barrows, S., Albers, E., Stapel, M., Warfield, S., Cross, A., and Sun, J. (2022). Medml: fusing medical knowledge and machine learning models for early pediatric covid-19 hospitalization and severity prediction. Iscience *25*.

34. Ma, L., Ma, X., Gao, J., Jiao, X., Yu, Z., Zhang, C., Ruan, W., Wang, Y., Tang, W., and Wang, J. Distilling knowledge from publicly available online emr data to emerging epidemic for prognosis. In: *Proceedings of the Web Conference 2021* (2021):(3558–3568).

35. Xu, Z., Luo, Y., Adekkanattu, P., Ancker, J. S., Jiang, G., Kiefer, R. C., Pacheco, J. A., Rasmussen, L. V., Pathak, J., and Wang, F. Stratified mortality prediction of patients with acute kidney injury in critical care. In: *MEDINFO 2019: Health and Wellbeing e-Networks for All* (462–466). IOS Press (2019):(462–466).

36. Rank, N., Pfahringer, B., Kempfert, J., Stamm, C., Kühne, T., Schoenrath, F., Falk, V., Eickhoff, C., and Meyer, A. (2020). Deep-learning-based real-time prediction of acute kidney injury outperforms human predictive performance. NPJ digital medicine *3*, 1–12.

37. Srinivas, T., Taber, D., Su, Z., Zhang, J., Mour, G., Northrup, D., Tripathi, A., Marsden, J., Moran, W., and Mauldin, P. (2017). Big data, predictive analytics, and quality improvement in kidney transplantation: a proof of concept. American Journal of Transplantation *17*, 671–681.

38. Akbilgic, O., Obi, Y., Potukuchi, P. K., Karabayir, I., Nguyen, D. V., Soohoo, M., Streja, E., Molnar, M. Z., Rhee, C. M., Kalantar-Zadeh, K. et al. (2019). Machine learning to identify dialysis patients at high death risk. Kidney international reports *4*, 1219–1229.

39. Liu, J., Wu, J., Liu, S., Li, M., Hu, K., and Li, K. (2021). Predicting mortality of patients with acute kidney injury in the icu using xgboost model. Plos one *16*, e0246306.

40. Zhou, Q., You, X., Dong, H., Lin, Z., Shi, Y., Su, Z., Shao, R., Chen, C., and Zhang, J. (2021). Prediction of premature all-cause mortality in patients receiving peritoneal dialysis using modified artificial neural networks. Aging (Albany NY) *13*, 14170.

41. Radović, N., Prelević, V., Erceg, M., and Antunović, T. (2022). Machine learning approach in mortality rate prediction for hemodialysis patients. Computer Methods in Biomechanics and Biomedical Engineering *25*, 111–122.

42. Kang, M. W., Kim, J., Kim, D. K., Oh, K.-H., Joo, K. W., Kim, Y. S., and Han, S. S. (2020). Machine learning algorithm to predict mortality in patients undergoing continuous renal replacement therapy. Critical Care *24*, 1–9.

43. Bai, Q., Su, C., Tang, W., and Li, Y. (2022). Machine learning to predict end stage kidney disease in chronic kidney disease. Scientific reports *12*, 1–8.

44. Schena, F. P., Anelli, V. W., Trotta, J., Di Noia, T., Manno, C., Tripepi, G., D'Arrigo, G., Chesnaye, N. C., Russo, M. L., Stangou, M. et al. (2021). Development and testing of an artificial intelligence tool for predicting end-stage kidney disease in patients with immunoglobulin a nephropathy. Kidney International *99*, 1179–1188.

45. Chaudhuri, S., Han, H., Usvyat, L., Jiao, Y., Sweet, D., Vinson, A., Steinberg, S. J., Maddux, D., Belmonte, K., Brzozowski, J. et al. (2021). Machine learning directed interventions associate with decreased hospitalization rates in hemodialysis patients. International Journal of Medical Informatics *153*, 104541.

46. Makino, M., Yoshimoto, R., Ono, M., Itoko, T., Katsuki, T., Koseki, A., Kudo, M., Haida, K., Kuroda, J., Yanagiya, R. et al. (2019). Artificial intelligence predicts the progression of diabetic kidney disease using big data machine learning. Scientific reports *9*, 1–9.
