# Supplementary material for: Mortality prediction with adaptive feature importance recalibration for peritoneal dialysis patients
Source: Patterns (N Y). 2023 Dec 8;4(12):100892. doi: 10.1016/j.patter.2023.100892 (PMC10724364; doi:10.1016/j.patter.2023.100892)
Supplement: Document S2. Article plus supplemental information [file mmc2.pdf]

Patterns

Article

Mortality prediction with adaptive feature importance recalibration for peritoneal dialysis patients

Graphical abstract

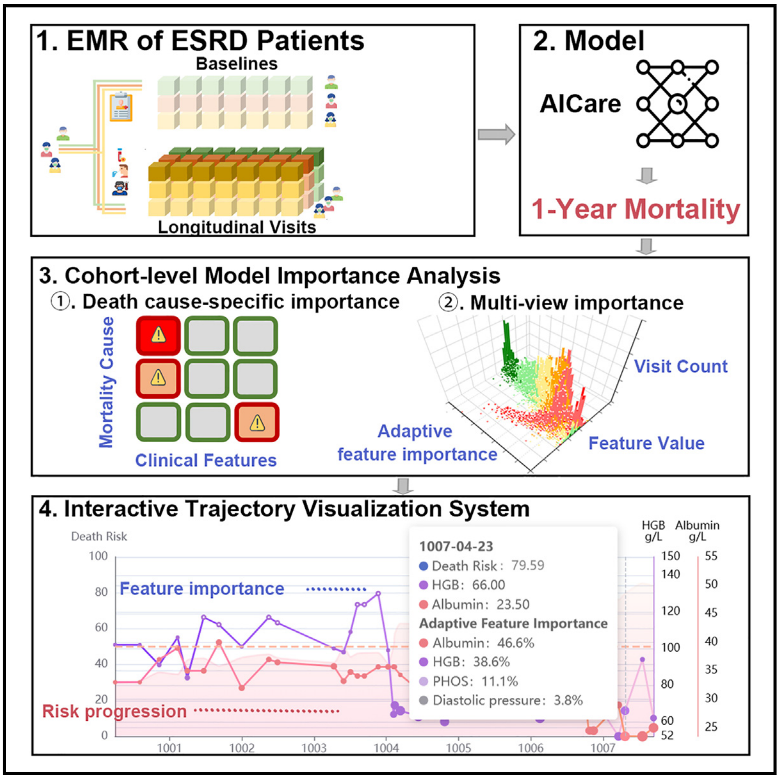

Highlights

- Harnessing deep learning for PD patients' 1-year mortality prediction
- Model clarifies medical indicators affecting mortality outcomes
- AI-doctor system visualizes individual health trajectories

Authors

Liantao Ma, Chaohe Zhang, Junyi Gao, ..., Xinju Zhao, Wenjie Ruan, Tao Wang

Correspondence

junyii.gao@gmail.com (J.G.),
wangyasha@pku.edu.cn (Y.W.),
tanggwen@126.com (W.T.)

In brief

As global health concerns shift with aging populations, the looming threat of kidney diseases necessitates better treatment management. This study introduces an AI-driven model adept at predicting critical health risks for kidney disease patients undergoing peritoneal dialysis. By not just predicting but also explaining these risks, the research paves the way for timely, personalized medical interventions.

Ma et al., 2023, Patterns *4*, 100892
December 8, 2023 © 2023 The Authors.
https://doi.org/10.1016/j.patter.2023.100892

CellPress

Patterns

CellPress
OPEN ACCESS

Article

Mortality prediction with adaptive feature importance recalibration for peritoneal dialysis patients

Liantao Ma,[1,7] Chaohe Zhang,[1,7] Junyi Gao,[2,6,7,*] Xianfeng Jiao,[1] Zhihao Yu,[1] Yinghao Zhu,[1] Tianlong Wang,[1] Xinyu Ma,[1] Yasha Wang,[1,8,*] Wen Tang,[3,*] Xinju Zhao,[4] Wenjie Ruan,[5] and Tao Wang[3]
[1]Peking University, Beijing, China
[2]Centre for Medical Informatics, University of Edinburgh, Edinburgh, UK
[3]Department of Nephrology, Peking University Third Hospital, Beijing, China
[4]Department of Nephrology, Peking University People's Hospital, Beijing, China
[5]Department of Computer Science, University of Exeter, Exeter, UK
[6]Health Data Research UK, London, UK
[7]These authors contributed equally
[8]Lead contact
*Correspondence: junyii.gao@gmail.com (J.G.), wangyasha@pku.edu.cn (Y.W.), tanggwen@126.com (W.T.)
https://doi.org/10.1016/j.patter.2023.100892

THE BIGGER PICTURE As global populations age and their lifestyles change, the threat of end-stage renal disease (ESRD) grows ever more significant. Consequently, an increasing number of patients require life-sustaining treatments such as peritoneal dialysis (PD). For these PD patients, their medical journey involves more than just treatment, it is about comprehending the trajectory of their health, navigating potential health risks, and underlining the urgent need for real-time, personalized risk predictions. We employ deep learning not only to predict but also to comprehend the mortality risks associated with PD patients. Our model doesn't merely "tell"; it "explains." It offers clinicians insight into the reasons behind its predictions by highlighting crucial medical factors that shape these outcomes. Additionally, we created a functional AI-doctor interaction system, empowering professionals to visualize a patient's health trajectory and grasp the personalized reference values of clinical indicators.

1 2 3 4 5 **Development/Pre-production:** Data science output has been rolled out/validated across multiple domains/problems

SUMMARY

The study aims to develop AICare, an interpretable mortality prediction model, using electronic medical records (EMR) from follow-up visits for end-stage renal disease (ESRD) patients. AICare includes a multichannel feature extraction module and an adaptive feature importance recalibration module. It integrates dynamic records and static features to perform personalized health context representation learning. The dataset encompasses 13,091 visits and demographic data of 656 peritoneal dialysis (PD) patients spanning 12 years. An additional public dataset of 4,789 visits from 1,363 hemodialysis (HD) patients is also considered. AICare outperforms traditional deep learning models in mortality prediction while retaining interpretability. It uncovers mortality-feature relationships and variations in feature importance and provides reference values. An AI-doctor interaction system is developed for visualizing patients' health trajectories and risk indicators.

INTRODUCTION

The prevalence of end-stage renal disease (ESRD) continues to increase and has become a significant healthcare burden worldwide. Approximately 3.8 million people currently rely on some form of dialysis for the treatment of ESRD worldwide.[1] ESRD is a long-term disease, and patients need continuous medical care and treatment for years or even decades. Peritoneal dialysis

This is an open access article under the CC BY license (http://creativecommons.org/licenses/by/4.0/).

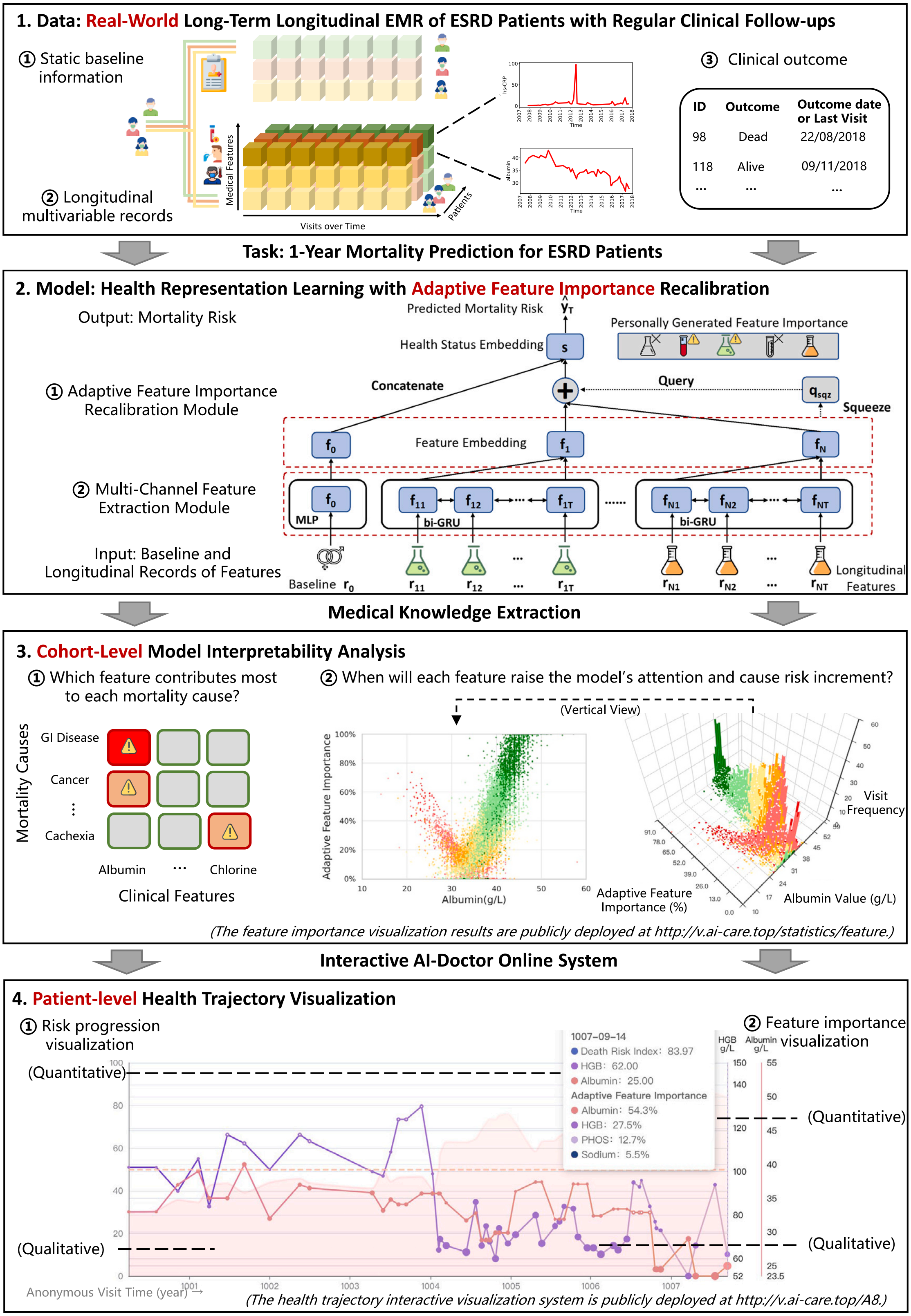

(legend on next page)

(PD) is a well-established renal replacement therapy (RRT) modality and the leading form of home-based life-supporting dialysis therapy for patients with ESRD.[2] Over the past decade, the use of PD increased dramatically worldwide.

During long-term PD, patients may still encounter various vital risks, such as cardio-cerebrovascular disease and infection.[3] These risks may cause adverse outcomes, and patients need lifelong treatment with periodic follow-up visits to monitor their health status. Predicting mortality risk and identifying modifiable risk factors from routine clinic visit records are of great importance for personalized medicine and early intervention to prevent adverse outcomes and improve the survival of long-term PD patients. Recent studies have attempted to utilize artificial intelligence (AI) techniques to evaluate the health status of patients. However, there are still some critical issues that have not yet been thoroughly addressed by existing works.

Issue$_1$: Perform dynamic mortality prediction at each follow-up visit based on the effective utilization of both sequential medical records and the baseline demographic information

Most AI-based electronic medical record (EMR) analysis research on kidney disease patients only uses static baseline information to perform one-time health prediction based on traditional machine learning methods.[4–13] These methods cannot perform real-time health prediction, and thus the practical utility in the clinical application is limited. Other research models the disease process by incorporating sequential EMRs.[14–16] However, these models cannot yet effectively together embed the baseline information and the sequential records and capture the interaction between them during the health status embedding procedure, which leads to limited prediction performance.

Issue$_2$: Provide fine-grained interpretability for each patient individually by selecting key features that contribute the most to mortality prediction (patient-level interpretability) and simultaneously achieve high prediction performance

Key factors strongly indicate that health risk are different among patients. Medical experts need to understand how a model makes a specific decision for a particular patient. This requires sufficient model interpretability to ensure that prediction results are trustworthy for developing bespoke interventions and extracting medical knowledge. However, most existing studies fail to ensure the model's trustworthiness in providing verifiable interpretations. On one hand, traditional machine learning models, such as decision trees,[7,8,11,12,17] are clinically interpretable, but they cannot capture complex longitudinal progressions and thus have inferior prediction performances. On the other hand, the decision-making process in deep learning-based models is a black box and fails to provide human-understandable interpretation.[13,14] Some recent works apply the Shapley additive explanation (SHAP),[18,19] feature permutation,[9,10] and inverse analysis[20] strategies to improve the interpretability. However, these post hoc interpretation[21] methods can only provide coarse-grained interpretability, which is difficult to understand at the patient level. It is still challenging to simultaneously provide satisfactory interpretability and achieve high prediction performance.

Issue$_3$: Adaptively analyze the importance of each feature along with the variation of its value (feature-level interpretability) to provide medical advice and extract knowledge

The way of attending to the medical feature in the prediction process should be flexible and individualized according to its value. However, most existing studies analyze the health status of patients in a fixed decision process[7,12,17] or embed clinical features via fixed parameters of neural networks without ante hoc interpretability.[9,13,20] To the best of our knowledge, none of the existing AI-based clinical prediction studies for kidney disease patients explicitly analyze the changes in the feature importance with features' values.

To address these challenges, we propose a deep learning-based interpretable mortality risk prediction framework for PD patients, AICare. As shown in Figure 1, it is built upon a real-world longitudinal EMR dataset of PD patients spanning 12 years, including baseline demographic information and outcomes, as well as patient-level follow-up lab tests and diagnosis records spanned by an average of 20 visits per patient. The main contributions of this work are summarized below.

(1) Our proposed framework, AICare, models the health trajectory based on multivariate EMR data of PD patients and achieves better prediction performance than state-of-the-art (SOTA) methods while simultaneously providing fine-grained patient-level interpretability. As shown in Figure 1 section 2, AICare employs a multichannel medical feature embedding architecture to extract sequential patterns from high-dimensional medical features. The squeezed embedding of static information and dynamic features is treated as a health context vector to guide the feature importance recalibration (addressing Issue$_1$). AICare assigns attention weights for each feature by looking at the health context for clues that can help lead to a more individual representation of the health status. On the dynamic mortality prediction tasks, AICare achieves 47.2% AUPRC (area under the precision-recall curve) on the PD dataset, which is 11.8% relatively higher than the SOTA comparative baseline model. We also introduce an additional experiment dataset, which is described in the supplemental information.

(2) AICare provides an elucidation of the relationship between the causes of mortality in patients with PD and clinical

Figure 1. Mortality prediction research overview for peritoneal dialysis (PD) patients

(1) We collect an over 12-year, long-term, and real-world clinical EMR dataset of PD patients, consisting of static baseline information, longitudinal multivariable records, and clinical outcomes. The prediction task is defined as a 1-year mortality prediction at each clinical visit. (2) We propose a deep learning-based interpretable health status representation learning framework consisting of a multichannel feature extraction module and an adaptive feature importance recalibration module. (3) We perform a model interpretability analysis for diverse mortality causes and observe the change of feature importance to extract novel medical knowledge (taking albumin as an example). (4) We build an interactive AI-doctor system to visualize the health trajectory.

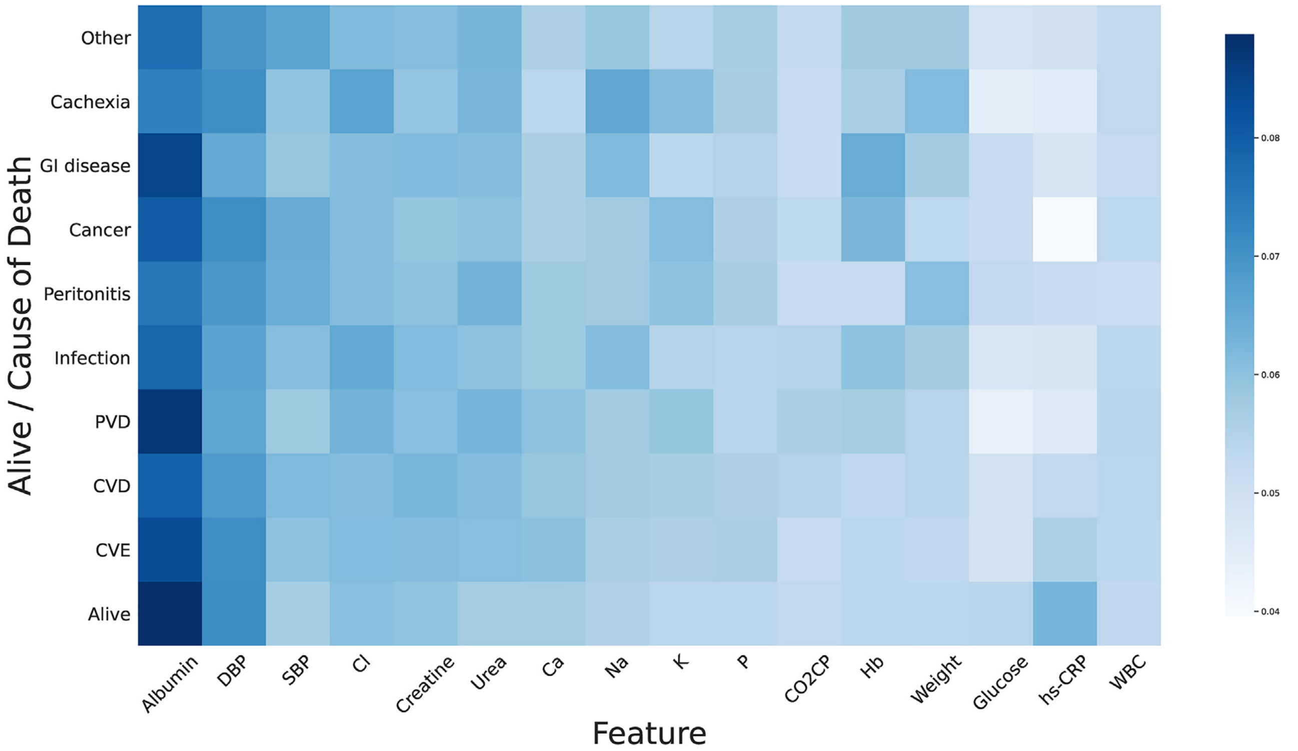

Figure 2. Average feature importance heatmap for diverse mortality causes generated by AICare

The darker the color, the greater the importance. Serum albumin is the most important feature in mortality prediction, especially for PD patients who died of gastrointestinal (GI) disease or peripheral vascular disease (PVD). Diastolic blood pressure (DBP) is the second indicative feature, especially for PD patients who died of cachexia, cancer, or cerebrovascular disease (CVE).

features (patient-level interpretability in Issue_2) based on an end-to-end deep learning model. As shown in Figure 1 section 3.1 and Figure 2, AICare achieves fine-grained interpretability by adaptively emphasizing high-risk features during the prediction process based on a feature recalibration module. We report detailed patient-level interpretability analyses; serum albumin, diastolic blood pressure, and chlorine are the most important indicators for most PD patients. Albumin is a strong indicator for patients who died of gastrointestinal disease and peripheral vascular disease. Diastolic blood pressure (DBP) is an indicator for patients who died of cachexia, cancer, and cerebrovascular disease. Systolic Blood Pressure (SBP) is indicative of cancer and PD-associated peritonitis deaths.

(3) AICare reveals the variation pattern in each feature's importance for PD patient mortality prediction (feature-level interpretability in Issue_3). As shown in Figure 1 section 3., Figures 3 and 4, and Table 5, AICare provides the ante hoc attention weight of each clinical feature according to its value and the patient's condition. We report detailed feature-level interpretability analyses. There are two variation patterns of importance in medical features: V-shaped parabolic curves (e.g., albumin and DBP) and L-shaped fold lines (e.g., SBP and hemoglobin). For example, the importance weight of albumin is presented as a V-shaped curve with 32 g/L as the lowest turning point. For most PD patients, when albumin is lower (higher) than the turning point of 32 g/L, the more extreme the value, the more attention weight is assigned by AICare, which means that this feature plays an essential role in the health status representation learning, and the predicted mortality risk rises (declines). AICare recommends improving albumin to higher than 32 g/L—the higher the better. The importance weight of SBP presents as an L-shaped curve with 130 mm Hg as a turning point. For SBP over 130 mm Hg, AICare pays nearly no attention to SBP. AICare recommends raising the SBP to at least 130 mm Hg for most PD patients, but a further increase in SBP will not bring many benefits.

(4) We develop a practical AI-doctor interaction system to visualize the trajectory of patients' health status and risk indicators. Model deployment has been the last but most challenging step toward clinical application. Deploying the deep models in an accessible way for clinicians to allow them to easily understand model predictions and model decision process still needs extra considerations. As shown in Figure 1 section 4, to further facilitate personalized clinical service, we deploy an AI-doctor interaction system online with open-source code at https://github.com/Accountable-Machine-Intelligence/AICare. Our developed health trajectory visualization system with anonymous case studies (patient IDs A1–A20) is publicly available at http://v.ai-care.top/A8. Visualization of the importance of the features is available at http://v.ai-care.top/statistics/feature. Users can upload the data online to get the

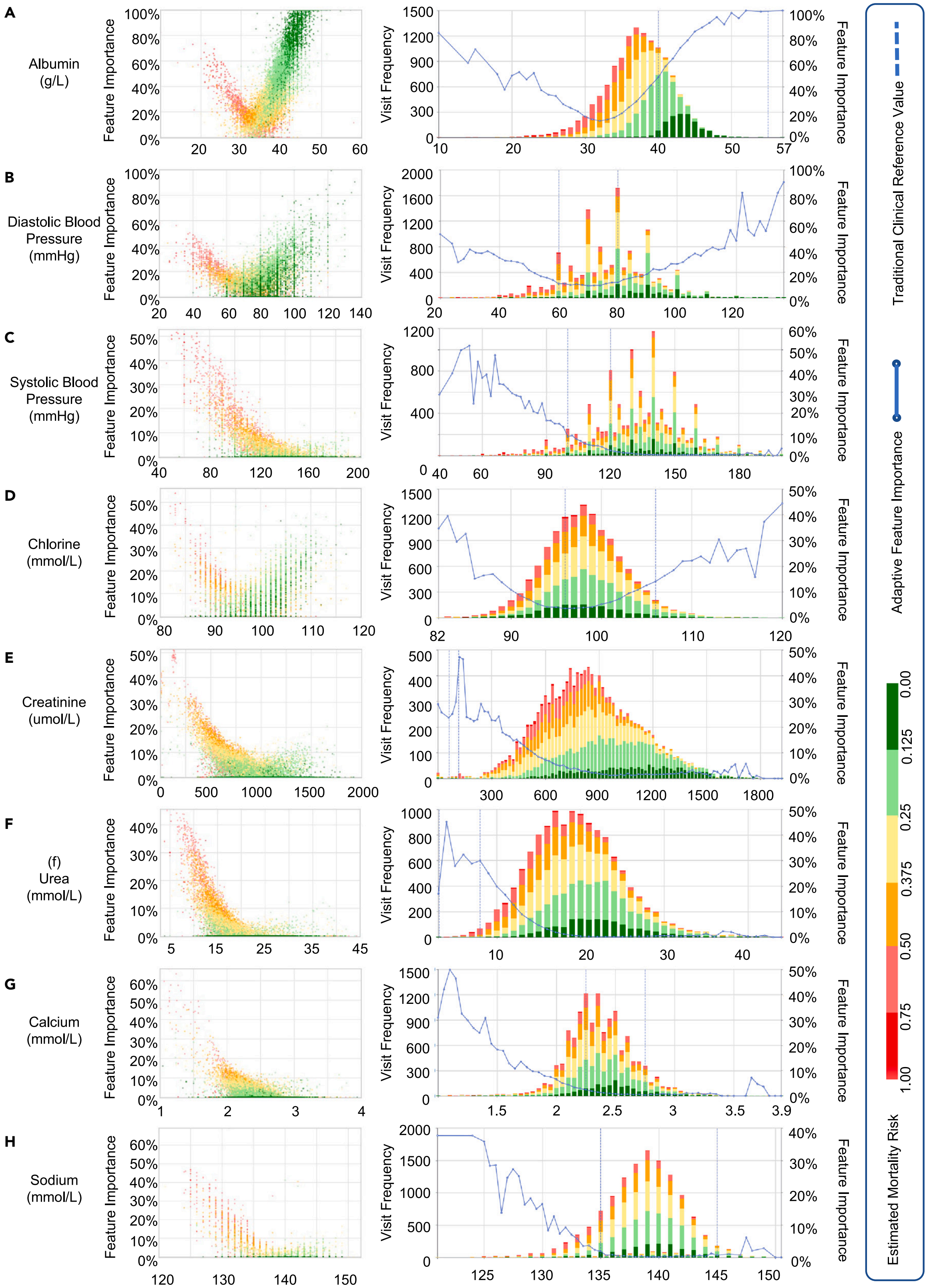

Figure 3. Feature importance variation learned by AICare (features a–h)

The clinical visits are marked as colored dots and histograms. Red represents high risk predicted by AICare, while green represents low risk. The average feature importance is visualized as blue fold lines. The traditional clinical reference values are vertically marked as blue dotted lines. There are two variation patterns of

(legend continued on next page)

Table 1. Statistics of baseline information and label assignment

	Total	Mortality (%)	Survival (%)
Patients	656	261 (39.8%)	395 (60.2%)
Visits	13,091	1,196 (9.1%)	11,895 (90.9%)
Age			
16–40	96 (14.6%)	10 (10.4%)	86 (89.6%)
40–60	217 (33.1%)	64 (29.5%)	153 (70.5%)
60–80	297 (45.3%)	153 (51.5%)	144 (48.5%)
80–98	44 (6.7%)	33 (75.0%)	11 (25.0%)
Diabetes			
Patients with diabetes	244 (37.2%)	120 (49.2%)	124 (50.8%)
Gender			
Female	327 (49.8%)	125 (38.2%)	202 (61.8%)
Male	328 (50.2%)	136 (41.5%)	192 (58.5%)

The real-world dataset contains 656 peritoneal dialysis (PD) patients with 13,091 clinical visits. There are 39.8% patients who died before the final follow-up. The age range of patients enrolled is 16–98 years.

prediction results immediately (http://v.ai-care.top/predict) or download the code to train the model based on their dataset offline. We made an abstract presentation video introducing our work (https://youtu.be/CY2glHchsC8).

RESULTS

Data description and problem formulation

We collected the EMR data of 656 PD patients with 13,091 visit records, spanning over 12 years, from January 1, 2006, to January 1, 2018, including patients' baseline data, longitudinal visit records, and outcomes.

(1) Baseline data include patients' demographic data (e.g., age and gender) and the diagnosis of diabetes at the beginning of dialysis. The statistics of the baseline data and the assignment of the labels are shown in Table 1.
(2) Visit data include laboratory tests and patients' vital signs at each visit. The visit frequency statistics and the feature values distribution are shown in Tables 2 and 3, respectively.
(3) Outcome data include patients' outcomes at the end of the data collection window, including death date and cause of death (e.g., cancer). The outcomes of all patients were followed and further recorded until October 31, 2018.

Table 2. Statistics of age and visit frequency

Statistic	Avg	Med	Max	Min	Std
Age (year)	58.55	60.70	97.45	16.79	15.81
Visits per patient	19.95	16	69	1	13.53
High-risk visits per patient	2	0	29	0	2.95
Duration of follow-up (year)	3.98	3.43	10.44	0.1	2.67
Visit interval (month)	2.73	2.48	29.87	–	2.67

Peritoneal dialysis (PD) patients were followed up every 3 months. There are about 20 visits recorded for each patient. Avg, average; Med, median; Max, maximum; Min, minimum; Std, standard deviation.

The feature sets consist of 16 longitudinal medical features and 4 baseline features. The age distribution of the patients is 58.55 ± 15.81 years, and the number of average records per patient is 19.95 ± 13.53. We fill in the missing values with the most recent historical recorded values.

We conduct the 1-year dynamic mortality prediction task. Given a patient's visit records with T visits, the binary classification task is to predict the mortality risk in the future 1 year $\widehat{y}_t$ at each visit t. To meet actual clinical practice, we also define an uncertain phase of patient health status. For patients with negative labels (alive, $y = 0$), the uncertain phase is 1 year before the end date of data collection because we do not know the outcomes of these patients in the future 1 year. For patients with positive labels (dead, $y = 1$) at t, the uncertain phase is between the $t-2$ year and the $t-1$ year because we are uncertain about the ground-truth health status during these visits. The final dataset contains 1,196 visits with positive labels (i.e., died within 1 year) and 10,804 records with negative labels. For more details about the dataset and the problem formulation, please see the supplemental information.

Prediction performance

The prediction performance of AICare and the baseline models on the 10-fold cross-validation mortality prediction of PD patients, evaluated by AUPRC and area under the receiver operating characteristic curve (AUROC), are shown in Table 4. AICare achieves 47.2% AUPRC, which is relatively 11.8% higher than the best baseline model.[1] This indicates that AICare can efficiently embed the long-term longitudinal multivariable sequential data and static baseline data to learn the representation of the health status of PD patients individually, using the multichannel feature extraction module and the adaptive feature importance recalibration module. More details about the experiment are listed in the supplemental information, including the prediction performance of diverse mortality causes and detailed descriptions of the comparative baseline methods. To verify the application universality of AICare on other patient cohorts, we also

feature importance: V-shaped parabolic curves (e.g., albumin, DBP) and L-shaped fold lines (e.g., SBP and Hb). We take the serum albumin importance variation as an example of a V-shaped parabolic curve. For most patients, when albumin is lower (higher) than the turning point of 32 g/L, the more extreme the value, the more attention weight is assigned by AICare, which means this feature takes essential part in health status representation learning, and the predicted mortality risk rises (declines). As a result, AICare recommends improving the serum albumin to above 32 g/L—the higher the better. On the contrary, we take the systolic blood pressure (SBP) importance variation as an example of an L-shaped fold line. For most patients, when SBP is lower than the turning point of 130 mm Hg, the lower the value, the more attention weight is assigned. However, when SBP is higher than 130 mm Hg, the attention weight drops to nearly 0%, meaning this feature will no longer affect the representation learning of health status. As a result, AICare recommends improving the SBP to at least 130 mm Hg, but higher SBP will not bring many benefits.

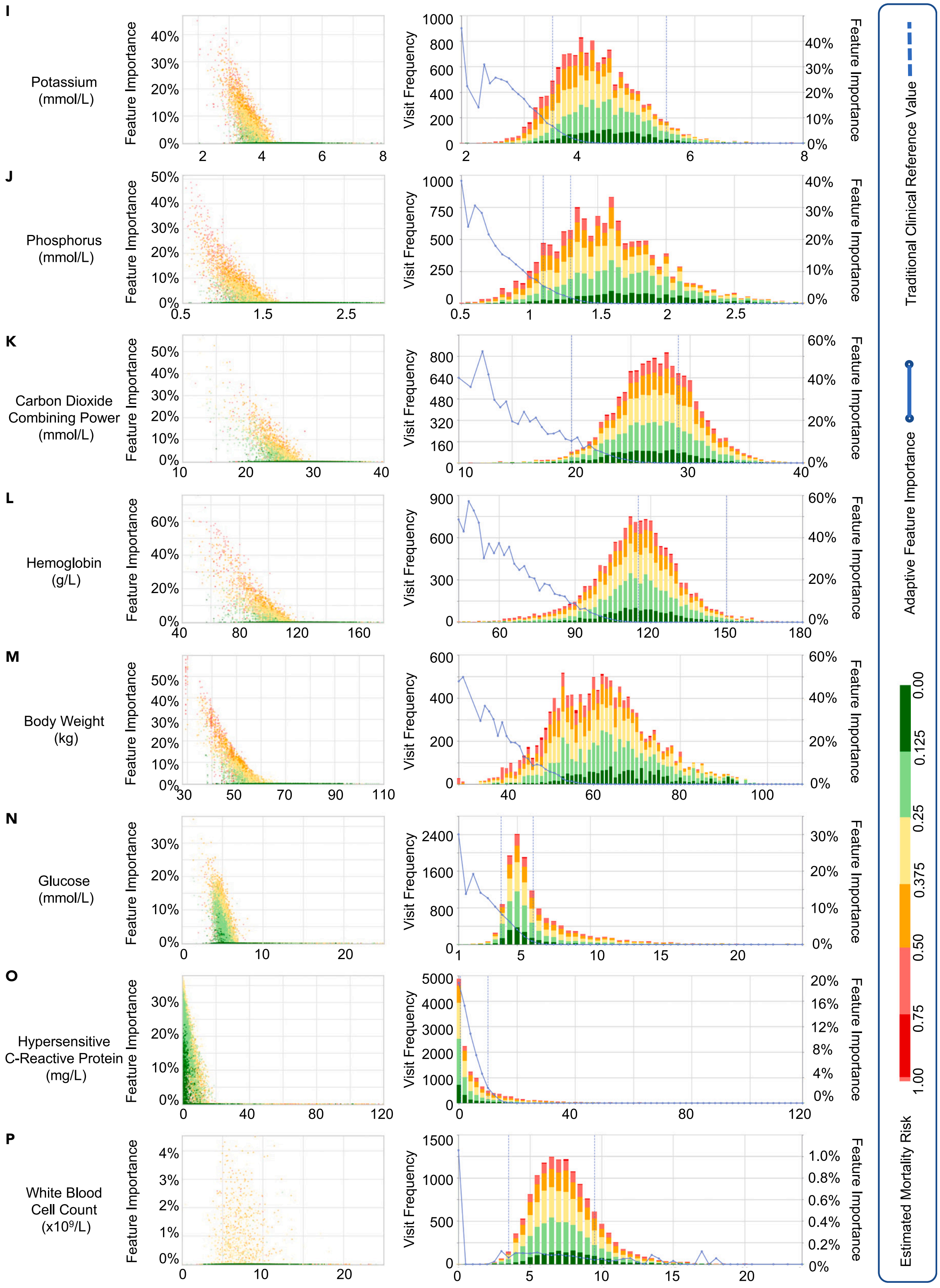

Figure 4. Feature importance variation learned by AICare (features i–p)

Table 3. Feature summary of the peritoneal dialysis (PD) dataset

Abbreviation	Full name	Unit	High-risk visits ($y = 1$)			Low-risk visits ($y = 0$)			Missing
Dynamic Features			Mean	Std	Med	Mean	Std	Med	
Albumin	albumin	g/L	33.81	4.437	34.3	37.87	4.337	38	25%
DBP	diastolic blood pressure	mm Hg	70.28	14.71	70	78.59	13.79	80	18%
SBP	systolic blood pressure	mm Hg	125.3	25.19	127	134.4	21.61	135	14%
Cl	chlorine	mmol/L	96.02	4.155	96	98.21	4.923	98	17%
Cr	creatinine	μmol/L	779.6	250.3	741	868.9	270.3	853	10%
Urea	urea	mmol/L	18.12	5.545	17.8	20.09	5.363	19.8	11%
Ca	calcium	mmol/L	2.358	0.277	2.345	2.406	0.341	2.39	12%
Na	sodium	mmol/L	137.1	4.262	137.9	138.5	4.617	139	21%
K	potassium	mmol/L	4.240	0.783	4.17	4.320	0.718	4.25	11%
P	phosphorus	mmol/L	1.549	0.450	1.5	1.606	0.430	1.57	13%
CO_2 CP	CO_2 combining power	mmol/L	27.45	3.562	27.5	27.38	3.630	27.4	8%
Hb	hemoglobin	g/L	111.4	19.54	113	114.6	17.05	115	12%
Weight	body weight	kg	59.98	11.05	59.59	62.26	11.07	62	41%
Glucose	glucose	mmol/L	7.758	3.665	6.7	6.689	3.089	5.7	30%
hs-CRP	hypersensitive C-reactive protein	mg/L	17.57	28.07	8.49	7.954	13.96	3.19	29%
WBC	white blood cell count	$\times 10^9$/L	8.238	2.767	7.895	7.773	2.754	7.43	10%
Baseline features									
Age	age	year	66.12	13.01	67.82	53.30	15.54	54.53	0%
Gender	female (0) or male (1)	–	0.53	0.50	1	0.49	0.50	0	0%
Height	height	cm	162.2	9.95	160.5	164.1	10.98	163.8	0%
Diabetes	is (1) or is not (0) diabetic	–	0.45	0.50	0	0.31	0.46	0	0%

This dataset comprises 16 dynamic features recorded at each clinical visit and 4 static baseline features recorded at the first visit. Med, median; Std, standard deviation.

introduce an additional experiment dataset to train the model and test the prediction performance, which consists of 1,363 ESRD patients receiving hemodialysis (HD) from another grade A tertiary hospital. The prediction performance is listed in the supplemental information.

Table 4. Mortality prediction performance of PD patients

Method	AUPRC	AUROC
GRU (gated recurrent unit)[22]	0.422 (0.109)	0.781 (0.047)
Transformer[23]	0.406 (0.097)	0.789 (0.047)
MT-RHN (multi-task recurrent highway network)[15]	0.413(0.107)	0.777(0.063)
LSTM (long short-term memory network)[19]	0.395(0.100)	0.782(0.065)
biLSTM-FC (bidirectional LSTM with fully connected layer)[24]	0.398(0.089)	0.758(0.067)
LR (logistic regression)[5]	0.370 (0.084)	0.610 (0.044)
XGBoost[17]	0.379 (0.087)	0.597 (0.033)
DT (decision tree)[12]	0.319 (0.040)	0.607 (0.027)
LightGBM[18]	0.405 (0.082)	0.604 (0.028)
AICare	0.472 ** (0.075)	0.816 ** (0.033)

Our proposed model, AICare, outperforms other comparative baseline approaches, including deep models. The values in parentheses are the standard deviation of 10-fold cross-validation. **: $p<0.01$.

Interpretability analysis

AICare provides fine-grained interpretability to help clinicians understand the prediction decision process. At each visit, the model provides dynamic importance weights for the features that indicate the contributions of each feature to the final prediction result. In this section, we discuss the detailed interpretability analyses.

Average feature importance for diverse causes of death

We calculate the average importance of each feature for patients, which is shown as a heatmap in Figure 2. The results indicate that serum albumin, DBP, and chlorine (Cl) are considered important health indicators for most PD patients because their columns are darker than other feature columns. Some findings of the relationship between the causes of mortality in PD patients and clinical features are listed below.

Albumin is the strongest indicator of most causes of death, especially for cerebrovascular disease (CVE), peripheral vascular disease (PVD) and gastrointestinal (GI) disease, according to the heatmap generated by AICare. This may be because albumin is an indicator of protein energy wasting, correlated with suboptimal GI intake and inflammation.[25,26] Hypoalbuminemia is a strong predictor for PD-related peritonitis,[27] which is the primary reason for deaths from infection and peritonitis. Besides,

CellPress
OPEN ACCESS

Table 5. Importance variation pattern and recommended reference value (turning point) learned by AICare for PD patients

Feature	Unit	Importance variation learned by AICare			Traditional reference range		Consistency
		Variation type	Recommendation	Turning point	Lower limit	Upper limit	
Albumin	g/L	V shape	higher	>32	40	55	√
DBP	mm Hg	V shape	higher	>70	60	80	~
SBP	mm Hg	L shape	at least	>130	100	120	×
Cl	mmol/L	V shape	higher	>96	96	106	√
Cr	μmol/L	L shape	at least	>900	62	115	×
Urea	mmol/L	L shape	at least	>20	3.1	9	×
Calcium	mmol/L	L shape	at least	>2.5	2.25	2.75	~
Na	mmol/L	L shape	at least	>135.5	135	145	√
K	mmol/L	L shape	at least	>4	3.5	5.5	√
P	mmol/L	L shape	at least	>1.5	1.1	1.3	×
CO2CP	mmol/L	L shape	at least	>25	20	29	~
Hb	g/L	L shape	at least	>114	115	150	√
Weight	kg	L shape	at least	>59	–	–	–
Glucose	mmol/L	L shape	not exceed	<6	3.9	6.1	√
Hs-CRP	mg/L	L shape	not exceed	<16	0.5	10	√
WBC	$\times 10^9$/L	irregular	unknown	–	3.5	9.5	–

This table is a quantified summary of Figures 3 and 4. Recommendation "higher" means that AICare suggests increasing this feature's value above the turning point. "At least" means that AICare suggests maintaining the value above the turning point, but a further increase may not bring many benefits. Consistency (~) means that there is some overlap between the reference range recommended by AICare for PD patients and the traditional reference range for outpatients. We have publicly deployed the visualization charts of the variation in importance of features at http://v.ai-care.top/statistics/feature.

our model generates a high attention weight of albumin for patients who are still alive, which means that low-risk scores are associated with high albumin value. More details about albumin can be found in the supplemental information.

DBP is a risk indicator for CVE, PD-related peritonitis, cancer, and cachexia deaths. This may be because DBP is a marker of atherosclerosis and is strongly independently related to atherothrombotic brain infarction incidence.[28] Low DBP could also be an indicator for low peripheral vascular resistance or increased arterial stiffness[29,30] that is strongly associated with a high incidence of cardio-cerebral vascular disease.[31] Additionally, low blood pressure (BP) is a surrogate predictor for specific comorbidities, heart failure, chronic inflammation, and malnutrition,[32] which may be related to death from peritonitis, cancer, and cachexia.

Sodium (Na), potassium (K), and body weight are important indicators for cachexia deaths. This may be because patients with cachexia often experience low Na and K levels due to insufficient food intake. Decreasing weight for these patients is a common phenomenon.

Hemoglobin (Hb) is an important indicator for GI disease deaths. GI bleeding is a critical manifestation of uremic GI disease. Hb and K are indicators for cancer deaths, which are consistent with the fact that cancer is highly associated with refractory anemia, anorexia, and, consequently, hypokalemia due to insufficient intake.

Urea, body weight, K, albumin, DBP and SBP are important indicators for PD-related peritonitis deaths. The risk factors for peritonitis, a common complication of PD patients, have been well defined[27] and include hypoalbuminemia, hypokalemia, protein energy wasting, etc. This is consistent with the results of our model.

Change of feature importance with feature values

AICare quantifies the feature importance changes with feature values in a macroscopic perspective for the whole patient cohort to help clinicians better understand the decision process, perform individualized intervention, and extract new medical knowledge, as shown in Figures 3 and 4.

In the left scatterplot, the x axis denotes the value of the biomarker. The y axis denotes the feature's importance. Each dot represents a follow-up visit of a patient, and the color represents the predicted risk. The right histogram shows the risk distributions at different values of biomarkers. The blue curve is the fitted curve of the average importance of the feature. We also plot each feature's traditional clinical reference ranges for normal clinic outpatients as blue dotted lines, helping physicians evaluate the consistency between the results of AICare and the traditional ranges.

There are two obvious patterns of relationships between biomarkers' importance weights and recorded values: a V-shaped parabolic curve and an L-shaped fold line. For the V-shaped parabolic pattern (e.g., albumin and DBP), an extremely high or low feature value will cause high importance attention weight through AICare, which means the feature plays an essential part in the learning the representation of health status. For the L-shaped fold line pattern (e.g., SBP and Hb), the lower the biomarker value, the higher the importance of attention weight.

The pattern of variation in importance and the recommended reference values learned by AICare are summarized in Table 5. We will discuss these patterns in detail in the following text.

(1) Albumin (Figure 3A). AICare believes that the albumin importance attention weight appears to be a V-shaped

curve with 32 g/L as a turning point. The variation of albumin in a descending or ascending manner always gets the model's attention. Considering that the red dots are mostly on the left side of the figure, AICare learns that patients with an albumin level lower than 32 g/L tend to have a high importance weight and poor prognosis ($y > 0.5$). When the albumin level is lower than 23 g/L, more than 50% attention weight is given, which means that the albumin level becomes the most critical indicator for the 1-year mortality outcome.

On the other hand, between the range of 32 and 57 g/L, a high albumin value also causes high importance weight and indicates a significant improvement in the patient's health ($y < 0.5$). When the albumin level is higher than 40 g/L, it often occupies about 50% to even 100% of the feature importance weight, which means the model can predict the high survival expectation of patients using just this feature. As a result, AICare recommends raising the albumin level to above 32 g/L as much as possible for most PD patients.

The traditional clinical reference range of albumin for outpatient clinics is 40–55 g/L, which is highly consistent with the recommended range given by AICare. This finding is also consistent with a recent study that evaluated the association between serum albumin trajectories and mortality in PD patients using the joint modeling approach, showing that changes (increases and decreases) in serum albumin over time were strongly and significantly associated with mortality after adjustment for the risk factor.[33].

(2) DBP (Figure 3B). DBP is another critical feature in the evaluation of patient health status. Similar to albumin, both high and low levels of DBP will affect the model's attention. The importance weight of DBP varies in a V-shaped curve with 70 mm Hg as a turning point. In the 40–70 mm Hg range, the model pays more attention to DBP as it gets lower and predicts a poor prognosis. When the DBP is below 40 mm Hg, it takes more than 30% of the model attention weights. Most patients whose DBP is below 60 mm Hg are more likely to have a high health risk, marked as red dots in the figure.

On the other hand, in the range of 70–120 mm Hg, the model pays more attention as the DBP gets higher and predicts better prognosis outcomes. When DBP is above 85 mm Hg, it also occupies about 20% of model attention weights, and patients are predicted to have a low-risk condition for most cases, marked as green dots in the figure. As a result, AICare recommends increasing the DBP to above 70 mm Hg, while a greater DBP indicates lower risk.

This is consistent with recent studies of dialysis patients. Higher DBP was associated with decreased early mortality in the first year after the start of RRT.[34] All-cause mortality risk was minimal at 77 mm Hg for DBP in 7,335 Chinese PD patients.[35] DBP lower than 70 mm Hg may be related to an increased mortality risk in both nondiabetic patients with chronic kidney disease and HS patients.[35–38] The traditional normal reference range of DBP for outpatient clinics is 60–80 mm Hg, while maintaining DBP at a relatively higher level is conducive to improving the survival of PD patients. Further research about DBP for PD patients is needed.

(3) SBP (Figure 3C). Unlike the features discussed above, AICare believes that SBP is a typical feature whose importance weights vary in an L-shaped fold line with 130 mm Hg as a turning point, meaning that the importance weights decrease as the value increases. For SBP below 60 mm Hg, AICare gives more than 50% attention, and in most cases, patients are likely to be predicted to have poor outcomes, presented as red dots in the figure. For SBP over 130 mm Hg, AICare pays nearly no attention to SBP ($\alpha < 1\%$), which means that SBP does not affect health status representation learning. As a result, AICare recommends maintaining the SBP at 130 mm Hg or slightly higher for most PD patients. A further improvement over 130 mm Hg does not significantly help reduce mortality risk.

This is consistent with clinic experience and most of the recent studies. Lower BP was a surrogate marker for severe comorbid conditions (e.g., heart failure or ischemic heart disease), chronic inflammation, and malnutrition and, hence, can lead to worse outcomes by limiting blood flow to vital organs.[35,39] The traditional reference range of SBP for outpatient clinics is 100–120 mm Hg. Although accepted definitions of hypertension and BP treatment targets in the dialysis population have not been determined, and definitive recommendations regarding BP treatment targets in dialysis patients have not been made, it is clear that hypotension should be avoided.[40]

(4) Creatinine (Cr) (Figure 3E). The importance variation curve of serum Cr is also L shaped, similar to SBP. For Cr levels in the 160–900 μmol/L range, the lower the level, the more attention is paid by AICare. When the Cr level drops below 400 μmol/L, the model provides more than 15% of attention weights, and the patients are likely to face a poor prognosis ($y > 0.5$). For serum creatinine levels in the range of 900–1750 μmol/L, it often only occupies 5% of attention weights, and patients in this range generally have a lower mortality risk ($y < 0.5$). As a result, AICare recommends maintaining the Cr level at least 900 μmol/L or slightly higher for most PD patients.

This is consistent with the finding of a previous study that a low Cr level (707–815 μmol/L as reference) as a proxy of low muscle mass, nutritional status, and protein energy wasting (PEW) may be associated with adverse outcomes in PD patients.[41,42] In contrast, a high Cr level is associated with a relatively lower mortality risk.[41] Cr should be maintained at a certain level. The traditional reference range of Cr for normal outpatient clinics is 62–115 μmol/L, which is unsuitable for PD patients. Note that AICare provides a rough recommendation for most PD patients in this dataset. We will specify this finding for different cohorts (e.g., different gender) in future work.

(5) Hb (Figure 4L). The curve of the importance variation of Hb is L shaped. The model pays more attention to the Hb level at 44–114 g/L as the Hb level decreases. Hb occupies about 20%–60% of the model attention weights when the Hb level is below 100 g/L. Patients in this range are more likely to have a high mortality risk. The model pays almost no attention to Hb levels above 114 g/L. As

CellPress
OPEN ACCESS

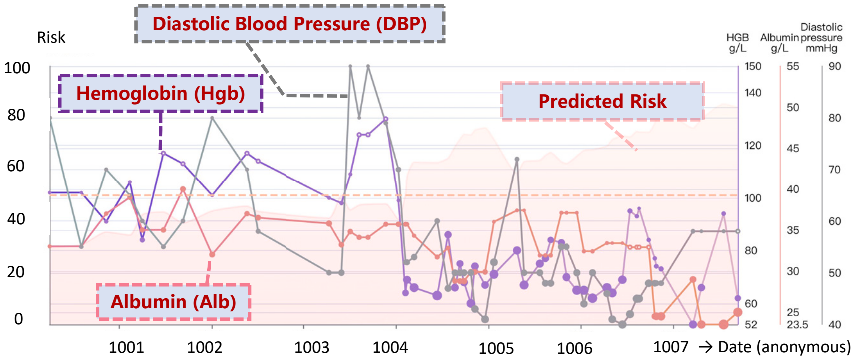

Figure 5. Case study I: Patient died of multiple organ failure

Mortality risk prediction results and interpretability analysis are deployed on a health trajectory interactive visualization system. The x axis denotes the visit date. The y axis denotes the predicted mortality risk (visualized as a pink translucent curve) and feature values. AICare provides the features' importance weights as interpretability at each visit, symbolized as the size of each data point on the line chart and also listed in the inset labels. AICare pays most attention to albumin, Hb, and DBP for this patient, and the patient died of multiple organ failure. The health trajectory interactive visualization system is publicly deployed at http://v.ai-care.top/A8 and is available in English and simplified Chinese.

a result, AICare recommends keeping Hb levels at least 114 g/L, but further increases in Hb may not bring many benefits.

As indicated by a previous study, an Hb level lower than 100 g/L was significantly associated with a higher risk for all-cause and cardiovascular deaths.[43] Lower Hb is also associated with a higher mortality risk in ESA (erythropoiesis-stimulating agents)-treated PD patients.[44] Current anemia management guidelines also suggest not using ESAs to maintain a Hb concentration above 110 g/L in adults.[45]

More analyses about the features' importance are listed in the supplemental information.

Case studies with the health trajectory interactive visualization system

To intuitively show the prediction process and verify the reasonability of AICare when applied to clinical practice, we develop an online AI-doctor system with an interactive interface to visualize the patient's health trajectory with the importance weights of features at each time step. This system makes the prediction results of deep learning models more accessible to clinicians and helps physicians make individualized clinical decisions. We draw a two-dimensional line chart to show the changes in the patient's biomarkers. The x axis is the visit timeline, and the y axis is the value of biomarkers. At each time step, we plot the predicted risk curve $\widehat{y}_i$ (values from 0%–100%). The attention weights of different biomarkers at each time step are also visualized, symbolized as the size of each data point on the line chart. The larger the point, the higher the attention weight.

In the following, we analyze two patient cases using our system. The clinical visit dates were reset to start in 1000 (year) on the online visualization system to protect privacy.Case I

Patient died of multiple organ failure (Figure 5): the first patient died in 1007 (year) due to prostate cancer and multiple organ failure. Figure 5 shows the patient's risk prediction and historical visit information.

During the period the red dotted box covers in the figure, AICare kept predicting a high risk 3 years before the adverse outcome. AICare mainly focused on albumin, DBP, and Hb due to their abnormal values and declining patterns. It is evident that the values of Hb and DBP decreased sharply at the beginning of 1004, which decreased by 69 g/L (from 130 g/L to 61 g/L) and 27 mm Hg (from 79 mm Hg to 52 mm Hg), respectively. AICare sensed the changes rapidly and started to pay attention to them. There was 31.0% of attention given to Hb and 19.8% given to DBP. We can also find a sudden drop of albumin from 32.9 mmol/L to 24.5 mmol/L in 1007 (year), and the albumin level remained at a low level during the last several visits since then, which kept drawing 30%–40% of attention weights of our model.

According to the records, this patient had a series of comorbidities since 1004, including unstable angina pectoris, peripheral arterial disease (PAD), prostate cancer, anemia, diabetic foot, and inflammatory bowel disease, which were closely related to the abnormal biomarkers identified by AICare.

Specifically, the decline of DBP indicated worsening arterial stiffness, which may be associated with severe atherosclerosis, such as coronary heart disease, PAD, and diabetic foot in this patient. The abnormal Hb level indicated deleterious anemia and could be associated with GI bleeding, severe infection, malnutrition, prostate cancer, diabetic foot, and inflammatory bowel disease.[25,26,46] With the help of AICare, physicians may be reminded early to perform a further examination to confirm and treat the these conditions accordingly.Case II

Patient died of digestive system diseases (Figure 6): the second patient was diagnosed with ischemic kidney disease, and PD therapy was initiated. This patient died in December 1004 due to GI disease.

Since July 1003, the risk score generated by AICare increased continuously increased. The attention varied but focused mainly on serum chloride, Na, and urea levels, which were indicators of insufficient intake or GI loss (the details are shown at http://v.ai-care.top/A2). In November 1004, 53.9% of AI attention was assigned to serum albumin (the albumin level decreased from 38 g/L to 20.8 g/L). Finally, during the last visit in December, the risk score for this patient was 90.3, and 66.2% attention was given to the albumin level. AICare captured the most important clinical features related to patient death and generated a timely high risk score.

Materials and ethics issues

This retrospective study was approved by the Medical Scientific Research Ethical Committee. The input of AICare is routinely collected laboratory test results and static baseline information. Patients do not need to conduct any additional unnecessary tests. Our study was granted an exemption from informed

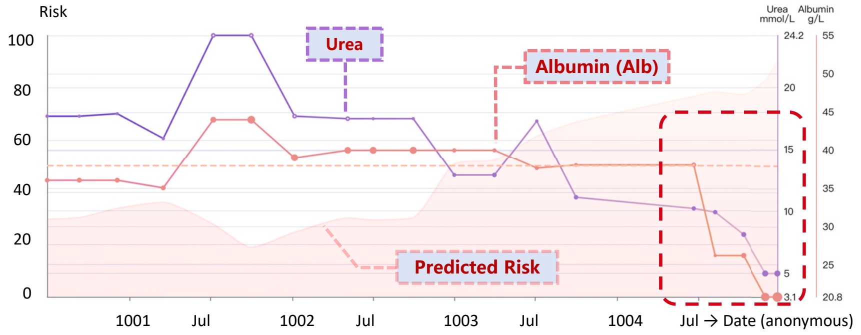

Figure 6. Case study II: Patient died of digestive system disease

AICare pays most attention to albumin and urea for this patient. The patient died of digestive system disease. The health trajectory is shown at http://v.ai-care.top/A2.

consent by the ethics committee due to its retrospective nature and the fact that it did not involve any intervention in the patients' treatment.

Our paper and online system do not contain any sensitive information that could be used to identify individual patients. Patients' private information was anonymized during the analysis. Patient names were replaced with unique patient IDs (e.g., A1, A2). Contact information, including phone numbers and addresses, was deleted. Clinical visit dates were reset to start in 1000 (year) in the online visualization system, and the date of birth was also reset with the corresponding offset.

We developed a practical AI-doctor interaction system to visualize the trajectory of patients' health status and risk indicators. Our developed health trajectory visualization system with anonymous case studies (patient IDs A1–A20) is publicly available at http://v.ai-care.top/A8. Visualization of the importance of the features is available at http://v.ai-care.top/statistics/feature.

All code for the model, the deep learning training parameters, and the training data are openly available. They can be accessed on Zenodo via https://doi.org/10.5281/zenodo.10003570 and http://v.ai-care.top/download. Users can upload data online to get prediction results immediately (http://v.ai-care.top/predict) or download the code to train the model offline based on their dataset. More data that support the findings of this study are available from the corresponding author upon reasonable request.

DISCUSSION

Implications

The whole procedure of PD treatment needs a dynamic prediction of patient mortality risk to help patients prevent adverse outcomes, based on the medical records collected along with the visits. Individual-level dynamic mortality prediction for long-term PD has not yet been substantially studied. Besides, deep models, which can capture complex longitudinal progressions, are often black boxes and fail to provide human-understandable interpretation. Thus, medical professionals lack trustworthiness in deep models.

In this work, we develop a deep learning-based generalizable model capable of learning massive amounts of EMR data and exploring personal characteristics to perform clinical predictions. AICare captures the clinical features that strongly indicate the health status of patients in various conditions. It builds personal health status embedding and provides reasonably fine-grained interpretability in terms of feature importance at each follow-up visit.

In the experiment based on a real-world clinical dataset, 656 incident PD patients were enrolled at the Department of Nephrology of a large grade A tertiary hospital. AICare is used to predict 1-year mortality at each follow-up visit. We compare the performance of AICare with existing related SOTA clinical predictive models. The experiment results show that AICare outperforms the published baseline approaches with 11.8% relative improvement on AUPRC and powerful interpretability.

To facilitate personalized clinical service and verify the reasonability of the model, we develop an AI-doctor interaction system to reveal the patient's health trajectory and the corresponding vital biomarkers while predicting a prognosis. After the trial of our system, experienced nephrology department physicians suggest that AICare can offer opportunities to identify patients with potential mortality risks within a time window that enables early individualized treatment and outcome improvement. The medical knowledge learned by AICare has been positively confirmed by human medical experts and related medical literature.

Key findings and clinical recommendations restatements

Important features summary

Some of the key findings generated by AICare are summarized below. For more details about the medical findings, please see Figures 3 and 4 and Table 5.

Albumin is the most indicative feature for the prediction of 1-year mortality in patients with PD, especially for GI disease, PVD, and living patients. The feature importance weight of albumin presents as a V-shaped curve along with the albumin level. A higher albumin level brings better survival expectations. AICare recommends raising the albumin level to above 32 g/L as much as possible for most PD patients.

DBP is the second important feature. It is indicative especially for cachexia, cancer, CVE and living patients. SBP is indicative for cancer and PD-associated peritonitis (PDAP). The importance weight of DBP and SBP presents as V-shaped and L-shaped curves, respectively. AICare recommends raising the DBP to above 70 mm Hg for most PD patients. AICare recommends maintaining the SBP at least 130 mm Hg. But further increases of SBP will not bring many benefits.

Cl is indicative for cachexia and infection patients. The importance weight of Cl presents as a V-shaped curve. A higher Cl level brings better survival expectations. AICare recommends raising the Cl level to above 96 mmol/L for most PD patients.

Cr is indicative for GI disease and cardiovascular disease (CVD) patients. AICare recommends raising the Cr level to above 900 μmol/L, which is a rough recommendation for most PD patients in this dataset. We will specify this finding for different cohorts (e.g., different gender) in future work.

Urea is indicative for PDAP and PVD patients. AICare recommends raising the urea level to above 20 mmol/L for most PD patients.

Phosphorus (P) is indicative for PDAP, cancer, CVD, and CVE patients. The importance weight of P presents as an L-shaped curve. AICare recommends raising the P level to above 1.5 mmol/L for most PD patients. Further increases will not bring benefits.

Hb is indicative for GI disease patients. The importance weight of Hb presents as an L-shaped curve. AICare recommends raising Hb level above 114 g/L for most PD patients. Further increases will not bring many benefits.

Prediction performance for mortality causes

As shown in Table.S1, experiment results indicate that AICare can effectively predict the most common adverse outcomes of PD patients (e.g., cachexia, PVD, infection, and cancer). However, CVE and CVD are the most challenging mortality causes to predict. CVE[47] patients in particular often acutely suffer from sudden death without apparent signs. This suggests that more frequent clinical follow-up and more clinical tests should be included as features (e.g., ECG examination) to perform early screening for CVE.

Limitations and future work

Introducing multicenter EMRs to increase the data amount

A major limitation of this study is the single-center design, which makes the data amount of the research scarce. The limitation also results in a relatively small sample of positive cases. However, the analyzed data of 656 PD patients with 13,091 visits cover a long-term longitudinal trajectory of PD patients. There are about 20 visits recorded for each patient, with an average visit interval of 2.7 months and an average follow-up time of 4 years. To the best of our knowledge, this is rarely seen in the existing medical literature. Besides, we also introduce an HD EMR dataset as an additional experimental dataset to test the prediction performance. In future work, we will extend AICare to multicenter healthcare systems and conduct a prospective multicenter controlled experiment to validate the framework in other clinical scenarios.

Incorporating more clinical features to depict health status

During the data collection process for this study, we collected many medical features of patients, most of which were discarded due to high missing rates. Our model only had access to the autofiltered 16 longitudinal medical features and 4 demographic features for each patient. The novelty of this research does not only lie in incremental model performance improvements. This predictive performance was achieved without hand selection or hand-made variables deemed important by a medical expert. AICare can achieve satisfactory prediction results and discover medical findings, proving the model's validity and practicability. In future releases, we expect to incorporate more medical features, such as medication records, dialysis adequacy records, complication records, and health data collected at home.

Providing recommendations for diverse patient cohorts

To obtain relatively stable and reasonable conclusions, the clinical recommendations in this paper are roughly generated by AICare for most PD patients. As more data are collected in the next release, we will provide refined recommendations for diverse patient cohorts (e.g., different genders and ages).

Embedding sequences with a relatively regular time interval

Our 1-year mortality risk prediction system, as developed in this paper, is primarily designed for scenarios involving outpatient follow-up of PD patients. It aligns with the approach of most relevant methods that deal with sequential EMRs using time-series-encoding models for prognosis prediction tasks, as illustrated by the methods listed in the Related work section in the supplemental information. Our network framework, AICare, is best suited for situations where there is a relatively regular interval between medical visits. For example.

(1) The majority of PD patients typically undergo routine outpatient lab tests at hospitals approximately every 2.5 months.
(2) In intensive care unit (ICU) settings, medical records for patients might be recorded on a daily basis or at even shorter intervals, as demonstrated in the sepsis prediction task in the supplemental information.

However, we acknowledge that some scenarios may involve significantly irregular records. In cases where raw data are input into our model without regularization, it could potentially result in a decline in prediction performance. To address this issue, it is crucial to preprocess the data using regularization techniques[48] or implement time-aware mechanisms to mitigate the adverse effects of irregular records.[49,50] Dealing with irregular records is indeed an important research branch within EMR analysis, but it is not the primary focus of this study.

Features with low importance weight

No significantly meaningful importance pattern was discovered for white blood cell count (WBC). This may be because WBC is not a crucial feature in mortality prediction or because WBC is such a special clinical feature that AICare does not know how to use it to embed the health representation. This reminds us to design proper embedding network modules (e.g., convolutional layers) to effectively utilize different feature effectively. Besides, considering that the proportion of immune cells may indicate the health status as a human-constructed advanced feature, we will introduce more related clinical features about the immune.

A robust prospective evaluation in future work

In this study, our focus is on application of our methodology to the 1-year mortality risk prediction task for PD patients and the interpretation of the results. Although it is widely believed that accurate predictions can be used to improve care,[51] this is not a foregone conclusion, and prospective trials are needed to demonstrate this.[52,53] We acknowledge that clinical practice is influenced by numerous factors, and while our observations provide some new medical insights, they should be considered preliminary and require further validation in larger prospective evaluations to establish efficacy and safety for patients. These observations, while serving as a reference and source of inspiration for subsequent research on EMR analysis, should not be immediately implemented in clinical practice without undergoing further validation. We will recruit patients and conduct a larger and more robust prospective evaluation in the future. We will

conduct a blinded application-grounded evaluation by inviting dozens of experienced medical practitioners (with 7–20 years of clinical practice time) from nephrology departments of different hospitals to test the practical effectiveness and degrees of physicians' recognition. The prototype version of the trial system with online questionnaires has already been developed and can be found at http://v.ai-care.top/table/questionnaire/a1. The AI-doctor interaction system is available in English and simplified Chinese. The questionnaire page is currently only available in simplified Chinese.

Conclusion

AICare is a generic framework proposed to model a patient's health status based on multivariate time-series EMR data. The AICare captures complex longitudinal progressions in patients' health conditions and provides dynamic predictions of mortality risk while also offering fine-grained interpretability to understand how the model arrives at specific predictions for individual patients. This interpretability aspect enables them to build trust in the model's predictions and better comprehend the reasoning behind the recommendations.

Our work also includes the development of an AI-doctor interaction system that leverages the capabilities of AICare to support clinicians in prognosis prediction. The system provides an intuitive visualization of the patient's health trajectory over time, allowing clinicians to track changes in key health indicators and identify trends in mortality risk predictions. Moreover, AICare offers personalized recommendations based on the model's predictions, suggesting target values for essential health indicators to improve the patient's survival outlook. These recommendations are instrumental in helping clinicians devise personalized treatment plans and monitoring strategies for their patients.

EXPERIMENTAL PROCEDURES

Resource availability

Lead contact

Yasha Wang is the lead contact of this study and can be reached via e-mail (wangyasha@pku.edu.cn).

Materials availability

This study did not generate new unique reagents.

Data and code availability

The code, training parameters, and training data are openly available on Zenodo (https://doi.org/10.5281/zenodo.10003570) and our website (http://v.ai-care.top/download). Users can instantly get predictions by uploading data to http://v.ai-care.top/predict or download the code for offline training with their dataset. Additional supporting data for this study can be requested from the corresponding author.

SUPPLEMENTAL INFORMATION

Supplemental information can be found online at https://doi.org/10.1016/j.patter.2023.100892.

ACKNOWLEDGMENTS

This work was supported by the National Natural Science Foundation of China (82241052). W.T. was supported by the Fund from Peking University Third Hospital (BYSYDL2023004) and the PKU-Baidu Foundation (2020BD030). L.M. was supported by the China Postdoctoral Science Foundation (2021TQ0011 and 2022M720237). X.Z. was supported by the Research Project of Blood Purification Center Branch of Chinese Hospital Association (CHABP2021-11). W.R. was supported by the UK EPSRC project on Offshore Robotics for Certification of Assets (ORCA) (EP/R026173/1). J.G. acknowledges the receipt of studentship awards from the Health Data Research UK-The Alan Turing Institute Wellcome PhD Program in Health Data Science (grant ref. 218529/Z/19/Z).

AUTHOR CONTRIBUTIONS

L.M. and C.Z. proposed the main architecture of the AICare model. The research process was designed by L.M., J.G., and Y.W.. C.Z., Y.Z., and X.J. conducted the experiment. L.M., Y.Z., Z.Y., X.M., and W.R. visualized the results. The AI-doctor interaction system was designed by L.M. and implemented by Y.Z., X.J., and Z.Y. The data were collected by T.W., W.T., and X.Z. J.G., L.M., and T.W. conducted the data pre-processing. The prediction task was formulated by L.M., J.G., W.T., and Y.W. W.T. and X.Z. extracted the medical findings.

DECLARATION OF INTERESTS

The authors declare no competing interests.

Received: April 25, 2023
Revised: August 18, 2023
Accepted: November 10, 2023
Published: December 8, 2023

REFERENCES

1. Teitelbaum, I. (2021). Peritoneal dialysis. N. Engl. J. Med. *385*, 1786–1795.
2. Morelle, J., Marechal, C., Yu, Z., Debaix, H., Corre, T., Lambie, M., Verduijn, M., Dekker, F., Bovy, P., Evenepoel, P., et al. (2021). Aqp1 promoter variant, water transport, and outcomes in peritoneal dialysis. N. Engl. J. Med. *385*, 1570–1580.
3. Bender, F.H., Bernardini, J., and Piraino, B. (2006). Prevention of infectious complications in peritoneal dialysis: best demonstrated practices. Kidney Int. *70*, S44–S54.
4. Xu, Z., Luo, Y., Adekkanattu, P., Ancker, J.S., Jiang, G., Kiefer, R.C., Pacheco, J.A., Rasmussen, L.V., Pathak, J., and Wang, F. (2019). Stratified mortality prediction of patients with acute kidney injury in critical care. In *MEDINFO 2019: Health and Wellbeing e-Networks for All* (462–466) (IOS Press), pp. 462–466.
5. Ravizza, S., Huschto, T., Adamov, A., Böhm, L., Büsser, A., Flöther, F.F., Hinzmann, R., König, H., McAhren, S.M., Robertson, D.H., et al. (2019). Predicting the early risk of chronic kidney disease in patients with diabetes using real-world data. Nat. Med. *25*, 57–59.
6. Chaudhuri, S., Han, H., Usvyat, L., Jiao, Y., Sweet, D., Vinson, A., Johnstone Steinberg, S., Maddux, D., Belmonte, K., Brzozowski, J., et al. (2021). Machine learning directed interventions associate with decreased hospitalization rates in hemodialysis patients. Int. J. Med. Inf. *153*, 104541.
7. Akbilgic, O., Obi, Y., Potukuchi, P.K., Karabayir, I., Nguyen, D.V., Soohoo, M., Streja, E., Molnar, M.Z., Rhee, C.M., Kalantar-Zadeh, K., and Kovesdy, C.P. (2019). Machine learning to identify dialysis patients at high death risk. Kidney Int. Rep. *4*, 1219–1229.
8. Liu, J., Wu, J., Liu, S., Li, M., Hu, K., and Li, K. (2021). Predicting mortality of patients with acute kidney injury in the icu using xgboost model. PLoS One *16*, e0246306.
9. Zhou, Q., You, X., Dong, H., Lin, Z., Shi, Y., Su, Z., Shao, R., Chen, C., and Zhang, J. (2021). Prediction of premature all-cause mortality in patients receiving peritoneal dialysis using modified artificial neural networks. Aging (Albany NY) *13*, 14170–14184.
10. Radović, N., Prelević, V., Erceg, M., and Antunović, T. (2022). Machine learning approach in mortality rate prediction for hemodialysis patients. Comput. Methods Biomech. Biomed. Eng. *25*, 111–122.

CellPress
OPEN ACCESS

11. Kang, M.W., Kim, J., Kim, D.K., Oh, K.-H., Joo, K.W., Kim, Y.S., and Han, S.S. (2020). Machine learning algorithm to predict mortality in patients undergoing continuous renal replacement therapy. Crit. Care *24*, 42–49.

12. Noh, J., Yoo, K.D., Bae, W., Lee, J.S., Kim, K., Cho, J.-H., Lee, H., Kim, D.K., Lim, C.S., Kang, S.-W., et al. (2020). Prediction of the mortality risk in peritoneal dialysis patients using machine learning models: a nation-wide prospective cohort in korea. Sci. Rep. *10*, 7470.

13. Schena, F.P., Anelli, V.W., Trotta, J., Di Noia, T., Manno, C., Tripepi, G., D'Arrigo, G., Chesnaye, N.C., Russo, M.L., Stangou, M., et al. (2021). Development and testing of an artificial intelligence tool for predicting end-stage kidney disease in patients with immunoglobulin a nephropathy. Kidney Int. *99*, 1179–1188.

14. Rank, N., Pfahringer, B., Kempfert, J., Stamm, C., Kühne, T., Schoenrath, F., Falk, V., Eickhoff, C., and Meyer, A. (2020). Deep-learning-based real-time prediction of acute kidney injury outperforms human predictive performance. NPJ Digit. Med. *3*, 139.

15. Tomašev, N., Glorot, X., Rae, J.W., Zielinski, M., Askham, H., Saraiva, A., Mottram, A., Meyer, C., Ravuri, S., Protsyuk, I., et al. (2019). A clinically applicable approach to continuous prediction of future acute kidney injury. Nature *572*, 116–119.

16. Srinivas, T.R., Taber, D.J., Su, Z., Zhang, J., Mour, G., Northrup, D., Tripathi, A., Marsden, J.E., Moran, W.P., and Mauldin, P.D. (2017). Big data, predictive analytics, and quality improvement in kidney transplantation: a proof of concept. Am. J. Transplant. *17*, 671–681.

17. Yan, L., Zhang, H.-T., Goncalves, J., Xiao, Y., Wang, M., Guo, Y., Sun, C., Tang, X., Jing, L., Zhang, M., et al. (2020). An interpretable mortality prediction model for covid-19 patients. Nat. Mach. Intell. *2*, 283–288.

18. Hyland, S.L., Faltys, M., Hüser, M., Lyu, X., Gumbsch, T., Esteban, C., Bock, C., Horn, M., Moor, M., Rieck, B., et al. (2020). Early prediction of circulatory failure in the intensive care unit using machine learning. Nat. Med. *26*, 364–373.

19. Thorsen-Meyer, H.-C., Nielsen, A.B., Nielsen, A.P., Kaas-Hansen, B.S., Toft, P., Schierbeck, J., Strøm, T., Chmura, P.J., Heimann, M., Dybdahl, L., et al. (2020). Dynamic and explainable machine learning prediction of mortality in patients in the intensive care unit: a retrospective study of high-frequency data in electronic patient records. Lancet. Digit. Health *2*, e179–e191.

20. Makino, M., Yoshimoto, R., Ono, M., Itoko, T., Katsuki, T., Koseki, A., Kudo, M., Haida, K., Kuroda, J., Yanagiya, R., et al. (2019). Artificial intelligence predicts the progression of diabetic kidney disease using big data machine learning. Sci. Rep. *9*, 11862–11869.

21. Alvarez Melis, D., and Jaakkola, T. (2018). Towards robust interpretability with self-explaining neural networks. Adv. Neural Inf. Process. Syst. *31*.

22. Meyer, A., Zverinski, D., Pfahringer, B., Kempfert, J., Kuehne, T., Sündermann, S.H., Stamm, C., Hofmann, T., Falk, V., and Eickhoff, C. (2018). Machine learning for real-time prediction of complications in critical care: a retrospective study. Lancet Respir. Med. *6*, 905–914.

23. Nitski, O., Azhie, A., Qazi-Arisar, F.A., Wang, X., Ma, S., Lilly, L., Watt, K.D., Levitsky, J., Asrani, S.K., Lee, D.S., et al. (2021). Long-term mortality risk stratification of liver transplant recipients: real-time application of deep learning algorithms on longitudinal data. Lancet. Digit. Health *3*, e295–e305.

24. Sung, M., Hahn, S., Han, C.H., Lee, J.M., Lee, J., Yoo, J., Heo, J., Kim, Y.S., and Chung, K.S. (2021). Event prediction model considering time and input error using electronic medical records in the intensive care unit: Retrospective study. JMIR Med. Inform. *9*, e26426.

25. Tsirpanlis, G., Bagos, P., Ioannou, D., Bleta, A., Marinou, I., Lagouranis, A., Chatzipanagiotou, S., Nicolaou, C., do Nascimento, M.M., Stenvinkel, P., et al. (2005). Serum albumin: a late-reacting negative acute-phase protein in clinically evident inflammation in dialysis patients. Nephrol. Dial. Transplant. *20*, 658–659.

26. de Mutsert, R., Grootendorst, D.C., Indemans, F., Boeschoten, E.W., Krediet, R.T., and Dekker, F.W.; Netherlands Cooperative Study on the Adequacy of Dialysis-II Study Group (2009). Association between serum albumin and mortality in dialysis patients is partly explained by inflammation, and not by malnutrition. J. Ren. Nutr. *19*, 127–135.

27. Li, P.K.-T., Szeto, C.C., Piraino, B., de Arteaga, J., Fan, S., Figueiredo, A.E., Fish, D.N., Goffin, E., Kim, Y.-L., Salzer, W., et al. (2016). Ispd peritonitis recommendations: 2016 update on prevention and treatment. Perit. Dial. Int. *36*, 481–508.

28. Jeerakathil, T.J., and Wolf, P.A. (2003). Epidemiology and stroke risk factors. In *Office Practice of Neurology* (252–268) (Elsevier), pp. 252–268.

29. O'Rourke, M.F., Hartley, C., and McDonald, D.A. (1998). McDonald's Blood Flow in Arteries: Theoretic, Experimental, and Clinical Principles (Arnold).

30. Fang, J., Madhavan, S., Cohen, H., and Alderman, M.H. (1995). Measures of blood pressure and myocardial infarction in treated hypertensive patients. J. Hypertens. *13*, 413–419.

31. Webb, A.J.S. (2020). Progression of arterial stiffness is associated with midlife diastolic blood pressure and transition to late-life hypertensive phenotypes. J. Am. Heart Assoc. *9*, e014547.

32. Zager, P.G., and Rohrscheib, M.R. (2009). Blood pressure and mortality risk in patients treated by peritoneal dialysis. Am. J. Kidney Dis. *53*, 9–11.

33. Basol, M., Goksuluk, D., Sipahioglu, M.H., and Karaagaoglu, E. (2021). Effect of serum albumin changes on mortality in patients with peritoneal dialysis: A joint modeling approach and personalized dynamic risk predictions. BioMed Res. Int. *2021*, 6612464.

34. Udayaraj, U.P., Steenkamp, R., Caskey, F.J., Rogers, C., Nitsch, D., Ansell, D., and Tomson, C.R.V. (2009). Blood pressure and mortality risk on peritoneal dialysis. Am. J. Kidney Dis. *53*, 70–78.

35. Xie, X., Lv, D., Zheng, H., Zhang, X., Han, F., and Chen, J. (2020). The associations of blood pressure parameters with all-cause and cardiovascular mortality in peritoneal dialysis patients: a cohort study in china. J. Hypertens. *38*, 2252–2260.

36. Hannedouche, T., Roth, H., Krummel, T., London, G.M., Jean, G., Bouchet, J.-L., Drüeke, T.B., and Fouque, D.; French Observatory (2016). Multiphasic effects of blood pressure on survival in hemodialysis patients. Kidney Int. *90*, 674–684.

37. Robinson, B.M., Tong, L., Zhang, J., Wolfe, R.A., Goodkin, D.A., Greenwood, R.N., Kerr, P.G., Morgenstern, H., Li, Y., Pisoni, R.L., et al. (2012). Blood pressure levels and mortality risk among hemodialysis patients in the dialysis outcomes and practice patterns study. Kidney Int. *82*, 570–580.

38. Navaneethan, S.D., Schold, J.D., Jolly, S.E., Arrigain, S., Blum, M.F., Winkelmayer, W.C., and Nally, J.V., Jr. (2017). Blood pressure parameters are associated with all-cause and cause-specific mortality in chronic kidney disease. Kidney Int. *92*, 1272–1281.

39. Afshinnia, F., Zaky, Z.S., Metireddy, M., and Segal, J.H. (2016). Reverse epidemiology of blood pressure in peritoneal dialysis associated with dynamic deterioration of left ventricular function. Perit. Dial. Int. *36*, 154–162.

40. Flythe, J.E., Chang, T.I., Gallagher, M.P., Lindley, E., Madero, M., Sarafidis, P.A., Unruh, M.L., Wang, A.Y.-M., Weiner, D.E., Cheung, M., et al. (2020). Blood pressure and volume management in dialysis: conclusions from a kidney disease: Improving global outcomes (kdigo) controversies conference. Kidney Int. *97*, 861–876.

41. Park, J., Mehrotra, R., Rhee, C.M., Molnar, M.Z., Lukowsky, L.R., Patel, S.S., Nissenson, A.R., Kopple, J.D., Kovesdy, C.P., and Kalantar-Zadeh, K. (2013). Serum creatinine level, a surrogate of muscle mass, predicts mortality in peritoneal dialysis patients. Nephrol. Dial. Transplant. *28*, 2146–2155.

42. Avram, M.M., Mittman, N., Bonomini, L., Chattopadhyay, J., and Fein, P. (1995). Markers for survival in dialysis: a seven-year prospective study. Am. J. Kidney Dis. *26*, 209–219.

43. Kuo, K.-L., Hung, S.-C., Tseng, W.-C., Tsai, M.-T., Liu, J.-S., Lin, M.-H., Hsu, C.-C., and Tarng, D.-C.; Taiwan Society of Nephrology Renal Registry Data System (2018). Association of anemia and iron parameters with mortality among patients undergoing prevalent hemodialysis in Taiwan: The AIM-HD study. J. Am. Heart Assoc. *7*, e009206.

44. Molnar, M.Z., Mehrotra, R., Duong, U., Kovesdy, C.P., and Kalantar-Zadeh, K. (2011). Association of hemoglobin and survival in peritoneal dialysis patients. Clin. J. Am. Soc. Nephrol. *6*, 1973–1981.

45. Kliger, A.S., Foley, R.N., Goldfarb, D.S., Goldstein, S.L., Johansen, K., Singh, A., and Szczech, L. (2013). Kdoqi us commentary on the 2012 kdigo clinical practice guideline for anemia in ckd. Am. J. Kidney Dis. *62*, 849–859.

46. Cheng, L.-T., Tang, W., and Wang, T. (2005). Strong association between volume status and nutritional status in peritoneal dialysis patients. Am. J. Kidney Dis. *45*, 891–902.

47. Wikipedia (2019). Cerebrovascular disease. https://en.wikipedia.org/w/index.php?title=Cerebrovascular_disease&oldid=876465095. (Accessed 3 February 2019). accessed.

48. Zheng, K., Gao, J., Ngiam, K.Y., Ooi, B.C., and Yip, W.L.J. (2017a). Resolving the bias in electronic medical records. In Proceedings of the 23rd ACM SIGKDD International Conference on Knowledge Discovery and Data Mining (ACM), pp. 2171–2180.

49. Baytas, I.M., Xiao, C., Zhang, X., Wang, F., Jain, A.K., and Zhou, J. (2017). Patient subtyping via time-aware lstm networks. In Proceedings of the 23rd ACM SIGKDD International Conference on Knowledge Discovery and Data Mining (ACM). 65–74.

50. Zheng, K., Wang, W., Gao, J., Ngiam, K.Y., Ooi, B.C., and Yip, W.L.J. (2017b). Capturing feature-level irregularity in disease progression modeling. In Proceedings of the 2017 ACM on Conference on Information and Knowledge Management, pp. 1579–1588.

51. Bates, D.W., Saria, S., Ohno-Machado, L., Shah, A., and Escobar, G. (2014). Big data in health care: using analytics to identify and manage high-risk and high-cost patients. Health Aff. *33*, 1123–1131.

52. Krumholz, H.M., Terry, S.F., and Waldstreicher, J. (2016). Data acquisition, curation, and use for a continuously learning health system. JAMA *316*, 1669–1670.

53. Grumbach, K., Lucey, C.R., and Johnston, S.C. (2014). Transforming from centers of learning to learning health systems: the challenge for academic health centers. JAMA *311*, 1109–1110.

Patterns, Volume *4*

Supplemental information

Mortality prediction with adaptive feature importance recalibration for peritoneal dialysis patients

Liantao Ma, Chaohe Zhang, Junyi Gao, Xianfeng Jiao, Zhihao Yu, Yinghao Zhu, Tianlong Wang, Xinyu Ma, Yasha Wang, Wen Tang, Xinju Zhao, Wenjie Ruan, and Tao Wang

Appendix for Mortality Prediction with Adaptive Feature Importance Recalibration for Peritoneal Dialysis Patients

Materials and Method Details

Dataset

This work includes 13,091 visits of 656 end-stage renal disease peritoneal dialysis patients from the Department of Nephrology of a large grade A tertiary (the highest level in the nation's three-tier grading system for hospitals). Fig. s1 shows the distribution of age and visit frequency. The average age of patients at the first clinical visit is 58.55 years old, with a standard deviation (Std) of 15.81 years. The average visiting frequency of patients at the end of the clinical follow-up was 19.95, with an Std of 13.53.

Problem Formulation

We formulate the model inputs and prediction tasks as follows:

Definition 1 (**Patient Records**). A patient's visit records can be represented as a matrix $\mathbf{R} \in \mathbb{R}^{N\times T}$, where N denotes the number of medical features in visit records and T denotes the number of visits within the observation window. We use vector $\mathbf{r}_{n,t}$ to denote the t-th visit of the n-th feature. The baseline information is denoted as vector $\mathbf{r}_0$.

Problem 1 (**One-year Mortality Prediction**). Given a patient's visit records $\mathbf{R}$ and baseline information $\mathbf{r}_0$, our objective is to predict the mortality risk $\widehat{\mathbf{y}}_t$ in the next year for the patient at each visit. This is formulated as a binary classification task as $\mathbf{y} \in \{0, 1\}$.

Considering the uncertainty of the health status variation in the observation window, we design a particular labeling strategy to make the training labels as close to the ground truth as possible. As shown in Fig. s2, for patients with positive labels (i.e., mortality) at the end of clinical follow-up, we consider all visits within one year before the mortality date as high-risk visits ($y = 1$). For patients with negative labels at the end, we consider all visits within one year before the last visit as *uncertain*, since we do not know whether the patient will have an adverse outcome in the next year ($y = uncertain$). The calculation of loss function and performance metrics will not include these visits. Other visits are all labeled as low-risk ($y = 0$).

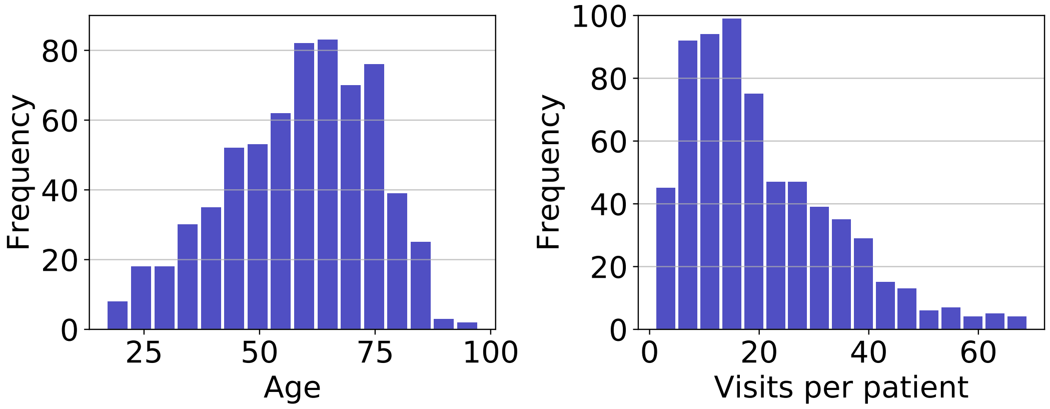

Figure s1: **Distribution of Age and Visit Frequency in PD Dataset.**

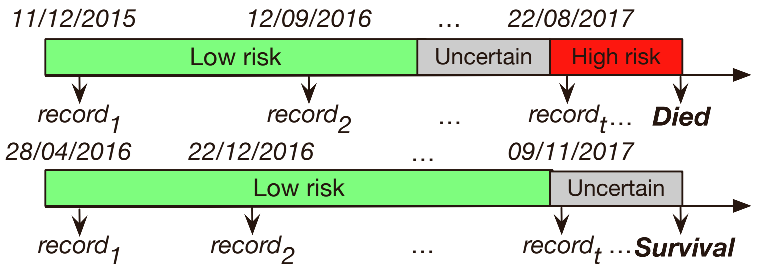

Figure s2: **Label Assignment.** The prediction task is defined as a 1-year mortality prediction at each clinical visit. Clinical visits within 1 year before death are labeled as *high risk* $(y = 1)$. Visits recorded 2 years before death are labeled as *low risk* $(y = 1)$. Visits recorded between 1 and 2 years before death are labeled as *uncertain* status and will not be included in the training process.

Problem 2 (**Model Interpretability**). For a given patient, the model will output an importance score matrix α, where the value $\alpha_{n,t}$ in the n-th row and t-th column denotes the importance score for feature n at visit t. This importance score represents how much the feature contributes to the prediction.

Model Detail

We propose a general healthcare predictive model, which can adaptively depict patients' health status in diverse conditions and provide reasonable interpretability. The model explicitly captures the interdependencies among time series of dynamic features and static baseline information to learn the personal health context of patients in a global view. As shown in Fig.1-(2), `AICare` comprises the following sub-modules:

- The multi-channel feature extraction module is developed to learn the representation of each dynamic feature separately.
- The adaptive feature importance recalibration module enhances the key factors explicitly by squeeze-and-excitation block to perform the individualized clinical prediction.

Multi-Channel Feature Extraction

The patient's health status is depicted by dynamic feature sequences and static baseline features. To embed such heterogeneous information and build the final health representation, as well as make the dynamic feature's importance assignment process more intuitive, we develop a multi-channel feature extraction module.

Considering that the logical time order of the clinical sequence matters in the medical domain, `AICare` embeds the time series of each dynamic feature separately by multi-channel bidirectional GRUs. The GRUs at the bottom level will refine the representation of each feature and form a sequence of $N + 1$ feature vectors (N for dynamic record features $\mathbf{R}$ and 1 for the baseline information $\mathbf{r}_0$). Specifically, `AICare` embeds the time series of each feature separately by multi-channel bidirectional GRU:

$$(\overrightarrow{\mathbf{f}_{n,1}}, ..., \overrightarrow{\mathbf{f}_{n,T}}), (\overleftarrow{\mathbf{f}_{n,1}}, ..., \overleftarrow{\mathbf{f}_{n,T}}) = \text{bi-GRU}_n(\mathbf{r}_{n,1}, ..., \mathbf{r}_{n,T}), \tag{1}$$

where the time series of feature n is denoted as $\mathbf{r}_{n,:} = (\mathbf{r}_{n,1}, ..., \mathbf{r}_{n,T}) \in R^T$. We derive each feature representation as the sum of the two last hidden embeddings:

$$\mathbf{f}_n = \overrightarrow{\mathbf{f}_{n,T}} + \overleftarrow{\mathbf{f}_{n,1}}. \tag{2}$$

Furthermore, the demographic baseline data ($\mathbf{r}_0$) is embedded as:

$$\mathbf{f}_0 = W_0^{emb} \cdot \mathbf{r}_0, \tag{3}$$

where W_0^{emb} is the embedding matrix. From here on, we ignore the bias term for ease of notation. Thus, all the patient data can be represented by a matrix $\mathbf{F}$ (i.e., a sequence of vectors, where each vector represents one feature of the patient over time): $\mathbf{F} = (\mathbf{f}_0, \mathbf{f}_1, \cdots, \mathbf{f}_N)$.

Adaptive Feature Importance Recalibration

We develop an adaptive feature importance recalibration module to provide the feature's importance weight when performing mortality prediction at each clinical visit. This attention-based module is inspired by the $SEblock$ in the research area of computer vision[1], and is trained to explicitly model the dependencies between clinical features. It can selectively give more weight to the representative and predictive features but suppresses unimportant ones.

First, to select the most informative features, we should provide the model with a global view of patients' status at the current visit. For a particular patient, `AICare` squeezes all the feature embedding via a mean pooling operation to get an integrated but straightforward picture of the health status. It can be regarded as an abstract of the patient's all historical status. The importance of different temporal patterns will be calculated based on this abstract. Second, we selectively give more weight to the predictive features but suppress unimportant ones, which contribute little to the prediction target. The selectively enhanced predictive features can be treated as a precursor of health risk for the given patient.

Concretely, the $Query$ is obtained by $\mathbf{f}_{sqz}$ from embedded health information $\mathbf{F}$, including dynamic features and baseline features. The Keys are formed by embedded dynamic features $\mathbf{f}_1, \cdots, \mathbf{f}_N$ as:

$$\mathbf{f}_{sqz} = \text{Mean\_pooling}(\mathbf{f}_0, \mathbf{f}_1, \cdots, \mathbf{f}_N), \tag{4}$$

$$\mathbf{q}_{sqz}^{fin} = W_{sqz}^{fin} \cdot \mathbf{f}_{sqz}, \tag{5}$$

$$\mathbf{k}_n^{fin} = W_n^{fin} \cdot \mathbf{f}_n, \quad (n = 1, ..., N), \tag{6}$$

where W_{sqz}^{fin} and W_n^{fin} are the projection matrix, respectively. Then the attention weights are calculated as follows:

$$\alpha_1^{fin}, ..., \alpha_N^{fin} = \delta(\zeta_1^{fin}, ..., \zeta_N^{fin}), \tag{7}$$

$$\zeta_n^{fin} = \mathbf{q}_{sqz}^{fin} \cdot \mathbf{k}_n^{fin} \quad (n = 1, ..., N), \tag{8}$$

where δ denotes the activation function (i.e., *softmax* or *sparsemax*). *Sparsemax* activation will make the model interpretability more prominent by allowing the most critical features to dominate the final embedding. Using *sparsemax* activation will slightly weaken the performance since it suppresses too much information from features that are not most important. In this work, we employ *softmax* function to obtain the prediction results, and employ *sparsemax* function to extract medical findings. Finally, the health status representation s and the prediction result $\widehat{\mathbf{y}}$ can be obtained by:

$$\mathbf{s} = \text{Concat}[\sum_{n=1}^{N} \alpha_n^{fin} \cdot \mathbf{f}_n^*, \ \mathbf{f}_0^*], \tag{9}$$

$$\widehat{\mathbf{y}} = \text{Sigmoid}(W^{final} \cdot \mathbf{s}), \tag{10}$$

where W^{final} is the weight matrix.

Experiment Details

Experiment Setup

Implementation Details

The training is done in a machine equipped with CPU: Intel Xeon E5-2630, 256GB RAM, and GPU: Nvidia Titan V using Pytorch 1.1.0. We use Adam[2] with the mini-batch of 256 patients, and the learning rate is set to $1e-3$. To fairly compare different approaches, the hyper-parameters of the baseline models are fine-tuned by a grid-searching strategy.

Baseline Approaches

We implement several state-of-the-art (SOTA) representative models as comparative baseline approaches. The hyper-parameters of models are fine-tuned by a grid-searching strategy.

GRU: Gated Recurrent Unit neural network embeds the time series to perform the target prediction. It is a widely applied variant of the Recurrent Neural Network (RNN), which improves the capability to maintain historical memories and reduces parameters in the update and reset gates. GRU has been used to predict several severe complications (mortality, renal failure with a need for renal replacement therapy, and postoperative bleeding leading to operative revision) in post-cardiosurgical care in real-time (Lancet Respiratory Medicine, 2018)[3].

Transformer$_e$ is the encoder of the Transformer[4], which comprises the positional encoding module and the self-attention module. Transformer has been used to perform the mortality risk analysis for liver transplant recipients[5].

MT-RHN: Multi-Task Deep Recurrent Highway Network, which embeds the historical data and current step with a deep residual embedding component, and employs a multi-task learning strategy to enhance the performance. MT-RHN has been used to perform the continuous risk prediction of future acute kidney injury deterioration occurring in the next 48 hours (Nature, 2019)[6].

LSTM: Long Short Term Memory network is a variant of the Recurrent Neural Network (RNN), capable of learning long-term dependencies. LSTM has been used to perform the 90-day all-cause mortality in the intensive care unit (ICU), based on the concatenated static features and dynamic features (Lancet Digital Health, 2020)[7].

biLSTM-FC: Bidirectional LSTM with Fully Connected layers. Bidirectional LSTM (biLSTM) is a sequence processing model that consists of two LSTMs: one taking the input in a forward direction and the other in a backward direction. Sung et al. have used biLSTM-FC to perform the clinical event prediction (death, sepsis, and acute kidney injury), where biLSTM and fully connected layers are employed to embed the dynamic features and static features correspondingly (JMIR, 2021)[8].

XGBoost: a recursive tree-based supervised machine learning classifier. XGBoost has been used to predict the mortality for COVID-19 infected patients (Nature Machine Intelligence, 2020)[9].

DT: Decision Tree, a non-parametric supervised learning algorithm with a hierarchical tree structure. DT has been used to perform mortality prediction for peritoneal dialysis patients (Nature Scientific Reports, 2020)[10].

LightGBM: a gradient boosting ensemble framework that uses tree-based learning algorithms. LightGBM has been used to perform the early prediction of circulatory failure in the intensive care unit (Nature Medicine, 2020)[11].

LR: Logistic Regression model predicts a dependent data variable by analyzing the relationship between one or more existing independent variables. LR has been used to predict the early risk of chronic kidney disease in patients with diabetes (Nature Medicine, 2019)[12].

Evaluation Metrics

We evaluate the models with a 10-fold cross-validation strategy and report the average performance, similar to[13]. We assess performance for the binary classification problem using the area under the receiver operating characteristic curve (AUROC) and the area under the precision-recall curve (AUPRC). AUPRC is the most informative and the primary evaluation metric when dealing with a highly imbalanced and skewed dataset[14,15] like the real-world EMR data.

Hyper-Parameter Settings

Specially, the hyper-parameter setting of the proposed `AICare` is as follows: We set the embedding dimension and hidden dimension as 16 / 16 / 32 for PD Patients / Hemodialysis ESRD / Challenge dataset, respectively. We use Adam optimization algorithm with the batch size of 256 and the learning rate is set to $1e-3$. We implement several state-of-the-art models as our baseline approaches. For the hyper-parameter settings of our baseline models, our principle is as follows: If the hyper-parameter setting is available in the original paper, we will use the recommended setting. Otherwise, the hyper-parameters of the baseline models are fine-tuned by the grid-searching strategy.

GRU/LSTM/biLSTM-FC/Transformer/MT-RHN The hidden units are set to 16 / 16 / 32 for PD Patients / Hemodialysis ESRD / Challenge dataset, respectively, and the dropout rate is 0.5. **LR**: the number of max iterations is set to 200. **XGBoost**: the max depth is set to 5, with 50 estimators and 0.1 learning rate. **DT**: the max depth is set to 5. **LightGBM**: the max depth is set to 5, with 50 estimators and 0.01 learning rate.

Prediction Performance of `AICare` for Different Causes of Death on PD Dataset

There are nine different CODs recorded: Cerebrovascular Disease (CVE), Cardiovascular Disease (CVD), Peritoneal Dialysis Associated Peritonitis (PDAP, Peritonitis), Peripheral Vascular Disease (PVD), Infections, Gastrointestinal Disease (GI Disease), Cachexia, Cancer and Other causes. We evaluate the performance for patients with different causes of death (COD). Since our experiment was conducted via 10-fold cross-validation, we employ the model trained on each fold's training set to the corresponding testset to perform the prediction.

The statistics of patients with different mortality causes are shown in Table s1. The receiver operating characteristic (ROC) curves for different COD patient subgroups are shown in Fig. s3. According to the prediction results, the risk of cachaxia (AUROC = 0.88), infection (AUROC = 0.82) and PVD (AUROC = 0.82) are easy to be identified. `AICare` provides accurate prediction results of these patients about a year before the outcome.

On the contrary, patients with CVE (AUROC = 0.55) and CVD (AUROC = 0.71) are the most difficult to predict by the model. These diseases often attack untimely and acutely without obvious signs[16], compared to cachexia, infections, and PVD. Patients with these health risk factors

Table s1: **Statistics of patients with different mortality causes.** This real-world dataset contains 656 peritoneal dialysis (PD) patients. 261 (39.8%) patients of them, unfortunately, died before the final follow-up. There are nine different causes of death (COD) recorded.

Causes of Death	# Patients (%)	AUROC
Cerebrovascular Disease (CVE)	74 (28.3%)	0.55
Cardiovascular Disease (CVD)	21 (8.0%)	0.71
Gastrointestinal Disease (GI Disease)	17 (6.5%)	0.73
Peritoneal Dialysis Associated Peritonitis (PDAP)	21 (8.0%)	0.74
Cancer	23 (8.8%)	0.76
Other	50 (19.1%)	0.80
Infection	33 (12.6%)	0.82
Peripheral Vascular Disease (PVD)	13 (4.9%)	0.82
Cachexia	9 (3.4%)	0.88
Mortality	261 (100.0%)	-

may have a higher threat and probability of sudden death in quite a short period of time, which is hard to take early warnings.

Additional Experiments on External Public Datasets

`AICare` is a generic framework proposed to model the patient's health status on multi-variate time series EMR data. The analysis of the peritoneal dialysis dataset in this paper serves as a proof of concept. To verify the generalizability of `AICare`, we train the model to perform the prognosis prediction tasks on external real-world public datasets (i.e., mortality prediction on the DOPPS hemodialysis ESRD dataset, sepsis prediction on the PhysioNet Challange Dataset).

Mortality Prediction on the DOPPS Hemodialysis ESRD Dataset

We perform the 1-year mortality prediction on the DOPPS hemodialysis ESRD dataset[17] [1]. The statistics of the dataset are listed in Table s2. The results in Table s4 indicate that `AICare` also achieves better performance than the baseline models. We notice that XGBoost achieves a higher AUPRC while a lower AUROC. This is because AUPRC is often more informative than AUROC when dealing with highly imbalanced datasets. In a scenario where one class significantly outnumbers the other, a random classifier could achieve an AUROC of 0.5, but the AUPRC might be higher or lower depending on the underlying distribution of the data and the way the model is handling the classes. So both XGBoost and GRU might be focusing on aspects that lead to higher precision and recall for the positive class, hence the higher AUPRC, while XGBoost fails to distinguish between classes in general, reflected in the AUROC score.

Sepsis Prediction on the PhysioNet Challenge Dataset

We perform the sepsis prediction on an open-source challenge dataset[18] [2]. The sepsis prediction data is from three geographically distinct U.S. hospital systems with three different electronic medical record systems. These data were collected over the past decade with approval from

[1] The DOPPS hemodialysis ESRD dataset is made available directly through an international platform `https://www.dopps.org/OurStudies/HemodialysisDOPPS.aspx`, and it can be obtained from DOPPS research community website:`https://www.dopps.org/PartnerwithUs.aspx`.

[2] The Physionet Challenge dataset is available at `https://physionet.org/content/challenge-2019/`

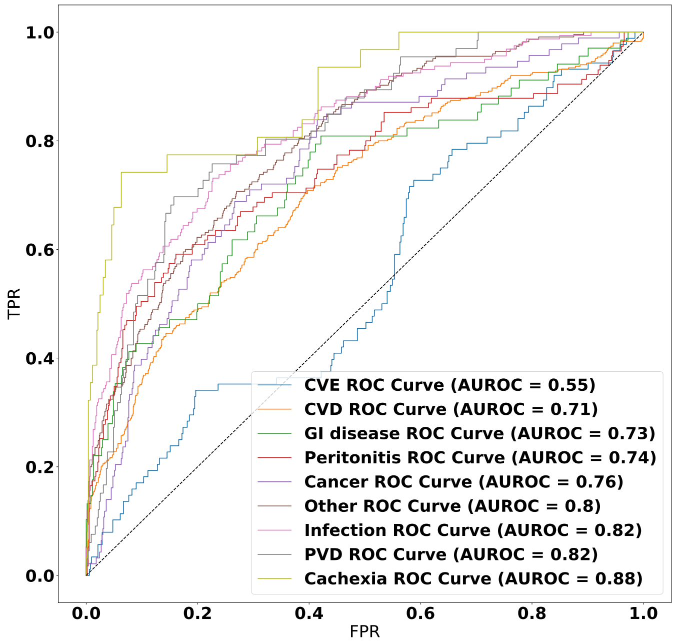

Figure s3: **Prediction ROC Results of Different Mortality Causes.** Cerebrovascular Disease (CVE) and Cardiovascular Disease (CVD) are the most challenging mortality causes to predict. On the contrary, Infections, Peripheral Vascular Disease (PVD), and Cachexia risks are relatively easy to be early identified.

Table s2: **Statistics of Hemodialysis Patient EMR as an Additional Dataset.** The real-world dataset contains 1,363 hemodialysis dialysis (HD) patients with 4,789 clinical visits. There are 12.55% patients, unfortunately, who died before the final follow-up. The average age of patients enrolled is 59 years old.

	Total	Survival (%)	Mortality (%)
# Patients	1363	1192 (87.45%)	171(12.55%)
# Visits	4789	4359 (91.02%)	430 (8.98%)
Avg. Age	59.38	57.89	68.67

Table s3: **Feature Summary of Hemodialysis (HD) Dataset.** This dataset comprises 18 dynamic features recorded at each clinical visit and 4 static baseline features recorded at the first visit.

Abbrev.	Full Name	Unit	Low Risk Visits ($y = 0$)			High Risk Visits ($y = 1$)			% Missing
Dynamic Features			Mean	Std	Median	Mean	Std	Median	
CO_2CP	CO_2 Combining Power	mmol/L	21.579	3.867	21.6	21.292	4.066	21.4	27%
WBC	White Blood Cell Count	x10^9/L	6.216	1.981	5.950	6.254	2.391	5.85	6%
Hb	Hemoglobin	g/L	107.9	17.283	109.5	103.6	18.66	105	6%
Ca	Calcium	mmol/L	2.272	0.24	2.258	2.253	0.236	2.24	41%
K	Potassium	mmol/L	5.045	0.818	5	4.851	0.851	4.8	13%
Na	Sodium	mmol/L	139.1	3.451	139	138.6	3.437	138.9	16%
Cr	Creatinine	umol/L	933.4	296.7	917.8	784.6	306.0	740.3	17%
P	Phosphorus	mmol/L	1.896	0.636	1.811	1.817	0.671	1.701	15%
Albumin	Albumin	g/L	39.49	4.212	39.9	36.94	4.838	37.5	27%
Glucose	Glucose	mmol/L	7.131	3.677	6.11	7.846	4.361	6.68	38%
pre-Weight	Pre-Dialysis Weight	kg	62.35	12.24	61.5	60.57	11.91	60.01	61%
pst-Weight	Post-Dialysis Weight	kg	59.94	11.96	59.06	58.23	11.58	57.23	62%
pre-SBP	Pre-Dialysis SBP	mmHg	147.3	20.58	147	146.7	20.96	148.6	61%
pst-SBP	Post-Dialysis SBP	mmHg	137.9	22.02	137.6	137.9	22.52	138.6	61%
pre-DBP	Pre-Dialysis DBP	mmHg	78.88	11.9	78.66	75.62	11.83	76	61%
pst-DBP	Post-Dialysis DBP	mmHg	77.44	12.05	77	73.62	12.53	73.33	61%
pre-Urea	Pre-Dialysis Urea	mmol/L	45.51	19.65	39.9	43.15	20.24	36.76	61%
pst-Urea	Post-Dialysis Urea	mmol/L	15.11	8.526	12.88	15.02	8.967	12.79	67%
Baseline Features									
BMI	Body Mass Index	-	21.87	3.633	21.39	21.83	4.081	21.60	57%
Gender	Female (0) or male (1)	-	0.54	0.498	1	0.557	0.497	1	0%
Age	Age	year	58.19	14.21	59	67.75	12.72	70	0%
Diabetes	Is (1) or not (0) has diabetes	-	0.267	0.442	0	0.351	0.478	0	0%

Table s4: **Mortality Prediction Performance on Hemodialysis ESRD Dataset.** Our proposed deep-learning-based model, `AICare`, outperforms other SOTA baseline comparative approaches.

Method	AUPRC	AUROC
GRU[3]	0.252(0.086)	0.702(0.083)
Transformer[5]	0.256(0.096)	0.695(0.100)
MT-RHN[6]	0.275 (0.089)	0.735 (0.080)
LSTM[7]	0.257 (0.085)	0.714 (0.074)
biLSTM-FC[8]	0.287 (0.082)	0.731 (0.078)
LR[12]	0.166 (0.076)	0.522 (0.031)
XGBoost[9]	0.222 (0.118)	0.518 (0.017)
DT[10]	0.202 (0.034)	0.539 (0.023)
LightGBM[11]	0.168 (0.111)	0.514 (0.020)
`AICare`	**0.325**** (0.122)	**0.743**** (0.088)

Table s5: **Results of the Sepsis Prediction on Challenge Dataset.**

Methods	AUPRC	AUROC
GRU[3]	0.7016 (0.026)	0.9352 (0.007)
Transformer[5]	0.6237 (0.031)	0.8946 (0.012)
MT-RHN[6]	0.2016 (0.019)	0.7378 (0.015)
LSTM[7]	0.7173 (0.025)	0.9351 (0.007)
biLSTM-FC[8]	0.7070 (0.026)	0.9303 (0.008)
LR[12]	0.0755 (0.004)	0.5040 (0.002)
XGBoost[9]	0.2986 (0.025)	0.8212 (0.012)
DT[10]	0.1924 (0.021)	0.6513 (0.016)
LightGBM[11]	0.2777 (0.025)	0.7972 (0.013)
`AICare`	**0.7731** (0.023)	.**0.9527** (0.019)

the appropriate Institutional Review Boards. They are labeled by Sepsis-3 clinical criteria. The cleaned dataset consists of 40,336 patients and consists of hourly vital sign summaries, lab values, and static patient descriptions. In particular, the data contained 40 clinical variables: 8 vital sign variables, 26 laboratory variables, and 6 demographic variables. We fix a test set of 10% of patients and divide the rest of the dataset into the training set and validation set with a proportion of 0.85 : 0.15. As shown in Table s5, on the PhysioNet dataset, `AICare` deals with 34-dimension lab test data and achieves relative improvements of 7.7% in AUPRC, compared to the best baseline models.

Additional Interpretability Analysis

Additional Case Study on AI-Doctor Interaction System

On the patient detail page, users can view the patient's static baseline demographic information, dynamic trajectories of biomarkers, and prediction results. The system automatically displays the most *key* biomarkers that dominate the prediction results and provides the importance weights assigned by the model.

Case III: Patient Died of Sudden Death and Insufficient Dialysis (Fig. s4)

This case was diagnosed with diabetic nephropathy and initiated PD therapy. Fig. s4 shows the details of the previous visits and the results of the mortality risk assessment for this patient.

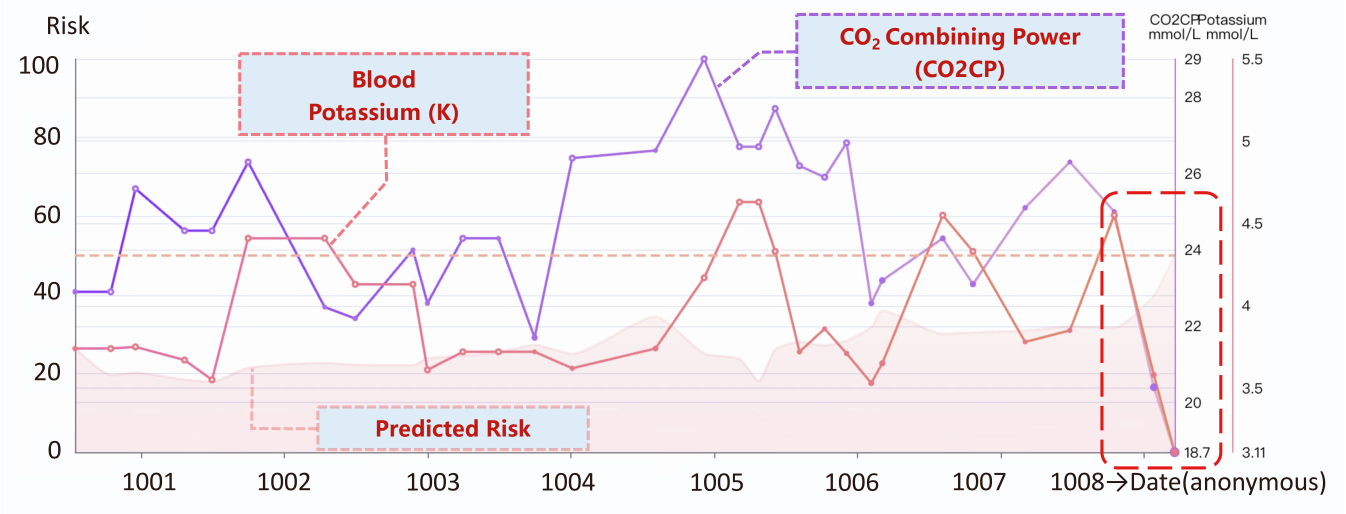

Figure s4: **Case Study III: Patient Died of Insufficient Dialysis and Sudden Heart Attack.** `AICare` pays most attention to CO2CP and Potassium for this patient. The health trajectory interactive visualization system is publicly deployed at `http://v.ai-care.top/A1`.

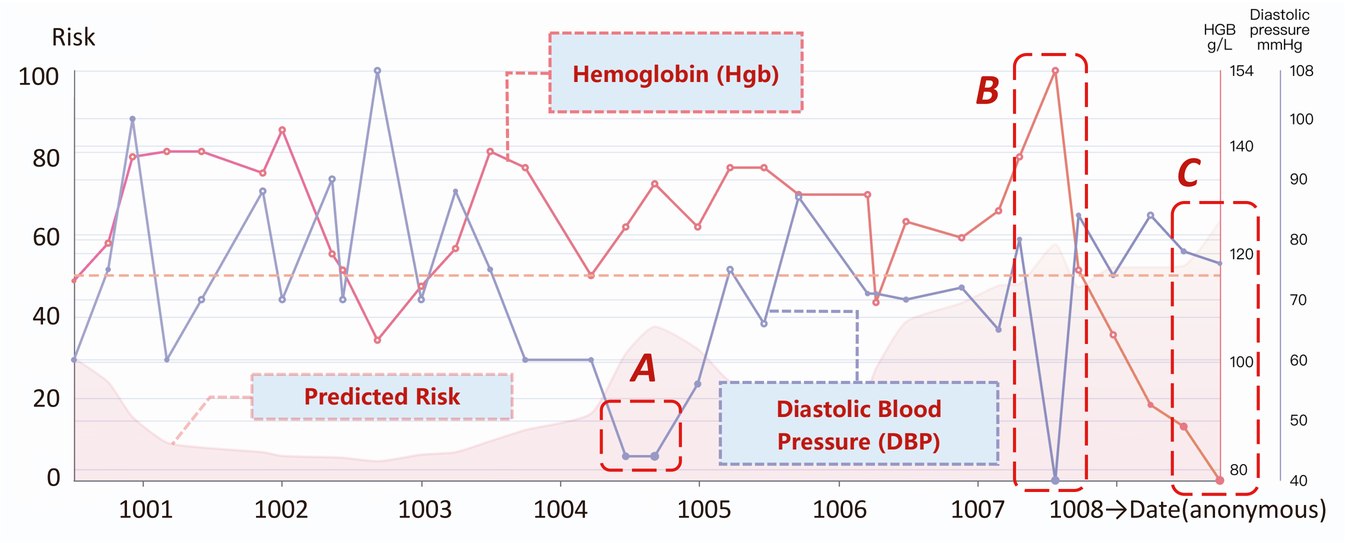

Figure s5: **Case Study IV: Patient Died of Gastrointestinal Bleeding.** `AICare` pays most attention to Diastolic Blood Pressure and Hemoglobin for this patient. The health trajectory interactive visualization system is publicly deployed at `http://v.ai-care.top/A3`.

The model believed that the patient's risk of death rose during the last two visits, indicating a deterioration of her health. The model assigned high attention weights to two medical features: carbon dioxide combining power (CO2CP) and potassium (K), due to the substantial decline of these two features at the last visits. `AICare` recommended focusing on the changes of carbon dioxide combining power (CO2CP) and potassium (K), where CO2CP decreased by 6.3 mmol/L rapidly (from 25.0 mmol/L to 18.7 mmol/L, 25.2% relative decrease), and K decreased by 1.44 mmol/L (from 4.55 mmol/L to 3.11 mmol/L, 31.6% relative decrease). The predicted mortality risk rapidly increased from 31.6 to 50.4 from September 1007 to March 1008.

This patient died on May, 1008. The cause of death was inadequate dialysis due to abdominal leakage of dialysate and sudden heart attack. The model sensed changes in important physiological indicators of the patient and provided early warning of possible risk factors for the patient. CO2CP was an indicator of acidosis, closely related to inadequate dialysis, and acidosis posed a health risk to the patient. The alert of potassium level could help physicians to give attention to it and treat hypokalemia by potassium supplement or hyperkalemia by necessary medical intervention.

Case IV: Patient Died of Gastrointestinal Bleeding (Fig. s5)

This case was diagnosed with glomerulonephritis and initiated PD therapy. There were three risk scores peaks during the whole PD treatment period. The first risk score peak (A) occurred on June, 1004. The risk score increased from 12.1 one year before, on July, 1003, to 37.53 on September, 1004. During this period, AI paid attention mainly to serum albumin (Please visit the link `http://v.ai-care.top/A3` for details.). On September, 1004, `AICare` found abnormal value changes in blood pressure (DBP dropped from 60 mmHg to 44 mmHg and SBP dropped from 100 mmHg to 96 mmHg) and paid much attention to DBP (26.3%) and SBP (12%) behind serum albumin (33.8%). The second risk score peak (B) was on July, 1007. The risk score increased to 57.59. The model found abnormal values of SBP (73mm Hg) and DBP (40 mm Hg). Attention was mainly given to SBP (28.1%) and DBP (24.3%). The third risk peak (C) was on September, 1008. The patient risk score increased to 63.37. The model found an abnormal decline and low hemoglobin value (from 92g/L to 78g/L). The highest attention (26.1%) was given to hemoglobin level, followed by 25.5% of attention to serum albumin (29.1g/L). The patient died of gastrointestinal bleeding on November, 1008. `AICare` accurately captured the abnormality of blood pressure level and hemoglobin. Hemoglobin level was one of the main manifestations of gastrointestinal bleeding causing this patient's death.

Additional Observation of Feature Importance Variation

1)-5) For analysis of albumin, diastolic blood pressure, systolic blood pressure, creatinine, and hemoglobin, please see the main text.

6) Chlorine (Cl) Fig.3-(d). As shown in Table 3, the importance weight of chlorine presents as a V-shaped curve with 96 mmol/L as the lowest turning point. In the 82-96 mmol/L range, `AICare` pays higher attention weight to chlorine level as the chlorine gets lower and predicts a poor prognosis, marked as red dots in Fig.3-(d). Meanwhile, in the range of 96-120 mmol/L, the model pays more attention to chlorine level as it gets higher and predicts a good prognosis. `AICare` recommends raising the chloride level to above and even higher than 96 mmol/L for most PD patients. To the best of our knowledge, the direct effect of chlorine on PD patient mortality has not been investigated by previous studies. Some studies find that blood chlorine level positively correlates with residual renal function[19]. The suggested level of blood chlorine by `AICare` is highly consistent with the traditional reference range of chlorine for outpatients, which is 96-106 mmol/L.

7) Urea Fig.3-(f). The importance variation learned by `AICare` presents an L-shaped curve

with a 20 mmol/L turning point. The model pays more attention to urea levels in the range of 4-20 mmol/L as the urea level decreases. When urea level drops below 10 mmol/L, the model provides more than 20% of attention weights, and patients in this range are more likely to suffer a high mortality risk (i.e., $\hat{y} > 0.5$, marked as red dots in Fig.3-(f)). However, it nearly occupies no attention for urea levels in the range of 20-44 mmol/L. AICare recommends maintaining urea level at 20 mmol/L or slightly higher for most PD patients. It will not bring much benefit if urea rises above 20 mmol/L. This may be because the urea level indicates the nutritional status of PD patients. The traditional recommended reference range of urea for outpatient clinics is 3.1-9 mmol/L, which is inconsistent with AICare's suggestions. The traditional recommended range is only suitable for normal outpatients without ESRD. To the best of our knowledge, the recommended reference range of urea for PD patients has not been analyzed by existing works based on end-to-end deep learning methods.

8) Calcium (Ca) Fig.3-(g). The importance variation of calcium presents as an L-shaped curve with a turning point of 2.5 mmol/L. For calcium in the 1-2.5 mmol/L range, AICare pays more attention to calcium and tends to make adverse predictions as the calcium level decreases. For calcium over 2.5 mmol/L, the model pays nearly no attention to it. AICare recommends maintaining calcium level at least 2.5 mmol/L, while increasing calcium above 2.5 mmol/L may not bring much more benefits. The traditional recommended calcium reference range for outpatient clinics is 2.25-2.75 mmol/L, which is consistent with AICare's suggestions.

9) Sodium (Na) Fig.3-(h). The importance variation of sodium presents as an L-shaped curve with a turning point of 135.5 mmol/L. For sodium level in the 121-135.5 mmol/L, AICare pays more attention to sodium and tends to make adverse predictions as the sodium level decreases. This is consistent with the previous study that an increased mortality rate associated with hyponatremia in PD patients[20]. Moreover, a study in time-dependent analysis shows that, in PD patients, lower time-dependent and baseline sodium levels were independently associated with higher death risk[21]. Yet, for sodium level over 135.5 mmol/L, the model pays nearly no attention to it. Thus, AICare recommends maintaining sodium level at least 135.5 mmol/L, while increasing sodium above 135.5 mmol/L may not bring much more benefits. The traditional recommended reference range of sodium for outpatient clinics is 135-145 mmol/L, which is highly consistent with AICare's suggestions.

10) Potassium (K) Fig.4-(i). AICare pays more attention and tends to make adverse predictions as the potassium level decreases for potassium level in the range of 2.3-4 mmol/L. For potassium level over 4 mmol/L, the model pays nearly no attention to it. AICare recommends maintaining potassium level at 4 least mmol/L, but further improvement may not bring benefits. The traditional recommended reference range of potassium for outpatient clinics is 3.5-5.5 mmol/L. Previous studies have also reported the association between low potassium level and PD patients' poor outcomes[22,23].

11) Phosphorus (P) Fig.4-(j). AICare pays more attention and makes adverse predictions as the phosphate level decreases for phosphate level in the range of 0.5-1.5 mmol/L. AI recommends maintaining phosphate level at least 1.5 mmol/L, while further increase may not bring benefits. The traditional recommended reference range of phosphate for outpatient clinics is 1.1-1.3 mmol/L, which is **inconsistent** with AICare's suggestions. However, this is **consistent** with the KDOQI guidelines for preventing hyperphosphatemia in ESRD patients, which points out that the normal range of phosphate is 0.85-1.51 mmol/L. Low serum phosphorus is usually associated with poor dietary intake, and the association between low serum phosphorus and poor clinical outcome has been reported previously[24].

12) Carbon Dioxide Combining Power (CO2CP) Fig.4-(k). AICare pays more attention and makes adverse predictions as the CO2CP level decreases for CO2CP level in the range of 10.5-25 mmol/L. Yet, for CO2CP level over 25 mmol/L, the model pays nearly no attention to it. Thus, AICare recommends maintaining the CO2CP level at least 25 mmol/L. However, further

improvement on CO2CP does not help reduce mortality risk. The traditional recommended reference range of CO2CP for outpatient clinics is 20-29 mmol/L. According to previous studies, low serum bicarbonate indicated acidosis in PD patients, which is associated with catabolism, malnutrition, and poor outcome[25,26].

13) Body Weight Fig.4-(m). `AICare` pays more attention and makes adverse predictions as the body weight decreases for body weight in the range of 29-59 kg. For body weight over 59 kg, the model pays nearly no attention to it. `AICare` recommends maintaining body weight at 60 kg for most PD patients. However, the further improvement of body weight may not bring benefits. Malnutrition and low body weight are associated with higher mortality in peritoneal dialysis (PD)[27,28].

14) Glucose Fig.4-(n). `AICare` pays more attention and makes good predictions as the glucose level decreases for glucose level in the range of 1-6 mmol/L. For glucose level over 6 mmol/L, the model pays nearly no attention to it. Thus, AI recommends maintaining glucose level not exceeding 6 mmol/L. The traditional recommended reference range of glucose for outpatient clinics is 3.9-6.1 mmol/L, which is consistent with `AICare`'s suggestions.

15) Hypersensitive C-Reactive Protein (Hs-CRP) Fig.4-(o). Hs-CRP is a highly skewed L-shaped curve with a 16 mg/L turning point. For hs-CRP level 0-16 mg/L, `AICare` pays more attention and makes good predictions as the hs-CRP level decreases. For hs-CRP level over 16 mg/L, the model pays nearly no attention to it. Thus, `AICare` recommends maintaining the hs-CRP level not exceeding 16 mg/L. The traditional recommended reference range of hs-CRP for outpatient clinics is 0.5-10 mg/L, which is almost consistent with `AICare`'s suggestions.

16) White Blood Cell Count (WBC) Fig.4-(p). WBC is considered by `AICare` as the most eccentric clinical feature included in the dataset. For most patients, the importance weights assigned to WBC is below 1%. The model believes that WBC is not a crucial feature for ESRD patients when conducting 1-year mortality prediction.

As a preliminary conclusion drawn from the analysis of a specific peritoneal dialysis patient dataset based on deep learning methods, we suggest that readers use the above suggestions as inspiration for future work and clinical considerations, but do not directly implement specific recommendations for such indicators to clinical patients.

Related Work

Over the past ten years, there has been a massive explosion in the amount of digital information stored in electronic medical records, which opens a door for researchers to make secondary use of these records for various clinical applications[29–34]. At the same time, with the development of artificial intelligence, machine learning and deep learning-based models have shown the capability to perform renal-disease-related clinical predictions, including acute kidney injury risk prediction[6,8,35,36], graft loss prediction[37] and mortality prediction[8,10,37–42]. For instance, Bai et al.[43] proposed a study to assess the feasibility of machine learning (ML) in predicting the risk of end-stage kidney disease (ESKD) for patients with CKD.[44] developed a deep-learning-based prediction model for end-stage kidney disease (ESKD) in patients with primary immunoglobulin A nephropathy (IgAN). Noh et al.[10] conducted the 5-year mortality risk prediction task for peritoneal dialysis patients using the decision tree model. Xu et al.[35], Ravizza et al.[12] and Chaudhuri et al.[45] utilized patient static information to conduct the prediction for the progression of renal diseases, such as AKI, CKD and hospitalization. Besides, Akbilgic et al.[38], Liu et al.[39], Zhou et al.[40], Radovic et al.[41] and Kang et al.[42] used different ML-based methods to predict the mortality risk of patients with kidney-related diseases.

However, there are still some critical issues that have not yet been thoroughly addressed by existing work in terms of the following three issues.

I_1: **Perform dynamic mortality prediction at each follow-up visit based on the effective utilization of both sequential medical records and the baseline demographic information.** Most above-mentioned existing renal disease-related works only utilize static records. Such models cannot learn non-linear progression patterns from high-dimensional longitudinal EMR datasets, capturing the health status variation trajectory, limiting their prediction performance and applications in quality improvement initiatives or data-driven clinical decision-making processes. It requires significant efforts from clinicians, healthcare institutions and model developers to collect data and build longitudinal models for such long-term predictive tasks.

Some works attempted to model the dynamic information of patients. For example, Makino et al.[46] constructed a predictive model for diabetic kidney diseases (DKD) using AI, processing natural language and longitudinal data of diabetes patients. Rank et al.[36] developed a deep-learning-based real-time algorithm to predict postoperative AKI prior to the onset of symptoms and complications.Tomavsev et al.[6] and Hyland et al.[11] proposed DL-based models to conduct dynamic monitoring for the risk of AKI and circulatory failure on in-hospital patients with kidney disease. These work dynamically predict patients' risk through different time-series models based on deep learning and realize the dynamic monitoring of the health status of patients with kidney disease. However, the baseline data of patients with renal disease is also essential in diagnosing and treating. These researches have limitations in combining the static baseline information and dynamic data of patients, thence have difficulties comprehensively integrating the patient information for evaluation.

Thence, Srinivas et al.[37] combined the static and dynamic data of patients who received a kidney transplant and conducted the graft loss and mortality prediction via LR. Sung et al.[8] have used biLSTM-FC to perform the clinical event prediction (death, sepsis, and acute kidney injury), where biLSTM and fully connected layers are employed to embed the dynamic features and static features correspondingly. However, these works briefly concatenate the static and sequential information by the hidden units. The static information does not guide the individual health status representation learning or help the model adaptively assign weights to the features.

I_2: **Provide fine-grained interpretability for each patient individually by selecting key features which contribute the most to the mortality prediction (patient-level interpretability) and achieve high prediction performance simultaneously.** Although deep learning has achieved huge success in many domains, lack-of-explainability remains one serious drawback for the neural network. An interpretable model is essential for clinical decision support applications as the predictive results need to be understood by clinicians to adopt individualized treatment and extract medical knowledge. However, the model interpretability has not been fully discussed in most renal disease-related works. The decision-making process in such deep models is a black box and fails to provide human-understandable interpretability.

Several researchers have explored the interpretability in the medical feature via tree-based strategy. For example, Noh et al.[10] assessed mortality risk prediction in PD patients using decision tree algorithms. Akbilgic et al.[38] implemented a random forest method to predict outcomes of ESRD patients after dialysis initiation. However, the prediction performance of these static information-based methods is limited due to the deficiency of effective advanced clinical feature extraction.

Some recent works apply the SHapley Additive exPlanations (SHAP)[7,11], feature permutation[40,41], and inverse analysis[46] strategies to provide the post-hoc interpretability. For example, Makino et al. generated the time-series data pattern by inverse analysis. However, these interpreting methods usually only provide coarse-grained analysis and may face a difficult trade-off between the network complexity and prediction performance. As a result, it is still challenging to provide satisfying interpretability and achieve high prediction performance simultaneously.

I_3: **Adaptively analyze the importance of each feature along with the variation of its value (feature-level interpretability) to provide medical advice and extract knowledge.** The

interpretability shown in most of the existing EMR analysis works mainly focuses on tree-based interpretability and some forms of post-hoc interpretability. The tree-based analysis can only provide a fixed decision process for all patients and face a deficiency in sequential information utilization. To the best of our knowledge, none of the existing PD/HD/ESRD-related works explicitly provide the adaptive feature importance, analyze the changes of feature importance with its values, and extract medical advice based on ante-hoc interpretability in a deep end-to-end model.

References

1. Hu, J., Shen, L., and Sun, G. Squeeze-and-excitation networks. In: *Proceedings of the IEEE Conference on Computer Vision and Pattern Recognition* (2018):(7132–7141).

2. Kingma, D. P., and Ba, J. (2014). Adam: A method for stochastic optimization. arXiv preprint arXiv:1412.6980.

3. Meyer, A., Zverinski, D., Pfahringer, B., Kempfert, J., Kuehne, T., Sündermann, S. H., Stamm, C., Hofmann, T., Falk, V., and Eickhoff, C. (2018). Machine learning for real-time prediction of complications in critical care: a retrospective study. The Lancet Respiratory Medicine *6*, 905–914.

4. Vaswani, A., Shazeer, N., Parmar, N., Uszkoreit, J., Jones, L., Gomez, A. N., Kaiser, Ł., and Polosukhin, I. Attention is all you need. In: *Advances in neural information processing systems* (2017):(5998–6008).

5. Nitski, O., Azhie, A., Qazi-Arisar, F. A., Wang, X., Ma, S., Lilly, L., Watt, K. D., Levitsky, J., Asrani, S. K., Lee, D. S. et al. (2021). Long-term mortality risk stratification of liver transplant recipients: real-time application of deep learning algorithms on longitudinal data. The Lancet Digital Health *3*, e295–e305.

6. Tomašev, N., Glorot, X., Rae, J. W., Zielinski, M., Askham, H., Saraiva, A., Mottram, A., Meyer, C., Ravuri, S., Protsyuk, I. et al. (2019). A clinically applicable approach to continuous prediction of future acute kidney injury. Nature *572*, 116–119.

7. Thorsen-Meyer, H.-C., Nielsen, A. B., Nielsen, A. P., Kaas-Hansen, B. S., Toft, P., Schierbeck, J., Strøm, T., Chmura, P. J., Heimann, M., Dybdahl, L. et al. (2020). Dynamic and explainable machine learning prediction of mortality in patients in the intensive care unit: a retrospective study of high-frequency data in electronic patient records. The Lancet Digital Health *2*, e179–e191.

8. Sung, M., Hahn, S., Han, C. H., Lee, J. M., Lee, J., Yoo, J., Heo, J., Kim, Y. S., Chung, K. S. et al. (2021). Event prediction model considering time and input error using electronic medical records in the intensive care unit: Retrospective study. JMIR medical informatics *9*, e26426.

9. Yan, L., Zhang, H.-T., Goncalves, J., Xiao, Y., Wang, M., Guo, Y., Sun, C., Tang, X., Jing, L., Zhang, M. et al. (2020). An interpretable mortality prediction model for covid-19 patients. Nature Machine Intelligence (1–6).

10. Noh, J., Yoo, K. D., Bae, W., Lee, J. S., Kim, K., Cho, J.-H., Lee, H., Kim, D. K., Lim, C. S., Kang, S.-W. et al. (2020). Prediction of the mortality risk in peritoneal dialysis patients using

machine learning models: a nation-wide prospective cohort in korea. Scientific reports *10*, 1–11.

11. Hyland, S. L., Faltys, M., Hüser, M., Lyu, X., Gumbsch, T., Esteban, C., Bock, C., Horn, M., Moor, M., Rieck, B. et al. (2020). Early prediction of circulatory failure in the intensive care unit using machine learning. Nature medicine *26*, 364–373.

12. Ravizza, S., Huschto, T., Adamov, A., Böhm, L., Büsser, A., Flöther, F. F., Hinzmann, R., König, H., McAhren, S. M., Robertson, D. H. et al. (2019). Predicting the early risk of chronic kidney disease in patients with diabetes using real-world data. Nature medicine *25*, 57–59.

13. Ma, T., Xiao, C., and Wang, F. Health-atm: A deep architecture for multifaceted patient health record representation and risk prediction. In: *Proceedings of the 2018 SIAM International Conference on Data Mining*. SIAM (2018):(261–269).

14. Davis, J., and Goadrich, M. The relationship between precision-recall and roc curves. In: *Proceedings of the 23rd international conference on Machine learning*. ACM (2006):(233–240).

15. Choi, E., Xiao, C., Stewart, W., and Sun, J. Mime: Multilevel medical embedding of electronic health records for predictive healthcare. In: *Advances in Neural Information Processing Systems* (2018):(4547–4557).

16. Wikipedia contributors. Cerebrovascular disease — Wikipedia, the free encyclopedia (2019). URL: `https://en.wikipedia.org/w/index.php?title=Cerebrovascular_disease&oldid=876465095` [Online; accessed 3-February-2019].

17. Zhao, X., Niu, Q., Gan, L., Hou, F. F., Liang, X., Ni, Z., Chen, Y., Zhao, J., Bieber, B., Robinson, B. et al. (2021). Baseline data report of the china dialysis outcomes and practice patterns study (dopps). Scientific reports *11*, 873.

18. Reyna, M. A., Josef, C. S., Jeter, R., Shashikumar, S. P., Westover, M. B., Nemati, S., Clifford, G. D., and Sharma, A. (2019). Early prediction of sepsis from clinical data: the physionet/computing in cardiology challenge 2019. Critical Care Medicine.

19. Li, L., Liang, W., Ye, T., Chen, Z., Zuo, X., Du, X., Qian, K., Zhang, C., Hu, X., Li, J. et al. (2016). The association between nutritional markers and biochemical parameters and residual renal function in peritoneal dialysis patients. PLoS One *11*, e0156423.

20. Al-Chidadi, A., Nitsch, D., and Davenport, A. (2017). The effect of serum sodium on survival in patients treated by peritoneal dialysis in the united kingdom. Peritoneal dialysis international *37*, 70–77.

21. Ravel, V. A., Streja, E., Mehrotra, R., Sim, J. J., Harley, K., Ayus, J. C., Amin, A. N., Brunelli, S. M., Kovesdy, C. P., Kalantar-Zadeh, K. et al. (2017). Serum sodium and mortality in a national peritoneal dialysis cohort. Nephrology Dialysis Transplantation *32*, 1224–1233.

22. Davies, S. J., Zhao, J., Morgenstern, H., Zee, J., Bieber, B., Fuller, D. S., Sloand, J. A., Vychytil, A., Kawanishi, H., Johnson, D. W. et al. (2021). Low serum potassium levels and clinical outcomes in peritoneal dialysis—international results from pdopps. Kidney international reports *6*, 313–324.

23. Szeto, C.-C., Chow, K.-M., Kwan, B. C.-H., Leung, C.-B., Chung, K.-Y., Law, M.-C., and Li, P. K.-T. (2005). Hypokalemia in chinese peritoneal dialysis patients: prevalence and prognostic implication. American journal of kidney diseases *46*, 128–135.

24. Liu, C.-T., Lin, Y.-C., Lin, Y.-C., Kao, C.-C., Chen, H.-H., Hsu, C.-C., and Wu, M.-S. (2017). Roles of serum calcium, phosphorus, pth and alp on mortality in peritoneal dialysis patients: a nationwide, population-based longitudinal study using twrds 2005–2012. Scientific reports *7*, 1–9.

25. Szeto, C., and Lal, K. (1998). Metabolic acidosis and nutritional status of patients receiving continuous ambulatory peritoneal dialysis (capd). The International journal of artificial organs *21*, 192–195.

26. Kang, D.-H. (1999). Metabolic acidosis as a catabolic factor in peritoneal dialysis patients. Peritoneal dialysis international *19*, 304–308.

27. Imam, T. H., Shi, J. M., Yi, D. K., and Yang, S.-J. (2021). Long-term peritoneal dialysis is associated with a decrease in body weight. Clinical kidney journal.

28. Taylor, P., Keshaviah, R., and Beecroft, M. L. (1996). Adequacy of dialysis and nutrition in continuous peritoneal dialysis: Association with clinical outcomes1. J, Am. Soc. Nephrol *7*, 198–207.

29. Ma, L., Gao, J., Wang, Y., Zhang, C., Wang, J., Ruan, W., Tang, W., Gao, X., and Ma, X. Adacare: Explainable clinical health status representation learning via scale-adaptive feature extraction and recalibration. In: *Thirty-Fourth AAAI Conference on Artificial Intelligence* (2020):.

30. Ma, L., Zhang, C., Wang, Y., Ruan, W., Wang, J., Tang, W., Ma, X., Gao, X., and Gao, J. Concare: Personalized clinical feature embedding via capturing the healthcare context. In: *Thirty-Fourth AAAI Conference on Artificial Intelligence* (2020):.

31. Gao, J., Xiao, C., Wang, Y., Tang, W., Glass, L. M., and Sun, J. Stagenet: Stage-aware neural networks for health risk prediction. In: *Proceedings of The Web Conference 2020* (2020):(530–540).

32. Gao, J., Xiao, C., Glass, L. M., and Sun, J. (2020). Dr. agent: Clinical predictive model via mimicked second opinions. Journal of the American Medical Informatics Association *27*, 1084–1091.

33. Gao, J., Yang, C., Heintz, J., Barrows, S., Albers, E., Stapel, M., Warfield, S., Cross, A., and Sun, J. (2022). Medml: fusing medical knowledge and machine learning models for early pediatric covid-19 hospitalization and severity prediction. Iscience *25*.

34. Ma, L., Ma, X., Gao, J., Jiao, X., Yu, Z., Zhang, C., Ruan, W., Wang, Y., Tang, W., and Wang, J. Distilling knowledge from publicly available online emr data to emerging epidemic for prognosis. In: *Proceedings of the Web Conference 2021* (2021):(3558–3568).

35. Xu, Z., Luo, Y., Adekkanattu, P., Ancker, J. S., Jiang, G., Kiefer, R. C., Pacheco, J. A., Rasmussen, L. V., Pathak, J., and Wang, F. Stratified mortality prediction of patients with acute kidney injury in critical care. In: *MEDINFO 2019: Health and Wellbeing e-Networks for All* (462–466). IOS Press (2019):(462–466).

36. Rank, N., Pfahringer, B., Kempfert, J., Stamm, C., Kühne, T., Schoenrath, F., Falk, V., Eickhoff, C., and Meyer, A. (2020). Deep-learning-based real-time prediction of acute kidney injury outperforms human predictive performance. NPJ digital medicine *3*, 1–12.

37. Srinivas, T., Taber, D., Su, Z., Zhang, J., Mour, G., Northrup, D., Tripathi, A., Marsden, J., Moran, W., and Mauldin, P. (2017). Big data, predictive analytics, and quality improvement in kidney transplantation: a proof of concept. American Journal of Transplantation *17*, 671–681.

38. Akbilgic, O., Obi, Y., Potukuchi, P. K., Karabayir, I., Nguyen, D. V., Soohoo, M., Streja, E., Molnar, M. Z., Rhee, C. M., Kalantar-Zadeh, K. et al. (2019). Machine learning to identify dialysis patients at high death risk. Kidney international reports *4*, 1219–1229.

39. Liu, J., Wu, J., Liu, S., Li, M., Hu, K., and Li, K. (2021). Predicting mortality of patients with acute kidney injury in the icu using xgboost model. Plos one *16*, e0246306.

40. Zhou, Q., You, X., Dong, H., Lin, Z., Shi, Y., Su, Z., Shao, R., Chen, C., and Zhang, J. (2021). Prediction of premature all-cause mortality in patients receiving peritoneal dialysis using modified artificial neural networks. Aging (Albany NY) *13*, 14170.

41. Radović, N., Prelević, V., Erceg, M., and Antunović, T. (2022). Machine learning approach in mortality rate prediction for hemodialysis patients. Computer Methods in Biomechanics and Biomedical Engineering *25*, 111–122.

42. Kang, M. W., Kim, J., Kim, D. K., Oh, K.-H., Joo, K. W., Kim, Y. S., and Han, S. S. (2020). Machine learning algorithm to predict mortality in patients undergoing continuous renal replacement therapy. Critical Care *24*, 1–9.

43. Bai, Q., Su, C., Tang, W., and Li, Y. (2022). Machine learning to predict end stage kidney disease in chronic kidney disease. Scientific reports *12*, 1–8.

44. Schena, F. P., Anelli, V. W., Trotta, J., Di Noia, T., Manno, C., Tripepi, G., D'Arrigo, G., Chesnaye, N. C., Russo, M. L., Stangou, M. et al. (2021). Development and testing of an artificial intelligence tool for predicting end-stage kidney disease in patients with immunoglobulin a nephropathy. Kidney International *99*, 1179–1188.

45. Chaudhuri, S., Han, H., Usvyat, L., Jiao, Y., Sweet, D., Vinson, A., Steinberg, S. J., Maddux, D., Belmonte, K., Brzozowski, J. et al. (2021). Machine learning directed interventions associate with decreased hospitalization rates in hemodialysis patients. International Journal of Medical Informatics *153*, 104541.

46. Makino, M., Yoshimoto, R., Ono, M., Itoko, T., Katsuki, T., Koseki, A., Kudo, M., Haida, K., Kuroda, J., Yanagiya, R. et al. (2019). Artificial intelligence predicts the progression of diabetic kidney disease using big data machine learning. Scientific reports *9*, 1–9.
